# “Anti‐Electrostatic” Halogen Bonding†

**DOI:** 10.1002/anie.202003083

**Published:** 2020-04-30

**Authors:** Jana M. Holthoff, Elric Engelage, Robert Weiss, Stefan M. Huber

**Affiliations:** ^1^ Fakultät für Chemie und Biochemie Ruhr-Universität Bochum Universitätsstraße 150 44801 Bochum Germany; ^2^ Institut für Organische Chemie Friedrich-Alexander-Universität Erlangen-Nürnberg Henkestraße 42 91054 Erlangen Germany

**Keywords:** density-functional calculations, electrostatics, halogen bonding, hydrogen bonding, noncovalent interactions

## Abstract

Halogen bonding is often described as being driven predominantly by electrostatics, and thus adducts between anionic halogen bond (XB) donors (halogen‐based Lewis acids) and anions seem counterintuitive. Such “anti‐electrostatic” XBs have been predicted theoretically but for organic XB donors, there are currently no experimental examples except for a few cases of self‐association. Reported herein is the synthesis of two negatively charged organoiodine derivatives that form anti‐electrostatic XBs with anions. Even though the electrostatic potential is universally negative across the surface of both compounds, DFT calculations indicate kinetic stabilization of their halide complexes in the gas phase and particularly in solution. Experimentally, self‐association of the anionic XB donors was observed in solid‐state structures, resulting in dimers, trimers, and infinite chains. In addition, co‐crystals with halides were obtained, representing the first cases of halogen bonding between an organic anionic XB donor and a different anion. The bond lengths of all observed interactions are 14–21 % shorter than the sum of the van der Waals radii.

## Graphical abstract

**Coming together**: Two negatively charged organoiodine derivatives are shown to form anti‐electrostatic halogen bonds (XBs) with anions. Even though the electrostatic potential is universally negative across the surface of both compounds, DFT calculations indicate kinetic stabilization of their halide complexes. Experimentally, self‐association of the anionic XB donors is observed. Co‐crystals with halides were obtained and featured XB adducts between two or even three anions.

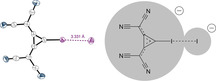

## Introduction

In recent years, halogen bonding (XBs),^1^ the noncovalent interaction between electrophilic halogen substituents and Lewis bases (LBs), has found widespread use in many fields of chemistry, including crystal engineering,^2^ anion recognition,^3^ and organocatalysis.^4^ The somewhat counterintuitive attraction between the seemingly electron‐rich halogen substituent and Lewis bases was originally described by Mulliken^5^ as a form of n→σ*‐type orbital interaction.^6^, ^7^ Later, an electrostatic model became very popular, which is based on the σ‐hole,^8^ a region of positive electrostatic potential at the elongation of the R−X bond (X=halogen). Sometimes, the interaction has even been described as “electrostatically driven”.^9^ Consequently, XB donors (halogen‐based Lewis acids) are typically either neutral^10^ or cationic,^11^ and the latter bind particularly strongly to anionic Lewis bases because of charge assistance.^12^ In contrast, the coordination of such substrates with anionic XB donors does not seem sensible based on an electrostatic reasoning.

It is noteworthy that for other interactions, a growing number of experimental evidence for adducts between ions of like charge were reported, for example, for guanidinium ions in water,^13^ biomolecules like oligopeptides,^14^ metastable colloidal crystallites,^15^ or for ionic liquids with weakly coordinating counterions.^16^, ^17^, ^18^, ^19^ In the latter example, cationic imidazolium species form clusters which are stabilized by cooperative hydrogen bonds (HB). The latter is another type of interaction that is considered to be primarily electrostatic in nature,^20^ but for which n→σ* orbital interactions are often also non‐negligible.^21^ This was demonstrated vividly in a study by Weinhold and Klein^22^ on cation–cation and anion–anion complexes that showed unusual kinetic stability, thereby challenging the seemingly generally accepted electrostatic model of HBs. This first report of so‐called anti‐electrostatic hydrogen bonds (AEHBs) was followed by numerous theoretical studies^23^, ^24^ as well as experimental evidence.^16^, ^17^, ^18^, ^19^, ^25^

For halogen bonding, the first theoretical studies on anti‐electrostatic XBs (AEXBs)^26^, ^27^ appeared only very recently. For organic compounds, the only experimental systems that could be considered to contain AEXBs are a handful of cases in which anionic organohalogen compounds show self‐association in solid‐state structures (even though in these studies, the contacts were not interpreted as AEXBs and were not analyzed further).^28^ In addition, several halometallates exhibit halogen–halogen contacts in the solid state either by self‐association or by coordination to (poly)halides.^29^ These contacts are typically very weak, however, and it is unclear whether they should be characterized as XB.^30^ Polyhalides like [Cl⋅⋅⋅I−I⋅⋅⋅Cl]^2−^, which form linear XB adducts in crystal structures if the terminal halides are coordinated by further interactions, may also feature AEXBs.^31^ However, it is debatable whether these complexes feature direct anion–anion interactions or if a better description would be the interaction of two anions with the same neutral XB donor (I_2_).

Thus, to the best of our knowledge, there are no examples of AEXBs between organic XB donors and structurally different anions, that is, AEXBs which are not based on self‐association. Herein, we present anionic iodocyclopropenium and iodoimidazole derivatives which form such complexes with halides for the first time (next to several variants of self‐association).

## Results and Discussion

**Figure 1:**
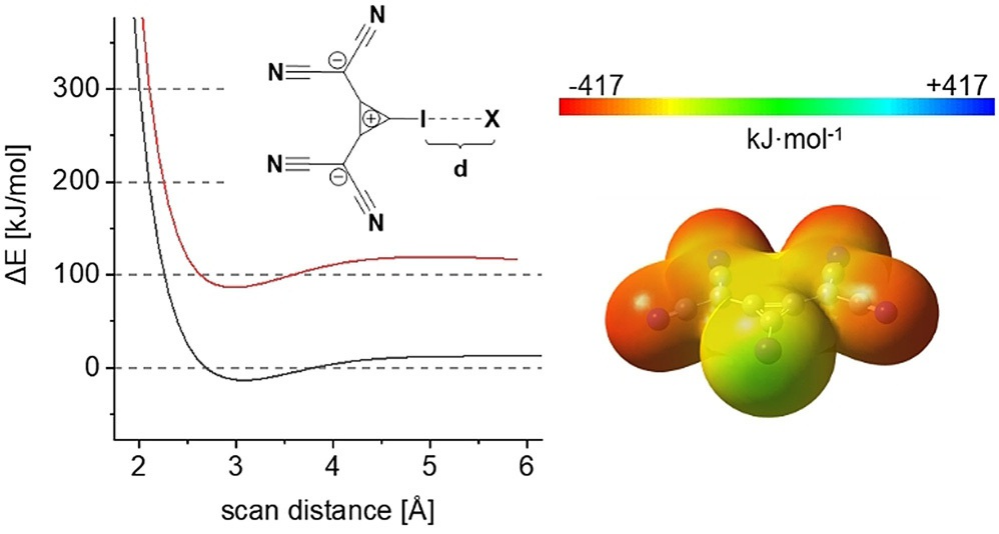
Left: Energy profile of the scan of the I⋅⋅⋅Cl^−^ bond distance for the **1**⋅Cl^−^ adduct in the gas phase (red) and in acetonitrile (black). Right: Electrostatic potential of **1** mapped on the 0.001 electron/Bohr^3^ isosurface of electronic density. In elongation of the C−I bond, a region of less negative electrostatic potential (*V*_S,max_=−80.36 kJ mol^−1^) can be seen (green color).

**Scheme 1:**
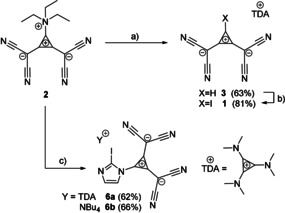
Syntheses of **1** starting from **2**, which can be converted into **3**. a) 1. NaBH_4_, MeOH, 0 °C→r.t., 2. H_2_O, TDACl; b) 1,3‐diiodo‐5,5‐dimethylhydantoin (**4**), DCM, r.t., 2 h; c) 1. 2‐iodoimidazole (**5**), LDA, THF, −78 °C, 2. addition of **2** in acetonitrile, −78 °C→r.t., 3. TDACl or NBu_4_Cl, H_2_O.

In the context of work directed at other goals, we became interested in the iodinated bis(dicyanomethylene)cyclopropanid derivate **1** (Scheme 1) and it soon became apparent that this type of compound is an ideal starting point to study AEXBs. First, DFT calculations were performed at the M06‐2X/def2‐TZVP‐level.^32^ The thus‐obtained electrostatic potential on the surface of the anion **1** shows that the negative charge is distributed over the whole molecule and that no region of positive electrostatic potential can be found (Figure 1, right), even though there is a region of less negative electrostatic potential in elongation of the C−I bond.

To test the ability of **1** to act as a halogen‐bonding Lewis acid, halides, X^−^ (X=Cl and I), were placed in elongation of the C−I bond and energy profiles scanning the C−I⋅⋅⋅X^−^ distances were obtained (see for example, Figure 1 left). Both calculations yielded minima corresponding to XB adducts with well depths (i.e., energies of the minima vs. highest energies at longer distances) of −16 kJ mol^−1^ (X=I) and −33 kJ mol^−1^ (X=Cl). Halogen bonding is indicated by either the I⋅⋅⋅I (3.5 Å) or I⋅⋅⋅Cl (3.0 Å) bond distances, which are markedly shorter than the sum of the van der Waals (vdW) radii^34^ (3.96 Å/3.73 Å) and the high directionality of the interaction (with C‐I⋅⋅⋅X angles of 180°). The overall binding energies (Δ*E*), enthalpies (Δ*H*), and Gibbs free enthalpies (Δ*G*) of the complexes were still markedly positive, however (Table 1, entries 1 and 2; see Table S20 in the Supporting Information). This outcome is not a surprise for a system consisting of two anions in the gas phase, and is in line with calculations on anionic systems reported in literature.^24^, ^27^ In contrast, when calculations were performed in acetonitrile (with the SMD18^35^ solvation model), negative binding energies for both iodide and chloride were found (Table 1, entries 3 and 4 and Figure 1). The corresponding Gibbs free energies were either very slightly positive (0.6 kJ mol^−1^ for **1**⋅⋅⋅I^−^) or even negative (−2.9 kJ mol^−1^ for **1**⋅⋅⋅Cl^−^), suggesting that these AEXBs could be stable in solution.

**Table 1:** Geometric parameters (I/H⋅⋅⋅LB distances in Å, ∡_C‐I/H⋅⋅⋅LB_ angles in °), well depths, binding energies and Gibb's free energies (with low‐frequency entropy corrections)^33^ [all in kJ mol^−1^] for minima corresponding to halogen/hydrogen bonding adducts. Calculations were performed in the gas phase, unless stated otherwise (“_solv_”) and the corresponding cations were omitted.

	Complex	*d*_Y⋅⋅⋅LB_	∡_C‐Y⋅⋅⋅LB_	Well depth	Δ*E*	Δ*G*
1	**1**⋅⋅⋅I^−^	3.5	180	−16	108	136
2	**1**⋅⋅⋅Cl^−^	3.0	180	−33	87	117
3	**1**⋅⋅⋅I^−^_solv_^[a]^	3.5	178	−23	−10	0.6
4	**1**⋅⋅⋅Cl^−^_solv_^[a]^	3.1	180	−26	−14	−1.9
5	**6**⋅⋅⋅Cl^−^	3.0	171	−24	111	142
6	**6**⋅⋅⋅I^−^	3.6	157	−3.0	126	158
7	**6**⋅⋅⋅I^−^_solv[_^[a]^	3.6	176	−18	6.7	11
8	**6**⋅⋅⋅Cl^−^_solv_^[a]^	3.0	176	−21	6.7	5.7
9	**6**⋅⋅⋅**6**_1XB_^[b]^	3.0	174	−13	80	141
10	**6**⋅⋅⋅**6**_2XB_^[c]^	3.0	171	–	89	152
11	**1**⋅⋅⋅**1**	3.1	177	−2.1	101	154
12	**1**⋅⋅⋅**1**_solv_^[a]^	3.0	180	−14	−14	45
13	**3**⋅⋅⋅I^−^	3.3	180	−0.03	142	172
14	**3**⋅⋅⋅Cl^−^	2.4	180	−6.7	135	166
15	**3**⋅⋅⋅I^−^_solv_^[a]^	3.0	180	−2.1	−1.5	−53
16	**3**⋅⋅⋅Cl^−^_solv_^[a]^	2.5	180	−5.2	9.3	−41

[a] Calculated with SMD18 using parameters for acetonitrile. [b] This dimeric structure features one XB contact as found for A⋅⋅⋅C in the crystal structure of **6 a** (see Figure 5). [c] A scan for the dimeric structure as found in the crystal structure of **6 b** (see Figure 4) was not possible. In case of **6**⋅⋅⋅**6**_1XB_, almost identical energies were obtained when calculations were performed either on the crystal structure geometry or the optimized minimum. Therefore, we used the geometries found in the crystal structure as minimum structure for further optimization and energy calculation.

Obviously, in the complexes discussed here, various electronic components contribute either favorably or unfavorably to the overall interaction energy: Pauli repulsion and electrostatic repulsion (resulting from the like charges) act against orbital interactions (n→σ*), polarization, and dispersion as attractive forces. The electrostatic repulsion will be lowered by counterions (which were not considered in these calculations) as well as by the dielectric environment of polar solvents (and individual interactions by the solvent molecules). In suitable cases like **1**⋅⋅⋅Cl^−^, the attractive forces, taken together, may overcome the repulsive components to form stable XB adducts, as indicated by our calculations.

With these promising calculations in hand, we synthesized the XB donor **1** starting from the inner salt **2**, which was first reported by Fukunaga and is known to react with nucleophiles under release of triethylamine.^36^ Since the direct conversion into the iodinated species failed using iodide as nucleophile, the corresponding H‐analogue **3** (Scheme 1) was chosen as precursor. This compound was already reported by Seitz et al. who described an eight‐step synthesis with an overall yield of about 5 %.^37^

**Figure 2:**
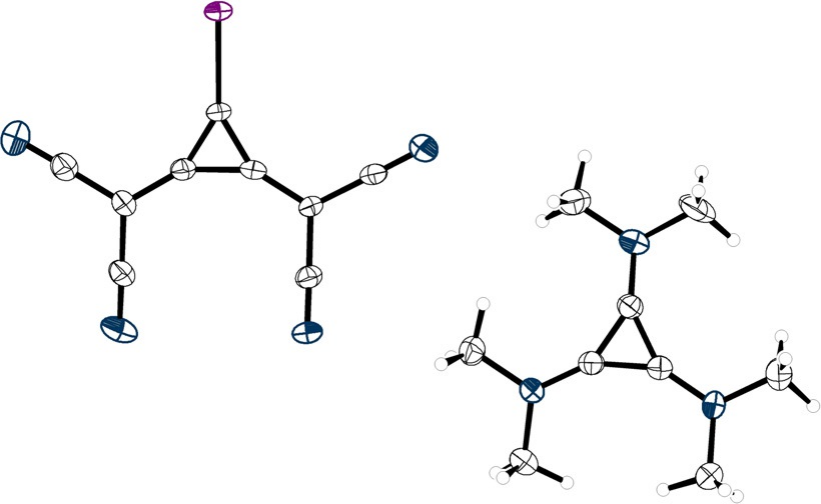
X‐ray structural analysis of **1**. Thermal ellipsoids at 50 % probability level.

Aiming for a more efficient synthesis, we reacted **2** with sodium borohydride to form the desired anion of **3** as a water‐soluble sodium salt. Cation exchange from sodium to the organic cation tris(dimethylamino)cyclopropenium (TDA)^38^ increases the solubility of the anion in organic solvents, and **3** was obtained in 63 % yield. Iodination of the latter was performed with 1,3‐diiodo‐5,5‐dimethylhydantoin (**4**) in DCM over two hours.^39^ The product **1**, which was isolated in 81 % yield, was crystallized from either DCM/diethyl ether or DCM/cyclopentane. Single‐crystal analysis^40^ confirmed the formation of the desired XB donor (Figure 2).

**Figure 3:**
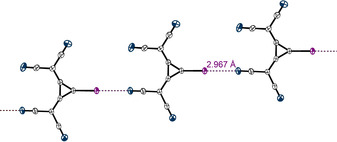
Cutout of the XB‐based planar chains formed by 1,2‐bis(dicyanomethylene)‐3‐iodo‐cyclopropanid anions in the crystal structure of **1**. The TDA cations, which are positioned alongside the depicted chain, are omitted. Thermal ellipsoids at 50 % probability level.

A closer inspection of the crystal structure revealed short contacts between the iodine substituents of one molecule and the nitrogen atoms of a second molecule (Figure 3), a very pronounced case of AEXB between two organic molecules in the solid state. The interaction features a bond length of *d*_I‐N_=2.97 Å (16 % shorter than the sum of the vdW radii (3.53 Å),^34^ R_XB_=0.84^41^) and shows the characteristic high directionality of XBs with angles of ∡_C‐I⋅⋅⋅N_=178.8°. The AEXBs result in infinite chains of **1**, and the counterions are positioned alongside these chains (see Figures S29–S31).

Inspired by these results, we wondered whether the anionic bis(dicyanomethylene)cyclopropanid substituent (L^−^) could be incorporated into other systems to obtain further AEXBs and 2‐iodoimidazole (**5**) seemed to be a promising core structure for this purpose. Therefore, calculations similar to the ones mentioned above were performed on the imidazolyl‐substituted anion **6** (Scheme 1). In this case, the negative charge is also distributed over the entire molecule and the region of least negative electrostatic potential (σ‐hole) on iodine is even more negative (−127.9 kJ mol^−1^) than in **1**. Scans of the C−I⋅⋅⋅X^−^ (X=Cl, I) distances yielded similar energy profiles as obtained for **1**⋅⋅⋅X^−^ (compare Figure 1). While the gas‐phase data of the complex **6**⋅⋅⋅Cl^−^ is mostly in line with the results of **1**⋅⋅⋅X^−^ (Table 1, entry 5), a less stable adduct was found for adduct **6**⋅⋅⋅I^−^ (entry 6), which is evident by the comparably small well depth (−3.0 kJ mol^−1^) and the deviation from linearity (157°). In acetonitrile, both adducts showed similar well depths and geometric parameters (entries 7 and 8) as **1**⋅⋅⋅X^−^ (entries 3 and 4), with linear arrangements and higher kinetic stability (well depths of −18 to −21 kJ mol^−1^). In contrast to the complexes with **1**, however, the binding energies and Gibb's free energies are predicted to be positive.

The synthesis of imidazolyl derivative **6** was achieved in a straight‐forward manner by deprotonation of 2‐iodoimidazole (**5**) with lithium diisopropylamide (LDA) and subsequent reaction with inner salt **2**. To obtain a soluble compound, organic counterions were then introduced leading to the TDA salt **6 a** and tetrabutylammonium (NBu_4_) salt **6 b** in 62 and 66 % overall yield, respectively. Single crystals for both **6 a** and **6 b** could be grown in DCM/diethyl ether and X‐ray structural analyses revealed that in both cases, short contacts (XBs) between iodine substituents and nitrogen atoms either from cyano groups or the imidazole are found. However, depending on the counterion, two different patterns are observed.

**Figure 4:**
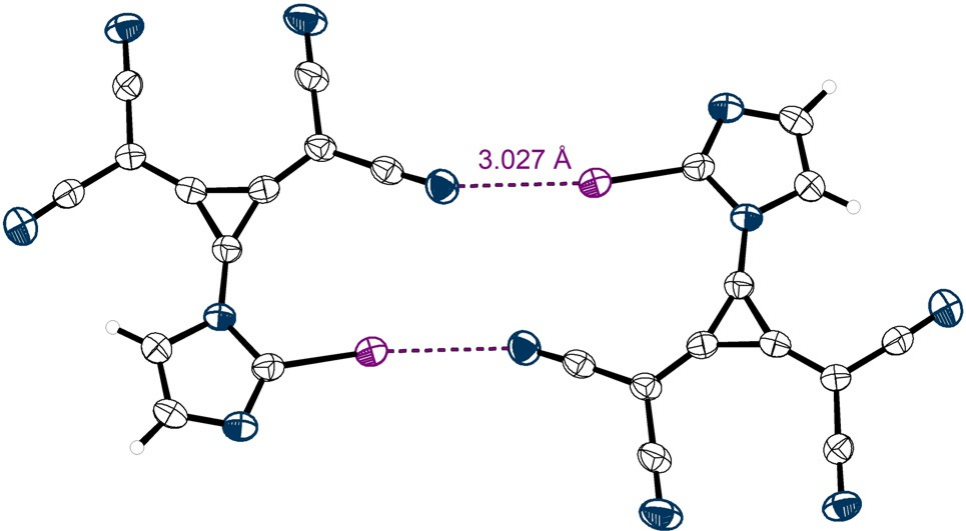
The dimers **6**⋅⋅⋅**6**_2XB_ found in the X‐ray structural analysis of **6 b** (see Scheme 1). Tetrabutylammonium counterions and a DCM molecule, which is incorporated in the crystal structure but does not interact with the XB donor, are omitted for clarity. Thermal ellipsoids at 50 % probability level.

Figure 4 shows the symmetric dimers **6**⋅⋅⋅**6**_2XB_ which exist in the crystal structure of the tetrabutylammonium salt **6 b**. The halogen bonds feature a longer interaction distance (*d*_I‐N_=3.03 Å, R_XB_=0.86) and a less linear angle (∡_C‐I‐N_=172.76°) compared to the AEXBs observed with **1** (Figure 3). The imidazole and cyclopropanid rings in one molecule are not coplanar but are slightly twisted by about 15°. In addition to the strong XBs, weak HBs to the counterions are detected [*d*_C‐H_=2.78 and 2.72 Å (R_CH_=0.97–0.99) and *d*_N‐H_=2.45–2.62 Å (R_HB_=0.92–0.99)], so that each dimer is surrounded by eight tetrabutylammonium ions (see Figure S28).

**Figure 5:**
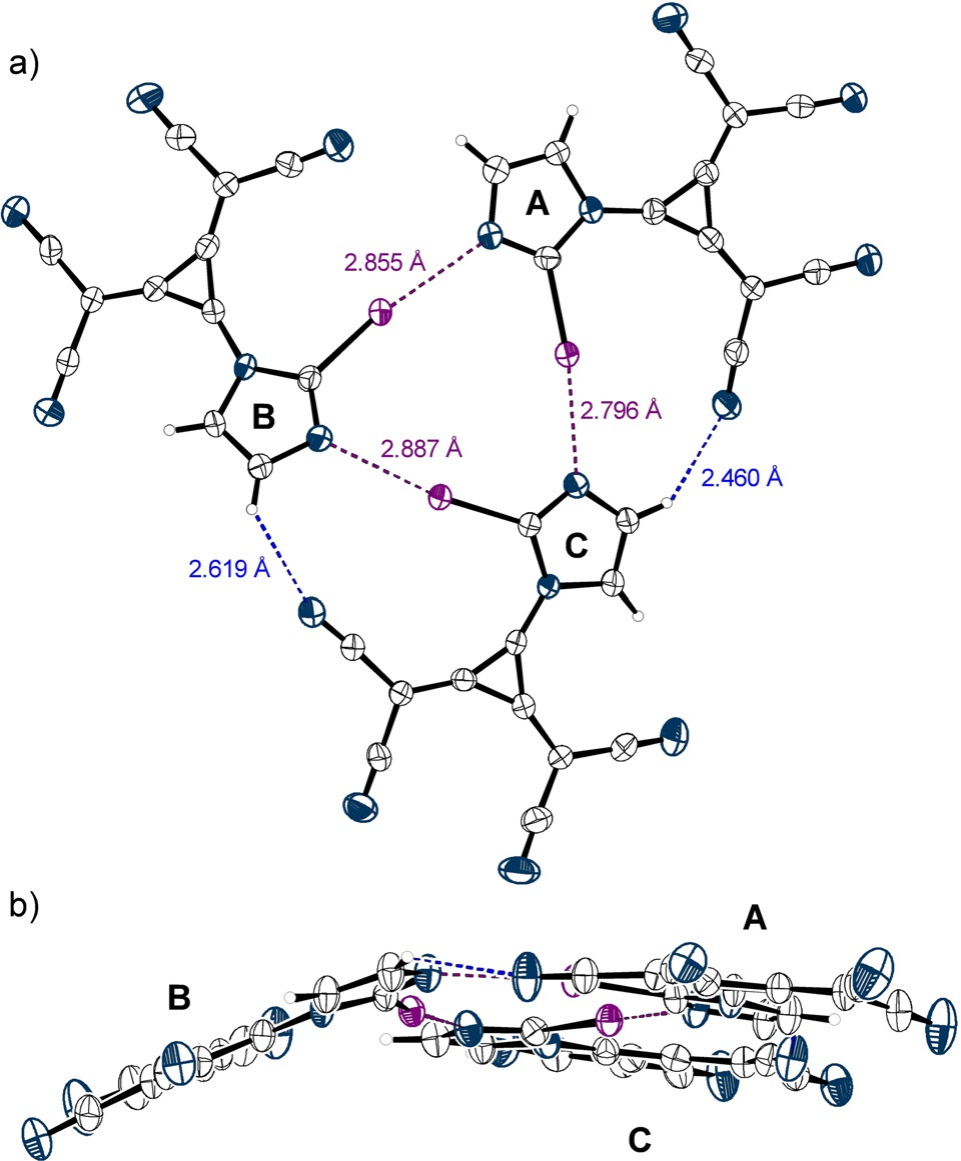
Segment of the X‐ray structural analysis of **6 a** (see Scheme 1). Counterions and an additional XB donor molecule, which is positioned at the center of symmetry and is not involved in any XB, are omitted (see Figures S32 and S41). Thermal ellipsoids at 50 % probability level. a) Top view on the trimeric pattern. Halogen bonds are marked in purple [∡_C‐I‐N_=173° (A⋅⋅⋅C), 170° (B⋅⋅⋅A) and 172° (C⋅⋅⋅B)]. Hydrogen bonds are indicated in blue. The third H‐N‐distance of 2.65 Å corresponds exactly to the sum of vdW radii (2.65 Å). Dihedral angles are 4.29° for A, 10.78° for B, and −12.54° for C. b) Side‐view of the trimeric pattern.

In case of the TDA salt **6 a**, a trimeric motif is observed, in which the iodine substituent of each XB donor interacts with the imidazole nitrogen atom of another one (Figure 5 a). This triangular motif was previously reported for other XB donor systems by Metrangolo et al.^42^ and Mukai and Nishikawa^43^ as well as for gold carbene complexes.^44^ In the trimer of **6 a** the XB bond distances (*d*_I‐N_=2.80–2.89 Å, R_XB_=0.79–0.81) are the shortest ones found in all self‐associated crystal structures described herein. Two of the three XB donors in the trimeric complex lie in the same plane (A and C), whereas the third molecule (B) is tilted out of this plane by about 20° (Figure 5 b). In addition to various short contacts of the depicted molecules to counterions and adjacent iodoimidazolyl species (see Figure S32), the XB complex is further stabilized by two weak HBs (R_HB_=0.88 and 0.94, Figure 5 a).

All these motifs found in the crystal structure not only demonstrate the feasibility of forming XBs between ions of like charge, but they also exhibit cooperativity, which has been discussed for XBs in the literature both theoretically^45^ and experimentally.^46^ This cooperativity can probably best be explained from the n→σ* orbital interaction point of view: donation of electron density into the σ* orbital of an XB donor will render its Lewis basic centers (in this case, nitrogen atoms of cyano or imidazole groups) more electron‐rich and will thus reinforce their interaction with a second XB‐donating moiety. In agreement with the solid‐state data, these polarization‐assisted XBs (PAXBs) are expected to be more pronounced for the trimer (Figure 5) compared to the dimer (Figure 4), as the relevant Lewis basic centres are much closer to the C−I bond in the former case.

The motifs found in the crystal structures were used for further DFT calculations. For the complexes involving **6**, similar structures to those of the solid‐state geometries were obtained (see also Table 1, entries 9 and 10; **6**⋅⋅⋅**6**_1XB_ designates a complex with a single XB). While the structure of a dimer of **1** in the gas phase deviated significantly from the geometries found in the chainlike motif (Figure 3), calculations in acetonitrile resulted in a linear arrangement of both XB donors and reasonable kinetic stability (Table 1, entry 12). NCI‐plots, visualising noncovalent interactions,^47^ of all motifs showed only attractive interactions between the molecules (see Figure S48).

**Figure 6:**
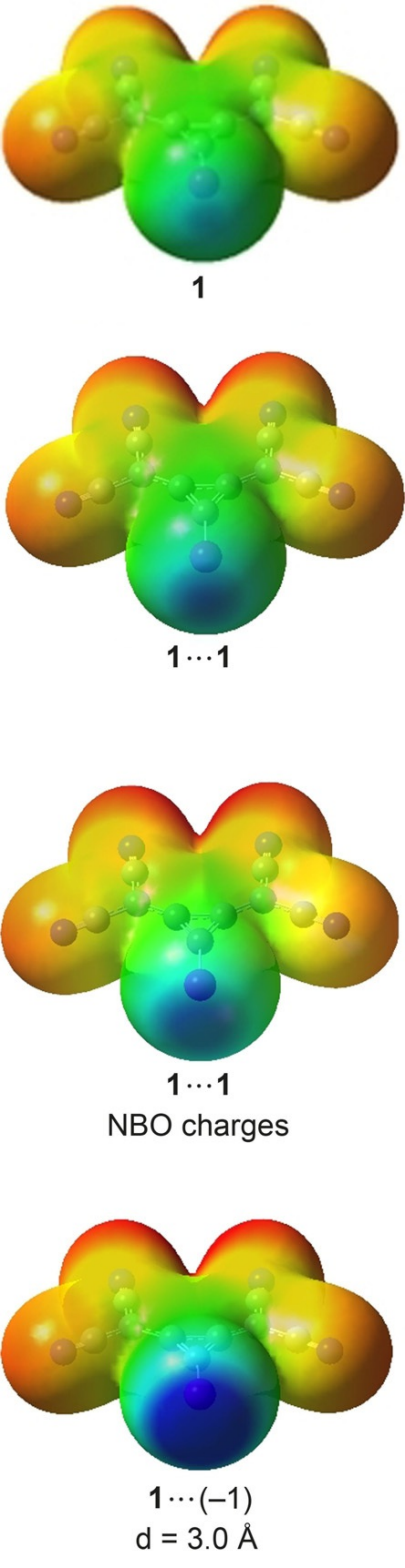
Electrostatic potential on the 0.001 au isosurface with and without point charges. The scale ranges from −417 to 0 kJ mol^−1^.

To further investigate the influence of the binding partner on the electrostatic potential of the XB donors, calculations with point charges positioned in elongation of the C−I bond were performed.^48^ Single‐point charges varying from +1 to −1 at XB‐relevant distances vividly illustrated the high polarizability of **1**, with *V*_S,max_ values between −266.1 and 154.7 kJ mol^−1^ (Table 2). To better simulate a second XB donor like **1** or **6**, we also placed the atomic charges (NBO^49^ or Mulliken^50^ charges) of all the atoms of a binding partner at their respective atomic position in space (to mimic **1**⋅⋅⋅**1** or singly/doubly bound **6**⋅⋅⋅**6** complexes). As expected, their influence on the electrostatic potential of the investigated XB donor is less pronounced than for single‐point charges, and in almost all cases *V*_S,max_ remained negative (Figure 6 and Table 2). Thus, while polarization can significantly decrease the repulsion between the XB donors,^51^ this in itself does not seem sufficient to explain XB self‐association.

**Table 2:** Electrostatic potential on the 0.001 electron/Bohr^3^ isosurface of the electronic density. The distance between point charge and donor is given in Å and the σ‐hole energies (*V*_S,max_) in kJ mol^−1^.

	Charge	*d*	*V*_S,max_
**1**	–	–	−80
**1**	+1.0	3.5	−266
**1**	−0.5	3.5	5.7
**1**	−1.0	3.5	92
**1**	−1.0	3.0	155
**1**	**1**_nbo_	–^[a]^	−16
**1**	**1**_Mulliken_	‐^[a]^	−9.6
**6**	–	–	−128
**6**	**6**_nbo_ (1XB)	–^[b]^	−17
**6**	**6**_Mulliken_ (1XB)	–^[b]^	2.9
**6**	**6**_nbo_ (2XB)	–^[c]^	−31
**6**	**6**_Mulliken_ (2XB)	–^[c]^	−45

[a] The optimized structure of the calculation in acetonitrile was used. [b] A dimer with both XB donors positioned in one plane featuring one XB contact [see Figure 5 (**A**⋅⋅⋅**C**)] was used as a template. [c] The dimer structure shown in Figure 4 was used as a template.

Finally, co‐crystals of **1** with halides were targeted as even more drastic examples of AEXBs: because of their localized negative charge, they seem to be more challenging substrates to coordinate to anionic XB donors. In contrast, DFT calculations in polar environments had demonstrated the feasibility of adduct formation (Figure 1) and the calculations discussed in the last paragraph indicated that halides would also induce a strong favourable polarization of the XB donor.

**Figure 7:**
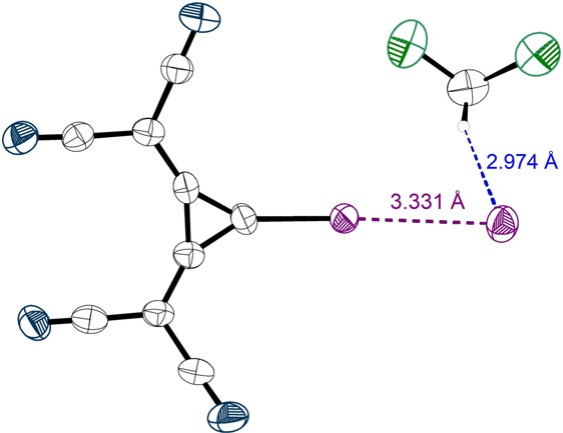
X‐ray structural analysis of a 1:1 co‐crystal of **1** with iodide. ∡_C‐I‐I_=177.5° The TDA counterions are omitted. Thermal ellipsoids at 50 % probability level.

**Figure 8:**
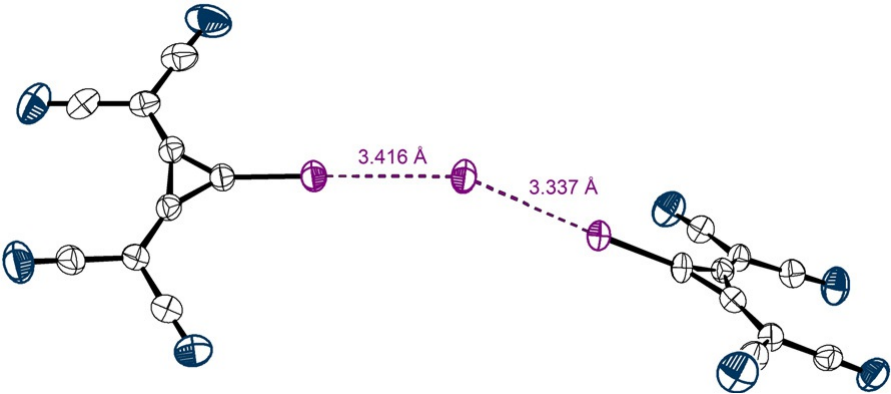
X‐ray structural analysis of a 1:2 co‐crystal of **1** with iodide. ∡_C‐I‐I_=172 and 178°, ∡_I‐I‐I_=152°. TDA counter‐ions and one DCM molecule, interacting weekly with iodide (see the Supporting Information), are omitted. Thermal ellipsoids at 50 % probability level.

Therefore, TDAX (X=I, Br, Cl) salts were added in different ratios (1:1, 1:2, and 1:3) to a solution of the XB donor **1** in DCM. As cosolvents cyclopentane and diethyl ether were used, resulting in the formation of several single crystals suitable for XRD measurements. Figure 7 and Figure 8 show the 1:1 and 1:2 co‐crystal, respectively, of TDAI with **1**.^52^ The crystal structure of the 1:1 complex shows one XB contact with an I—I distance of 3.33 Å, R_XB_=0.84 (R_XB_^ion^=0.80 if anion radii according to Pauli^53^ are considered for iodide). Additionally, one solvent molecule is incorporated in the crystal structure, which is weakly bound to iodide.

Such a weak hydrogen bond is also observed between DCM and iodide in the crystal structure of the 1:2 complex, in which two XB donor molecules coordinate to one iodide (Figure 8). One of these interactions shows almost the same geometric parameters (∡_C‐I‐I_=178°; d_I‐I_=3.33 Å) as found in the 1:1 complex. The second contact is slightly longer (*d*=3.41 Å) and a bit less linear (∡_C‐I‐I_=172°). Nevertheless, both interactions clearly constitute XBs, and thus this structure represents an XB adduct between three anions! The planes of the three‐membered rings are in an angle of 45° to one another and the angle between the two XBs is ∡_I‐I‐I_=152°. This motif could also be considered, to some degree, as an umpoled version of triiodide (I_3_^−^; which may be described as I^−^⋅⋅⋅I^+^⋅⋅⋅I^−^, ∡_I‐I‐I_=180°). A more fitting comparison, however, is pentaiodide, which may be described as a central iodide coordinated to two terminal I_2_ units (∡_I2‐I‐I2_=95°)^54^—the latter being replaced by anionic XB donors in our case.

**Figure 9:**
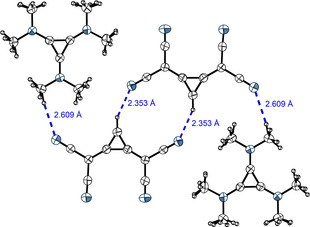
In the crystal structure of **3**, symmetric hydrogen‐bonded dimers are found (R_HB_=0.89). Thermal ellipsoids at 50 % probability level.

Incidentally, during the course of our studies, we also obtained a single crystal of **3** (by vapor diffusion using DCM/cyclopentane) and the corresponding X‐ray structural analysis exhibited AEHBs (Figure 9). The compound forms dimers in which the hydrogen substituent of one molecule interacts with the cyano substituent of a second molecule by AEHBs (R_HB_=0.89 and ∡_C−H⋅⋅⋅LB_=152.9°). The dimer motif is complemented by two TDA counterions, which are weakly coordinated to other cyano groups of the anions, thus forming a planar motif which seems to interact only by dispersion forces with other such assemblies. In case of the corresponding tetrabutylammonium salt, no AEHB is found, which indicates that AEHB formation is favored by the planar arrangement in the solid‐state structure of **3**.

**Figure 10:**
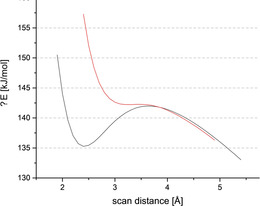
Energy profile of the scan of the H⋅⋅⋅X^−^ bond distance for the adduct of **3** with chloride (black) and iodide (red) in the gas phase.

To confirm the ability of **3** to stabilize AEHBs, DFT calculations, similar to the ones mentioned above for the I analogues, were performed. The obtained energy profiles deviate strongly from the ones found for the corresponding AEXBs: in the gas‐phase calculations, there is comparably little kinetic stabilization of the complexes (Table 1, entries 13 and 14 and Figure 10), markedly less than for the XB variants. The calculations with intrinsic solvation model, in contrast, yield energy curves with much more pronounced wells and predict that the adducts should be overall strongly exergonic, again in sharp contrast to the AEXB versions (entries 15 and 16; see Figures S55 and S56). The latter results should be taken cum grano salis, however, as it is unclear how well this computational model can reflect the actual situation in solution.

## Conclusion

In summary, two anionic organic XB donors based on 1,2‐bis(dicyanomethylene)cyclopropanid moieties were synthesized and characterized by solid‐state structures, which featured multiple forms of XB‐based self‐association of the anionic molecules. Even more pronounced (and previously unprecedented) examples of such “anti‐electrostatic” XBs were obtained in the form of halide adducts. DFT calculations supported the feasibility of adduct formation in polar environments and illustrated the polarization of the XB donors by the interaction partner (but still predicted entirely negative electrostatic potentials for the self‐associated adducts). In our view, all these examples highlight the importance of polarization and n→σ* orbital interactions for halogen bonding and demonstrate once again^7^, ^55^ that a description of this interaction by static σ‐holes is doomed to fail. These findings may also pave the way towards the utilization of novel classes of (anionic) XB donors, which were so far almost exclusively based on neutral or cationic compounds.

## Conflict of interest

The authors declare no conflict of interest.

## Supporting information

As a service to our authors and readers, this journal provides supporting information supplied by the authors. Such materials are peer reviewed and may be re‐organized for online delivery, but are not copy‐edited or typeset. Technical support issues arising from supporting information (other than missing files) should be addressed to the authors.

Supplementary
