# Supplementary material for: “Anti‐Electrostatic” Halogen Bonding
Source: Angew Chem Int Ed Engl. 2020 Apr 30;59(27):11150–7. doi: 10.1002/anie.202003083 (PMC7317790; doi:10.1002/anie.202003083)
Supplement: Supplementary file 1 — Supplementary [file ANIE-59-11150-s001.pdf]

Angewandte Chemie
Eine Zeitschrift der Gesellschaft Deutscher Chemiker

Supporting Information

“Anti-Electrostatic” Halogen Bonding**

Jana M. Holthoff, Elric Engelage, Robert Weiss, and Stefan M. Huber**

anie_202003083_sm_miscellaneous_information.pdf

Table of Contents

I. Experimental Procedures

II. NMR- and IR-Spectra

III. X-ray Structures

IV. Computational Details

V. References

I. Experimental Procedures

A) General Information

A.1) Chemicals

Chemicals were obtained from *ABCR, Acros, Alfa Aesar, Carbolution Chemicals, Chempur, Fisher Scientific* or *Sigma-Aldrich*. Deuterated solvents were obtained from *Deutero* and *Eurisotop* and stored over molecular sieves (3 Å). Commercially available reagents and starting materials were, unless mentioned otherwise, used without further purification. Dichloromethane (DCM), tetrahydro furan (THF) and diethyl ether were obtained in technical grade quality and distilled before drying with a solvent drying system by *M. Braun* (type: MB SPS-800). Acetonitrile was dried over molecular sieves (3 Å). All dried solvents or reagents were stored over molecular sieves under an argon atmosphere and their water content was monitored via Karl-Fischer titration (*SI Analytics* (*Xylem*) Titroline 7500 KF Trace Karl Fischer system using *Honeywell* Hydranal Coulomat-AD. For column chromatography silica gel (grain size: 0.04-0.63 cm, *Merck*, Si_{60}) or aluminium oxide (grain size: 0.063-0.200 mm, *Merck*, aluminium oxide 90 active neutral) were used. Thin layer chromatography (TLC) was performed on plates from *Merck* (silica gel 60, F254) or on aluminium oxide plates from *Macherey-Nagel* (ALUGRAM ALOX N/UV_{254}).
All reactions were performed without the exclusion of air or moisture or carried out under standard Schlenk conditions using high-vacuum/oven-dried glassware, proceeding under argon atmosphere, when reagents or products are sensitive to air and moisture. Reagents were injected via a septum or added under argon counter-flow.

A.2) Appliances and Materials

NMR-spectra were obtained on instruments of the type DRX 400, AVIII 400, AVIII 300 and Advanced Neo 500 (equipped with a three channel cryo probe TXO (CRPHe TR-^{13}C/^{15}N/^{1}H 5mm-Z)) from *Bruker*. Coupling constants (*J*) are given in hertz [Hz] and chemical shifts (δ) in parts per million [ppm]. Multiplicities are assigned as s (singlet), d (doublet), t (triplet), q (quartet) and sept (septet). Mass spectra (MS-spectra) were obtained by using electron ionization (EI) on Jeol AccuTOF GCy or electron spray ionization (ESI) on an esquire6000 instrument from *Bruker*. Elemental analysis was performed on vario Micro cube (*Elementar Analysensysteme*). For IR-spectroscopy a *Shimadzu IRAffinity*-1S and *Specac* ATR were used. The relative intensities of the IR-signals are denoted as very weak (vw), weak (w), medium (m), strong (s) and very strong (vs). Broad absorption bands are labelled as such (bs).

B) Syntheses

B.1) 1,2-Bis(dicyanomethylene)-3-triethylammonium-cyclopropanid 2

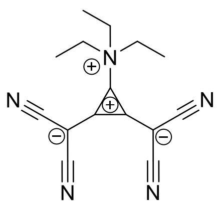

Chemical Formula: $C_{15}H_{15}N_5$
Molecular Weight: 265,32

The compound was synthesised following the procedures described in literature.[1]

To a solution of 6.85 g (38.52 mmol, 1.00eq.) tetrachlorocyclopropene and 5.47 g (82.82 mmol, 2.15 eq.) malononitrile in 330 ml dry DCM at -30 to -40 °C triethylamine (30.0 ml, 216.48 mmol, 5.62 eq.) in 20 ml DCM was added dropwise under an argon atmosphere. Upon addition the solution turned yellow and a solid crushed out. When the addition of the base was completed, the reaction mixture was slowly warmed to 0 °C and water was added to dissolve NEt_3•HCl. The product was filtered off and washed rigorously with water, methanol and with DCM. The product **2** was obtained as a beige solid in 79% yield (8.08 g. 30.45 mmol) and was used without further purification.

^{1}H-NMR (acetonitrile-d_3, 400 MHz, 300 K): δ = 4.33 (q, *J* = 7.2 Hz, 6H, C$\underline{H}_2$CH$_3$), 1.94 (t, *J* = 7.2 Hz, 9H, CH$_2$C$\underline{H}_3$) ppm.

^{1}H-NMR (DMSO-d_6, 300 MHz,298 K): δ = 3.82 (q, *J* = 7.2 Hz, 6H, C$\underline{H}_2$CH$_3$), 1.30 (t, *J* = 7.2 Hz, 9H, CH$_2$C$\underline{H}_3$) ppm.

^{1}H-NMR (acetone-d_6, 300 MHz, 298 K): δ = 4.08 (q, *J* = 7.2 Hz, 6H, C$\underline{H}_2$CH$_3$), 1.56 (t, *J* = 7.2 Hz, 9H, CH$_2$C$\underline{H}_3$) ppm.

^{13}C-NMR (acetonitrile-d_3, 100 MHz, 300 K): δ = 135.44 (C^1 and C^2), 116.31 (C($\underline{C}$N)$_2$), 115.51 (C($\underline{C}$N)$_2$), 112.08 (C^3), 58.32 ($\underline{C}$H$_2$CH$_3$), 35.41 ($\underline{C}$(CN)$_2$), 7.66 (CH$_2\underline{C}$H$_3$) ppm.

Elemental Analysis: calc.: C: 67.90, H: 5.70, N: 26.40
found: C: 66.79, H: 5.433, N: 26.39.

MS (EI, 70 eV): *m/z* (%) = 265.1 (58) [M]$^+$, 237.1 (64) [M-CH$_2$CH$_3$]$^+$, 208.1 (55) [M-2CH$_2$CH$_3$]$^+$, 180.0 (32) [M-3CH$_2$CH$_3$]$^+$, 101.1 (8) [NEt$_3$]$^+$, 86.1 (51) [NEt$_2$CH$_2$]$^+$, 29.0 (100) [CH$_2$CH$_3$]$^+$.

IR: signal (intensity) = 2987 (w) [C-H], 2212 (vs) [CN], 2197 (vs) [CN], 1886 (s) [cyclopropenyl], 1516 (vs), 1474 (s), 1464 (s), 1447 (s), 1383 (s), 1358 (s), 1319 (s), 1196 (m), 1167 (m), 1142 (m), 1094 (m), 1074 (w), 1059 (m), 1036 (w), 1011 (m), 993 (s), 935 (w),901 (m), 878 (w), 818 (m), 785 (s), 665 (m), 596 (s), 555 (m), 545 (m), 498 (w), 459 (w), 434 (w), 413 (w) cm^{-1}.

B.2) Tris(dimethylamino)cyclopropenylium 1,2-bis(dicyanomethylene)-3-hydro-cyclopropanid 3

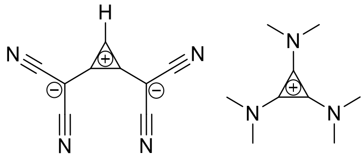

Chemical Formula: $C_{18}H_{19}N_7$
Molecular Weight: 333,40

2.60 g (9.80 mmol, 1.00 eq.) 1,2-bis(dicyanomethylene)-3-triethylammonium-cyclopropanid **2** were suspended in 500 ml methanol and cooled to 0 °C. 389 mg (10.29 mmol, 1.05 eq.) sodium borohydride were added portion-wise. Upon addition, gas evolution was observed. When the addition was completed, the ice bath was removed, and the reaction mixture was stirred for 2 h at room temperature. Afterwards, the solvent was removed under reduced pressure and the residue was dissolved in 60 ml water. The product crushed out of the solution, when tris(dimethylamino)cyclopropenylium hydrate **3** (TDACl·H$_2$O) (2.17 g, 9.80 mmol, 1.00 eq.) dissolved in 7 ml water were added to the aqueous solution of the sodium salt. The precipitate was filtered off, washed with 10 ml water and dissolved in 20 ml DCM. The organic phase was dried over magnesium sulfate and filtered. The solution of the crude product in DCM was filtered slowly(!) over activated carbon. Afterwards the product was precipitated with diethyl ether. The product was obtained in 58% yield (1.91 g, 5.73 mmol) as a white solid.

^{1}H-NMR (chloroform-*d*, 400 MHz, 300 K): δ = 7.34 (s, 1H, $C^3\underline{H}$), 3.13 (s, 18H, TDA-$C\underline{H}_3$) ppm.

^{1}H-NMR (acetonitrile-d_3, 300 MHz, 298 K): δ = 7.60 (s, 1H, $C^3\underline{H}$), 3.07 (s, 18H, TDA-$C\underline{H}_3$) ppm.

^{13}C-NMR (chloroform-*d*, 100 MHz, 300 K): δ = 141.71 (C^1 and C^2), 118.90 (*C*N), 118.47 (*C*N), 117.59 (TDA), 107.64 (C^3H), 42.51 (TDA-CH_3), 33.89 ($C(\underline{C}N)_2$) ppm.

^{13}C-NMR (chloroform-*d*, 100 MHz, 300 K): δ = 141.71 (d, *J* = 1.1 Hz, C^1 and C^2), 118.90 (d, *J* = 1.4 Hz, $(C(\underline{C}N)_2)$, 118.47 $(C(\underline{C}N)_2)$, 117.58 (dt, *J* = 7.9, 3.9 Hz, TDA-C_3), 107.64 (d, *J* = 239.2 Hz, $\underline{C}^3$H), 42.50 (qq, *J* = 139.3, 3.2 Hz, TDA-$\underline{C}H_3$), 33.89 ($\underline{C}(CN)_2$) ppm.

Elemental Analysis: calc.: C: 64.85, H:5.74, N:29.41
found: C: 64.45, H: 5.706, N: 29.52.

MS (ESI): positive mode: *m/z* = 167.99, negative mode: *m/z* = 164.77.

IR: signal (intensity) = 3578 (m) [H_2O], 3115 (s) [C^3H], 2932 (m) [C-H], 2884 (m) [C-H], 2849 (w), 2801 (w) 2210 (w), 2201 (vs) [CN], 2183 (vs) [CN], 2145 (w), 1821 (s) [cyclopropenyl], 1549 (vs), 1439 (s), 1402 (vs), 1277 (m), 1217 (s), 1157 (s), 1092 (w), 1055 (w), 1026 (s), 974 (vw), 874 (w), 783 (s), 766 (s), 696 (w), 611 (m), 573 (s), 550 (w), 496 (m), 455 (m) cm^{-1}.

B.3) Tris(dimethylamino)cyclopropenylium 1,2-bis(dicyanomethylene)-3-hydro-cyclopropanid 3•NBu$_4$

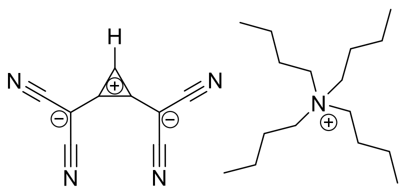

Chemical Formula: $C_{25}H_{37}N_5$
Molecular Weight: 407,61

1.00 g (3.77 mmol, 1.00 eq.) 1,2-bis(dicyanomethylene)-3-triethylammonium-cyclopropanid **2** was suspended in 300 ml methanol and cooled to 0 °C. 150 mg (3.96 mmol, 1.05 eq.) sodium borohydride were added in portions. Upon addition, gas evolution was observed. When the addition was completed, the ice bath was removed, and the reaction mixture was stirred for 1.5 h at room temperature. Afterwards, the solvent was removed under reduced pressure and the residue was dissolved in 60 ml water. The product crushed out of the solution, when tetrabutylammonium chloride (1.05 g, 3.77 mmol, 1.00 eq.), dissolved in 5 ml water, was added to the aqueous solution of the sodium salt. The precipitate was filtered off, washed with 10 ml water and dissolved in 15 ml DCM. The organic phase was dried over magnesium sulfate and filtered. Column chromatography using DCM:MeOH (20:1) yielded 1.09 g (2.67 mmol, 71%) tetrabutylammonium 1,2-bis(dicyanomethylene)-3-hydro-cyclopropanid **3•NBu$_4$** as colourless to beige powder. When the product still shows brown impurities, the product can be reprecipitated using DCM/diethyl ether.

^{1}H-NMR (DMSO-d_6, 300 MHz, 300 K): δ = 8.44 (s, 1H, C^3*H*), 3.24 – 3.02 (m, 8H, $N(C\underline{H}_2CH_2CH_2CH_3)_4$), 1.57 (p, *J* = 7.9 Hz, 8H, $N(CH_2C\underline{H}_2CH_2CH_3)_4$), 1.31 (h, *J* = 7.3 Hz, 8H, $N(CH_2CH_2C\underline{H}_2CH_3)_4$), 0.94 (t, *J* = 7.3 Hz, 12H, $N(CH_2CH_2CH_2C\underline{H}_3)_4$) ppm.

^{1}H-NMR (chloroform-*d*, 300 MHz, 300 K): δ = 7.32 (s, 1H, C^3*H*), 3.21 – 3.06 (m, 8H, $N(C\underline{H}_2CH_2CH_2CH_3)_4$), 1.70 – 1.51 (m, 8H, $N(CH_2C\underline{H}_2CH_2CH_3)_4$), 1.40 (h, *J* = 7.3 Hz, 8H, $N(CH_2CH_2C\underline{H}_2CH_3)_4$), 0.99 (t, *J* = 7.3 Hz, 12H, $N(CH_2CH_2CH_2C\underline{H}_3)_4$) ppm.

^{1}H-NMR (acetonitrile-d_3, 300 MHz, 295 K): δ = 7.60 (s, 1H), 3.15 – 3.01 (m, 8H, $N(C\underline{H}_2CH_2CH_2CH_3)_4$), 1.59 (ddt, *J* = 11.7, 8.2, 3.9 Hz, 8H, $N(CH_2C\underline{H}_2CH_2CH_3)_4$), 1.35 (dq, *J* = 14.4, 7.3 Hz, 8H, $NCH_2CH_2C\underline{H}_2CH_3)_4$), 0.97 (t, *J* = 7.3 Hz, 12H, $N(CH_2CH_2CH_2C\underline{H}_3)_4$) ppm.

^{13}C-NMR (chloroform-*d*, 75 MHz, 300 K): δ = 141.93 (C^1 and C^2), 119.12 $(C(\underline{C}N)_2)$, 118.83 $(C(\underline{C}N)_2)$, 108.11 (C^3), 58.88 (t, *J* = 2.4 Hz, $N(\underline{C}H_2CH_2CH_2CH_3)_4$), 33.92 ($\underline{C}(CN)_2$), 23.87 ($N(CH_2\underline{C}H_2CH_2CH_3)_4$), 19.73 ($N(CH_2CH_2\underline{C}H_2CH_3)_4$), 13.61 ($N(CH_2CH_2CH_2\underline{C}H_3)_4$) ppm.

Elemental Analysis: calc.: C: 73.67, H: 9.15, N: 17.18
found: C: 73.17, H: 8.810, N: 17.45.

MS (ESI): positive mode: *m/z* = 242.15, negative mode: *m/z* = 164.80.

IR: signal (intensity) = 3138 (m) [C^3H], 2961 (s) [C-H], 2932 (m) [C-H], 2872 (m) [C-H], 2205 (vs) [CN], 2189 (vs) [vs], 1817 (vs) [cyclopropyl], 1734 (vw), 1493 (m), 1450 (vs), 1383 (s), 1310 (vw), 1269 (m), 1217 (m), 1153 (s), 1107 (w), 1092 (w), 1055 (w), 1026 (w), 968 (w), 881 (s), 806 (m), 741 (m), 718 (s), 610 (w), 571 (s), 519 (m), 496 (m) cm^{-1}.

R_f = 0.6, (DCM:MeOH = 20:1)

B.3) Tris(dimethylamino)cyclopropenylium 1,2-bis(dicyanomethylene)-3-iodo-cyclopropanid 1

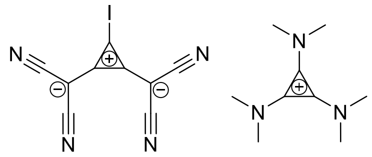

Chemical Formula: $C_{18}H_{18}IN_7$
Molecular Weight: 459,30

200 mg (0.60 mmol, 1.00 eq.) tris(dimethylamino)cyclopropenylium 1,2-bis(dicyanomethylene)-3-hydro-cyclopropanid **3** were dissolved in 10 ml dichloromethane and the iodination agent 1,2-diiodo-5,5-dimethylimidazolidine-2,4-dione **4** (137 mg, 0.36 mmol, 0.6 eq.) was added. The reaction mixture was stirred for 2.5 h. Afterwards, the dark green to brown solution was washed with water thrice. The organic phase was dried over magnesium sulfate and filtered. The solvent was removed under reduced pressure without applying heat. To remove impurities 3 ml methanol were added to the residue. The insoluble solid was filtered off and washed with an additional 2 ml of methanol and with diethyl ether, before drying the yellow to ochre powder. Tris(dimethylamino)cyclopropenylium 1,2-bis(dicyanomethylene)-3-iodo-cyclo-propanid **1** was obtained in 70% yield (192 mg, 0.42 mmol).

^{1}H-NMR (dichloromethane-d_2, 300 MHz, 295 K): δ = 3.14 (s, 18H, TDA-C$\underline{H}_3$) ppm.

^{13}C-NMR (chloroform-*d*, 75 MHz, 300 K): δ = 146.93 (C^1 and C^2), 118.39, 118.03 (TDA-C_3), 117.54, 42.96 (TDA-$\underline{C}H_3$), 34.86 ($\underline{C}$(CN)$_2$) ppm.

Elemental Analysis: calc.: C: 47.07, H: 3.95, N: 21.35
found: C: 46.74 H: 3.988, N: 21.45.

MS (ESI): positive mode: *m/z* = 168.01, negative mode: product anion was not detected.

IR: signal (intensity) = 2967 (w) [C-H], 2930 (m) [C-H], 2887 (w) [C-H], 2845 (vw), 2442 (vw), 2345 (vw), 2309 (vw), 2210 (s) [CN], 2201 (vs) [CN], 2187 (vs) [CN], 2143 (m), 1838 (s) [cycloporpoenyl], 1555 (vs), 1449 (vs), 1420 (s), 1396 (vs), 1306 (m), 1294 (s), 1219 (s), 1138 (s), 1107 (w), 1082 (m), 1055 (m), 1028 (s), 937 (w), 891 (vw), 785 (s), 735 (w), 700 (w), 625 (vw), 610 (w), 579 (s), 662 (m), 498 (m), 459 (m), 428 (w),
419 (w) cm^{-1}.

B.4) Tris(dimethylamino)cyclopropenylium 1,2-bis(dicyanomethylene)-3-(2-iodoimidazolyl)-cyclopropanid 6a

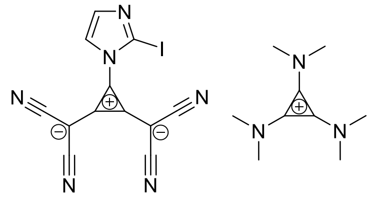

Chemical Formula: $C_{21}H_{20}IN_9$
Molecular Weight: 525,36

Under an argon atmosphere 146 mg (0.75 mmol, 1.00 eq.) 2-iodominidazole **5** were dissolved in 15 ml dry THF and cooled to -78 °C. A cooled solution of 0.75 mmol (1.00 eq.) lithium diisopropylamide in 5 ml THF was added dropwise. The reaction mixture was stirred for 2 h in the cold, before 1,2-bis(dicyanomethylene)-3-triethylammonium-cyclopropanid **2** (200 mg. 0.75 mmol, 1.00 eq.) dissolved in 6 ml dry acetonitrile were added dropwise. The yellow solution was slowly allowed to warm to room temperature overnight. The solution was filtered over a fiberglass filter and concentrated under reduced pressure. The residue was dissolved in 5-10 ml water and TDACl·H_2O (167 mg, 0.75 mmol, 1.00 eq.) in 3 ml water was added. The resulting precipitate was filtered off, washed with water and dissolved in DCM. The organic phase was dried over magnesium sulfate and filtered. The crude product was purified with column chromatography using neutral allox and a solvent mixture of DCM:MeOH = 30:1 (without applying additional pressure!). The product **6a** could be isolated as yellow powder in 62% yield (245 mg, 0.47 mmol).

^{1}H-NMR (chloroform-d, 300 MHz, 296 K): δ = 7.56 (d, *J* = 1.7 Hz, 1H), 7.12 (d, *J* = 1.7 Hz, 1H), 3.17 (s, 18H) ppm.

^{13}C-NMR (chloroform-*d*, 75 MHz, 296 K): δ = 134.38 (imidazole-C^4), 127.21 (C^1 and C^2), 125.58 (imidazole-C^5), 119.23 ($C(\underline{C}N)_2$), 118.39 ($C(\underline{C}N)_2$), 117.72 (TDA-C_3), 110.81 (C^3), 88.55 (imidazole-C^2), 42.68 (TDA-$\underline{C}H_3$), 33.28 ($\underline{C}(CN)_2$) ppm.

Elemental Analysis: calc.: C: 48.01, H: 3.84, N: 24.00
found: C: 47.64, H: 3.872, N: 23.95.

MS (ESI): positive mode: *m/z* = 168.00, negative mode: product anion could not be detected

IR: signal (intensity) = 3150 (vw) [C-H],3123 (w) [C-H], 3009 (vw) [C-H, TDA], 2970 (w) [C-H, TDA], 2930 (m) [C-H, TDA], 2897 (m) [C-H, TDA], 2803 (w) [C-H, TDA], 2361 (w), 2208 (w) [CN], 2199 (vs) [CN], 2181 (vs) [CN], 1896 (w), 1823 (vw), 1738 (m). 1555 (vs), 1474 (vs), 1435 (vs), 1402 (vs), 1323 (s), 1302 (m), 1269 (m), 1215 (s), 1412 (m), 1123 (m), 1099 (m), 1057 (w), 1030 (m), 1011 (s), 949 (w), 899 (w), 876 (w), 841 (vw), 787 (s), 735 (s), 669 (m), 648 (s), 610 (m), 581 (s), 559 (w), 523 (s), 478 (w), 447 (m) cm^{-1}.

R_f = 0.42 (DCM:MeOH = 30:1, on neutral allox)

B.5) Tetrabutylammonium 1,2-bis(dicyanomethylene)-3-(2-iodoimidazolyl)-cyclopropanid 6b

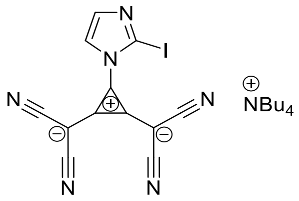

Chemical Formula: $C_{28}H_{39}N_7$
Molecular Weight: 473,67

Under an argon atmosphere146 mg (0.75 mmol, 1.00 eq.) 2-iodominidazole **5** were dissolved in 15 ml dry THF and cooled to -78 °C. A cooled solution of 0.75 mmol (1.00 eq.) lithium diisopropylamide in 5 ml THF was added dropwise. The reaction mixture was stirred for 2 h in the cold, before 1,2-bis(dicyanomethylene)-3-triethylammonium-cyclopropanid **2** (200 mg. 0.75 mmol, 1.00 eq.) dissolved in 6 ml dry acetonitrile were added dropwise. The yellow solution was slowly warmed up to room temperature overnight. The solvent was removed under reduced pressure and the residue was dissolved in 5-10 ml water. The aqueous solution was filtered over a fiberglass filter. Afterwards, tetrabutylammonium chloride (209 mg, 0.75 mmol, 1.00 eq.) dissolved in 5 ml water was added and the resulting precipitate was filtered off, washed with water and dissolved in DCM. The organic phase was dried over magnesium sulfate and the crude product was purified using flash column chromatography using silica and DCM:MeOH-mixture (20:1). The resulting solid was dissolved in a few millilitres DCM and diethyl ether was added until turbidity was observed. Since the formation of a precipitate could not be observed, DCM was slowly added until the turbidity vanished. Crystal formation suddenly started when reaching this point. The crystals where filtered off and dried in vacuo. The product **6b** was isolated as a white solid in 62% yield (280 mg, 0.46 mmol).

^{1}H-NMR (chloroform-*d*, 300 MHz, 296 K): δ = 7.55 (d, *J* = 1.7 Hz, 1H, imidazole-$C^5\underline{H}$), 7.10 (d, *J* = 1.7 Hz, 1H, imidazole-$C^4\underline{H}$), 3.24 – 3.06 (m, 8H, $N(C\underline{H}_2CH_2CH_2CH_3)_4$), 1.62 (dq, *J* = 11.5, 8.1, 7.7 Hz, 8H, $N(CH_2C\underline{H}_2CH_2CH_3)_4$), 1.44 (hept, *J* = 7.4 Hz, 8H, $N(CH_2CH_2C\underline{H}_2CH_3)_4$), 1.01 (t, *J* = 7.3 Hz, 12H, $N(CH_2CH_2CH_2C\underline{H}_3)_4$) ppm.

^{13}C-NMR (chloroform-*d*, 75 MHz, 296 K): δ = 134.25 (imidazole-C^4), 127.52 (C^1 and C^2), 125.58 (imidazole-C^5), 119.27 ($C(\underline{C}N)_2$), 118.60 ($C(\underline{C}N)_2$), 111.10 (C^3), 88.55 (imidazole-C^2), 58.93 ($N(\underline{C}H_2CH_2CH_2CH_3)_4$), 33.28 ($\underline{C}(CN)_2$), 23.95 ($N(CH_2\underline{C}H_2CH_2CH_3)_4$), 19.81 ($N(CH_2CH_2\underline{C}H_2CH_3)_4$), 13.71 ($N(CH_2CH_2CH_2\underline{C}H_3)_4$) ppm.

Elemental Analysis: calc.: C: 56.09, H: 6.39, N: 16.35
found: C: 56.12, H: 6.309, N: 16.58.

MS (ESI): positive mode: *m/z* = 242.14, negative mode: product anion could not be detected

IR: signal (intensity) = 2963 (m) [C-H], 2932 (m) [C-H], 2874 (m) [C-H], 2444 (vw), 2359 (w), 2205 (vs) [CN], 2189 (vs) [CN], 2178 (s) [CN], 2160 (w), 1931 (w), 1911 (w), 1888 (w), 1738 (w), 1597 (vw), 1520 (w), 1474 (vs), 1437 (vs), 1416 (s), 1381 (s), 1321 (s), 1298 (w), 1271 (m), 1219 (w), 1188 (m), 1169 (w), 1157 (w), 1144 (m), 1119 (m), 1097 (m), 1086 (m), 1028 (w), 1011 (s), 928 (vw), 883 (m), 795 (m), 743 (s), 669 (w), 648 (s), 608 (w), 583 (s), 530 (m), 519 (m), 476 (w), 463 (w), 444 (m) cm^{-1}.

R_f = 0.35 (DCM:MeOH = 20:1)

II. NMR- and IR-Spectra

1,2-Bis(dicyanomethylene)-3-triethylammonium-cyclopropanid 2

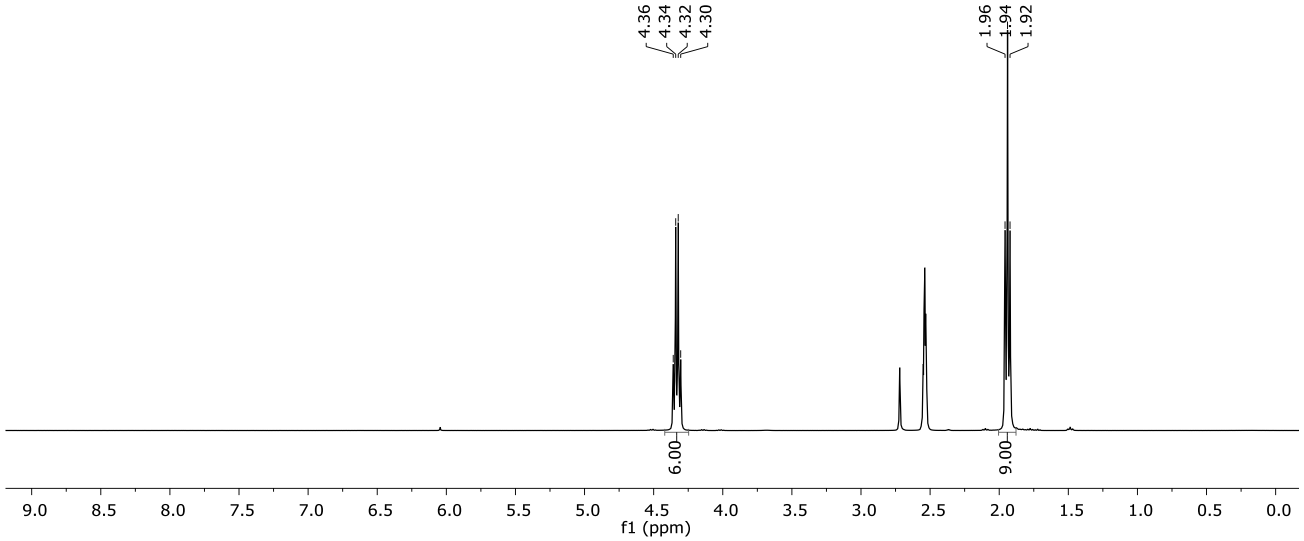

Figure S1: ^{1}H-NMR spectrum of 1,2-Bis(dicyanomethylene)-3-triethylammonium-cyclopropanid **2** in deuterated acetonitrile.

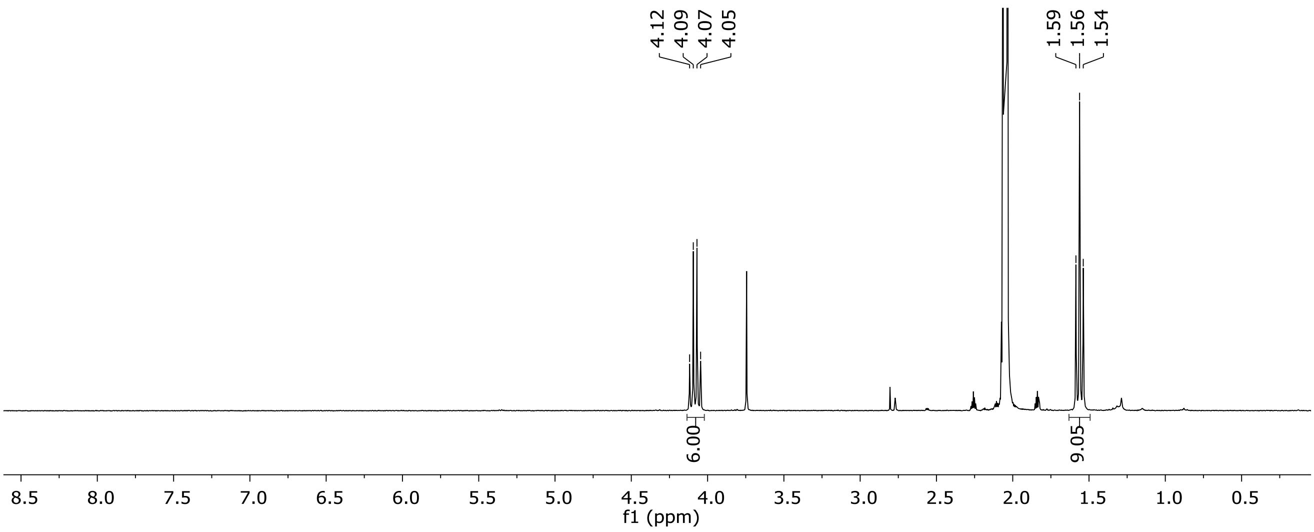

Figure S2. ^{1}H-NMR spectrum of 1,2-Bis(dicyanomethylene)-3-triethylammonium-cyclopropanid **2** in deuterated acetone.

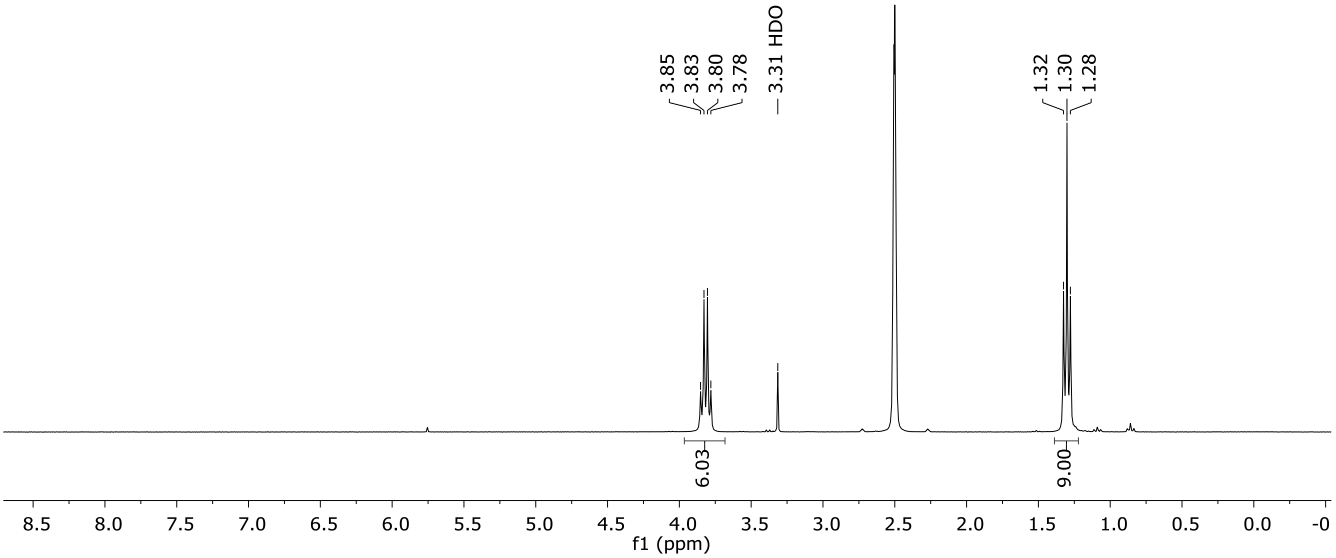

Figure S3. ^{1}H-NMR spectrum of 1,2-Bis(dicyanomethylene)-3-triethylammonium-cyclopropanid **2** in deuterated acetone.

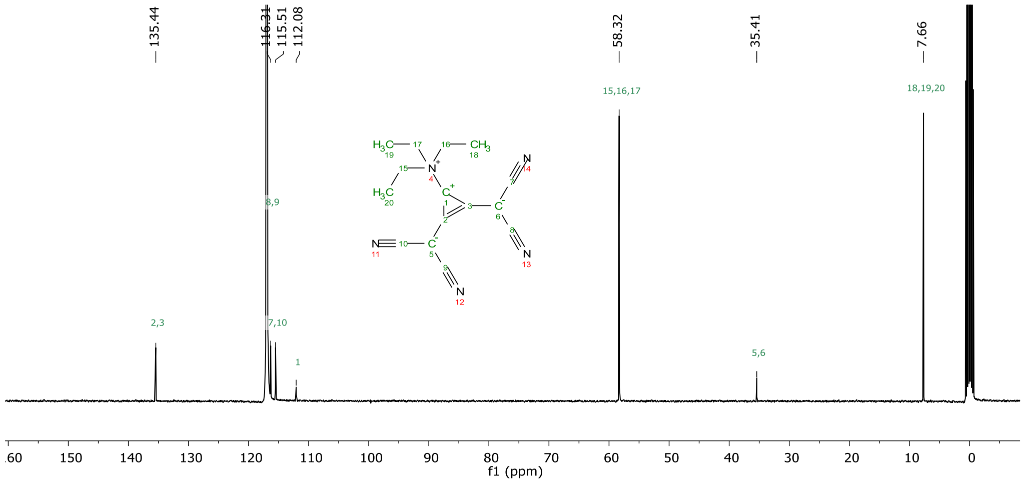

Figure S4. ^{13}C-NMR spectrum of 1,2-Bis(dicyanomethylene)-3-triethylammonium-cyclopropanid **2** in deuterated acetonitrile.

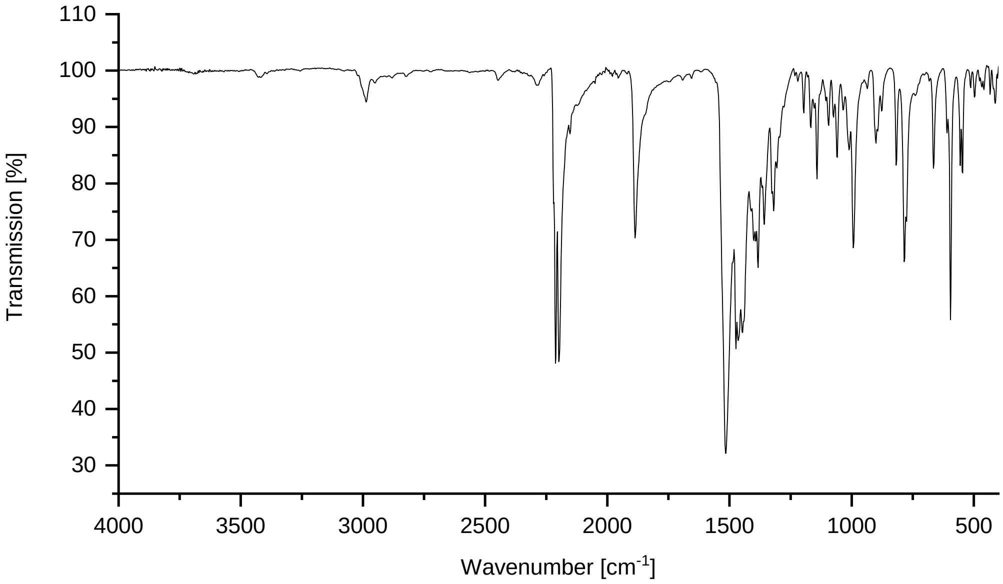

Figure S5. IR-Spectrum of 1,2-bis(dicyanomethylene)-3-triethylammoniumcyclopropanid **2**.

Tris(dimethylamino)cyclopropenylium 1,2-bis(dicyanomethylene)-3-hydro-cyclopropanid 3

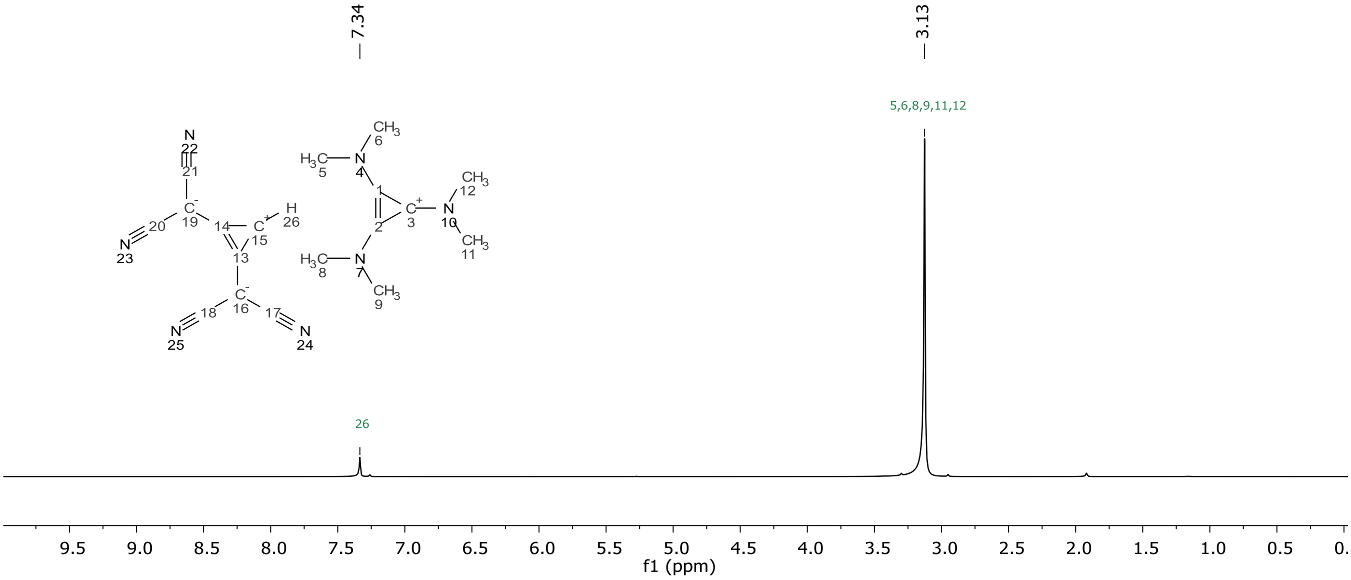

Figure S6. ^{1}H-NMR spectrum of tris(dimethylamino)cyclopropenylium 1,2-bis(dicyanomethylene)-3-hydro-cyclopropanid **3** in deuterated chloroform.

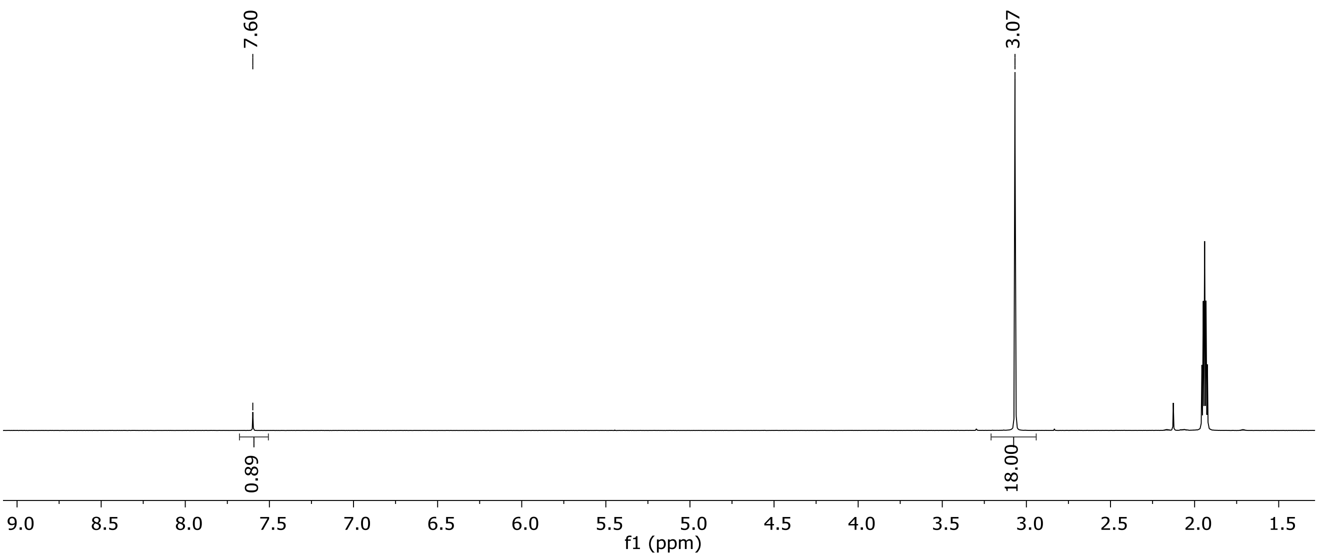

Figure S7. ^{1}H-NMR spectrum of tris(dimethylamino)cyclopropenylium 1,2-bis(dicyanomethylene)-3-hydro-cyclopropanid **3** in deuterated acetonitrile.

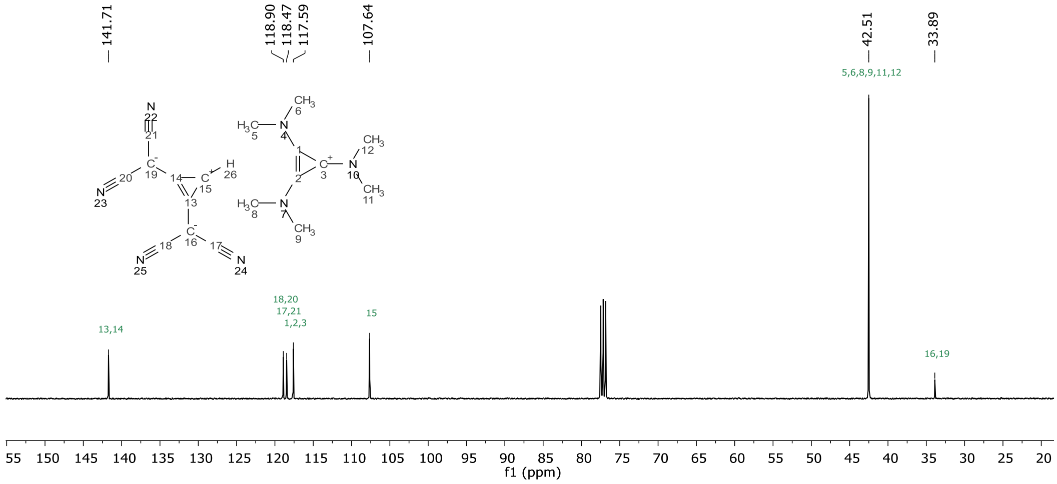

Figure S8. ^{13}C-NMR spectrum of tris(dimethylamino)cyclopropenylium 1,2-bis(dicyanomethylene)-3-hydro-cyclopropanid **3** in deuterated chloroform.

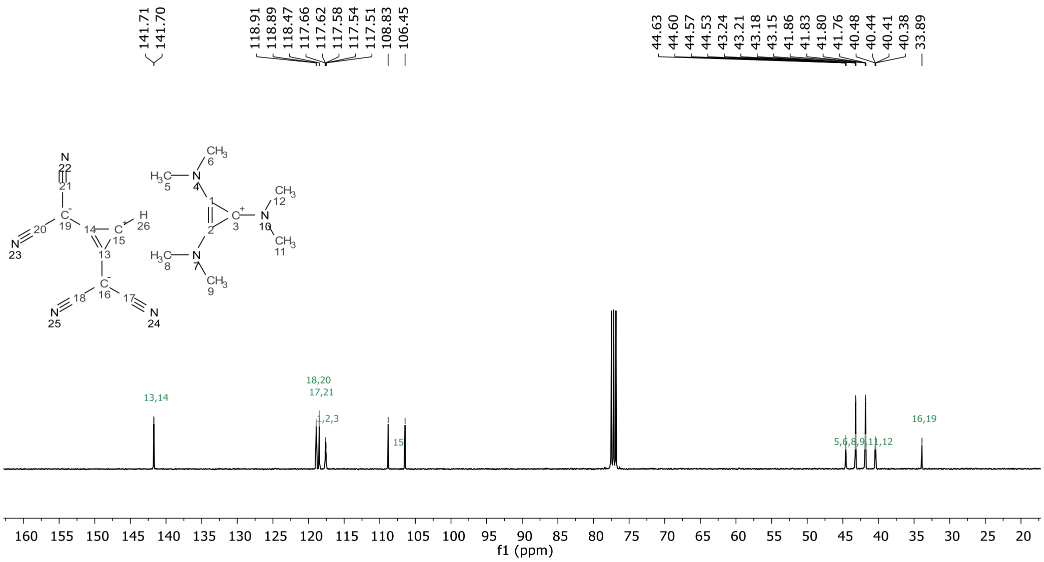

Figure S9. ^{13}C-NMR spectrum of tris(dimethylamino)cyclopropenylium 1,2-bis(dicyanomethylene)-3-hydro-cyclopropanid **3** in deuterated chloroform showing CH-couplings.

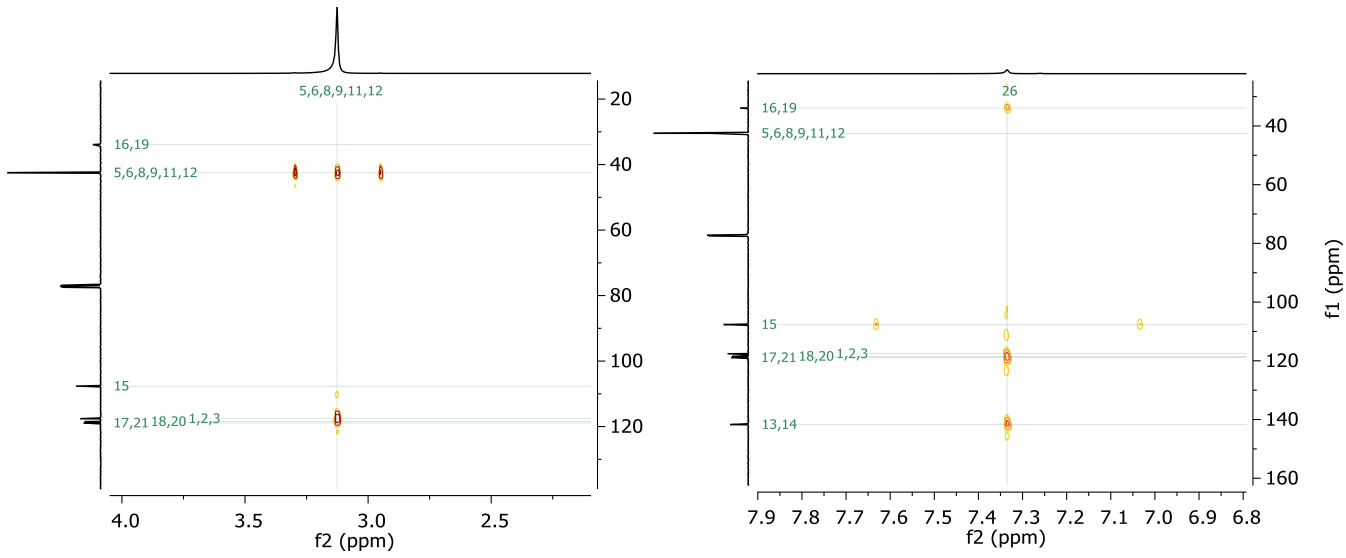

Figure S10. HMBC-NMR spectrum of tris(dimethylamino)cyclopropenylium 1,2-bis(dicyanomethylene)-3-hydro-cyclopropanid **3** in deuterated chloroform.

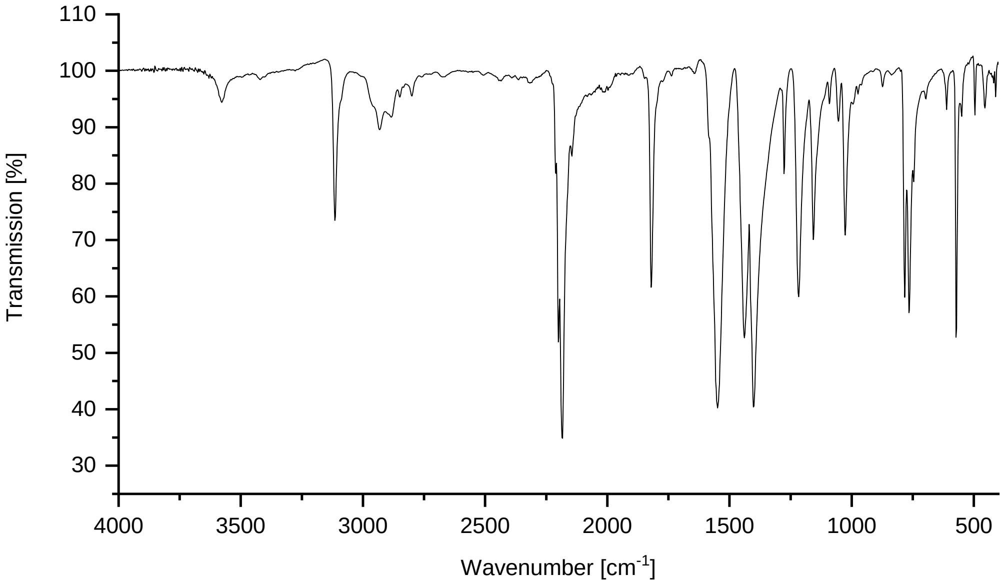

Figure S11. IR-Spectrum of tris(dimethylamino)cyclopropenylium 1,2-bis(dicyanomethylene)-3-hydro-cyclopropanid **3.**

Tetrabutylammonium 1,2-bis(dicyanomethylene)-3-hydro-cyclopropanid 3•NBu$_4$

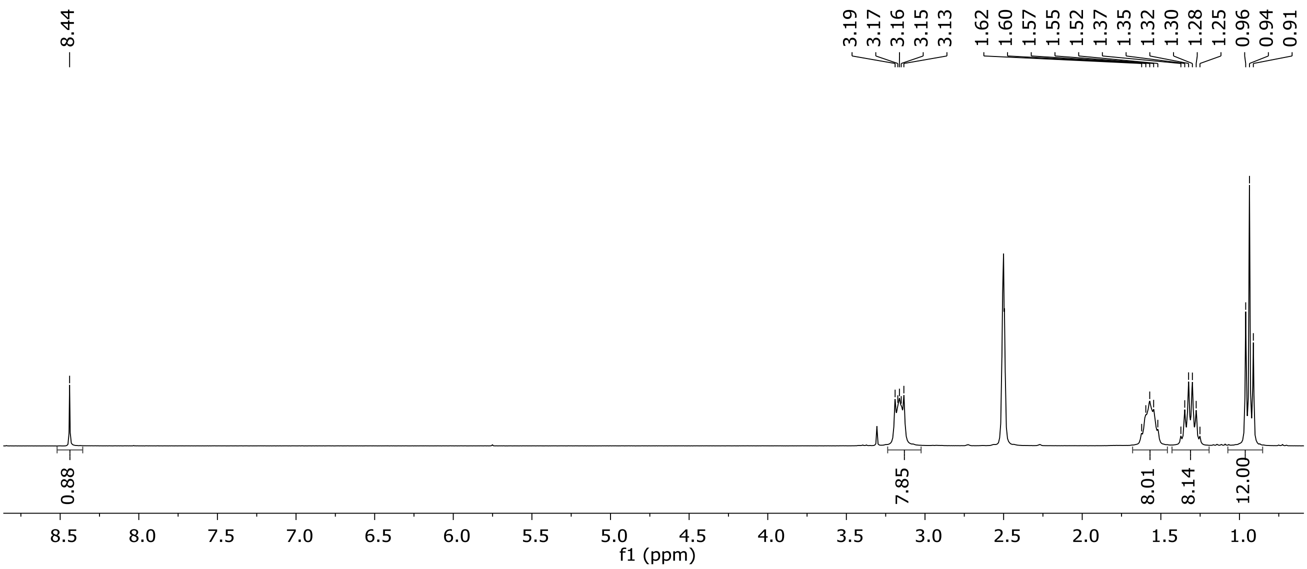

Figure S12. ^{1}H-NMR spectrum of tetrabutylammonium 1,2-bis(dicyanomethylene)-3-hydro-cyclopropanid **3•NBu$_4$** in deuterated DMSO.

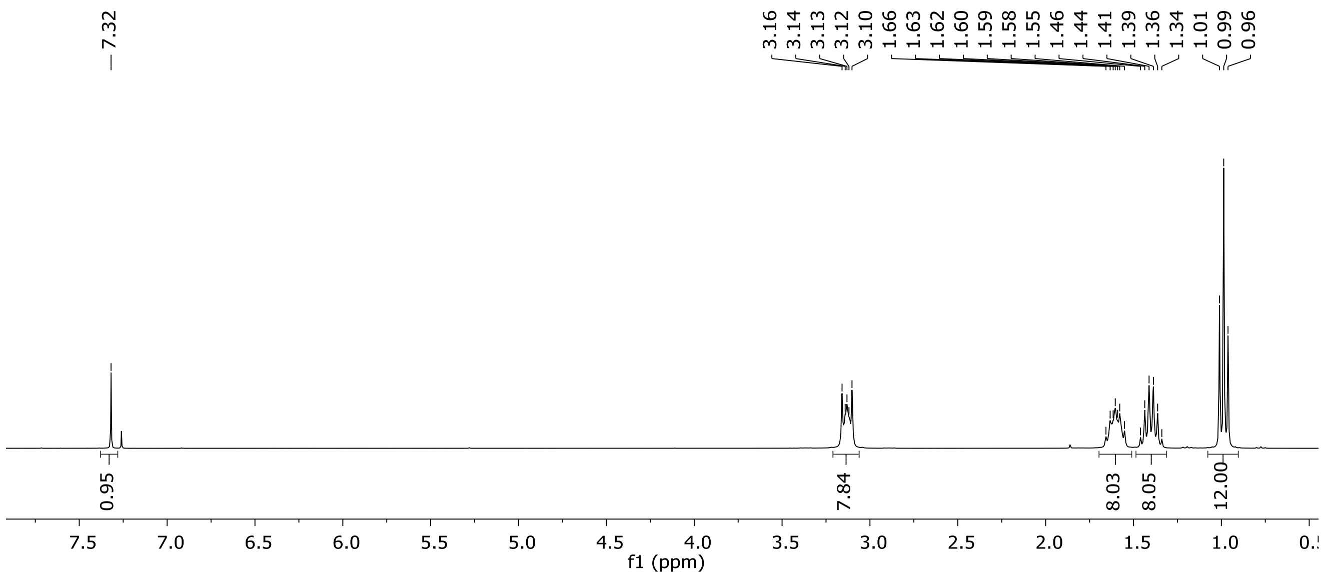

Figure S13. ^{1}H-NMR spectrum of tetrabutylammonium 1,2-bis(dicyanomethylene)-3-hydro-cyclopropanid **3•NBu$_4$** in deuterated chloroform.

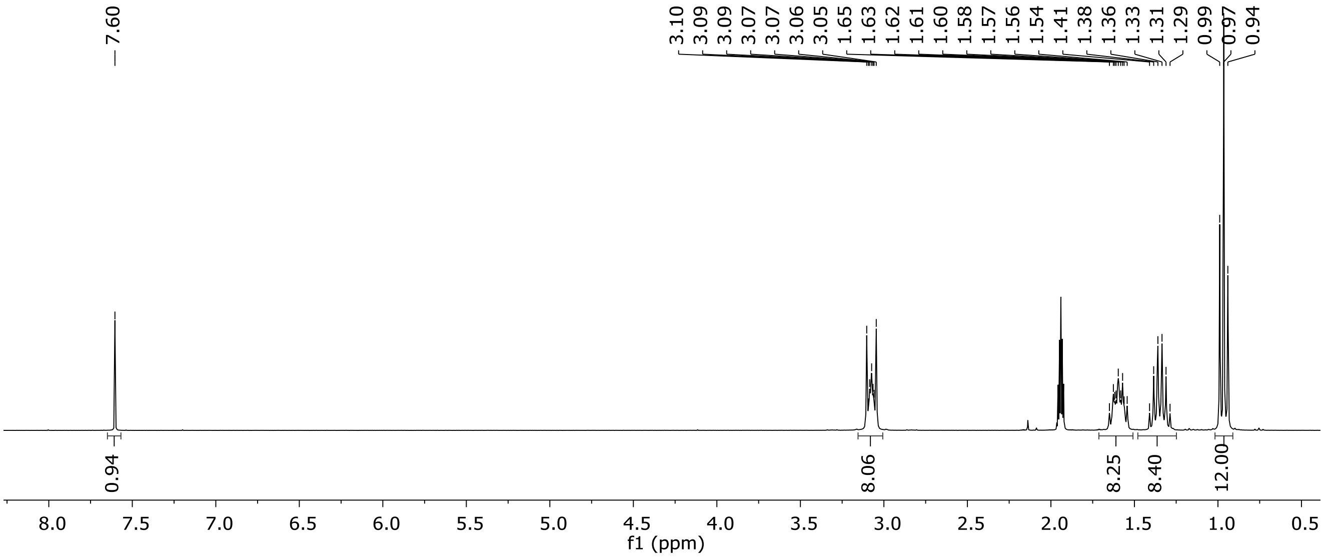

Figure S14. ^{1}H-NMR spectrum of tetrabutylammonium 1,2-bis(dicyanomethylene)-3-hydro-cyclopropanid **3•NBu$_4$** in deuterated acetonitrile.

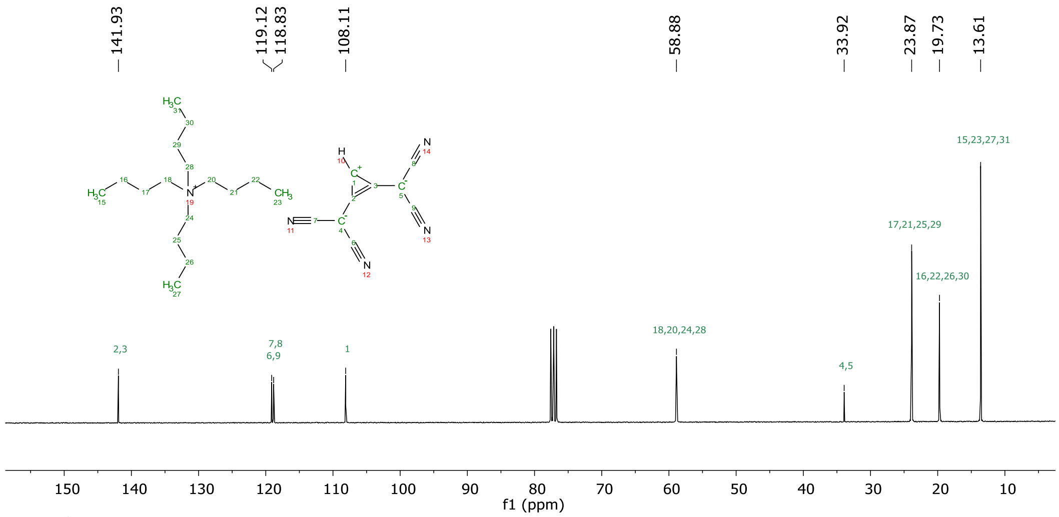

Figure S15. ^{13}C-NMR spectrum of tetrabutylammonium 1,2-bis(dicyanomethylene)-3-hydro-cyclopropanid **3•NBu$_4$** in deuterated chloroform.

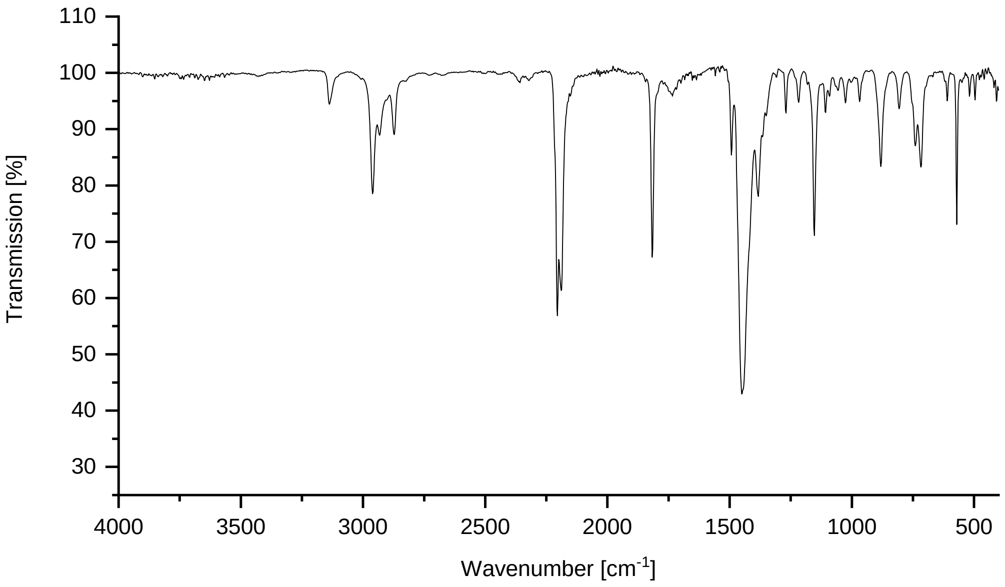

Figure S16. IR-Spectrum of Tetrabutylammonium 1,2-bis(dicyanomethylene)-3-hydro-cyclopropanid **3b.**

Tris(dimethylamino)cyclopropenylium 1,2-bis(dicyanomethylene)-3-iodo-cyclopropanid 1

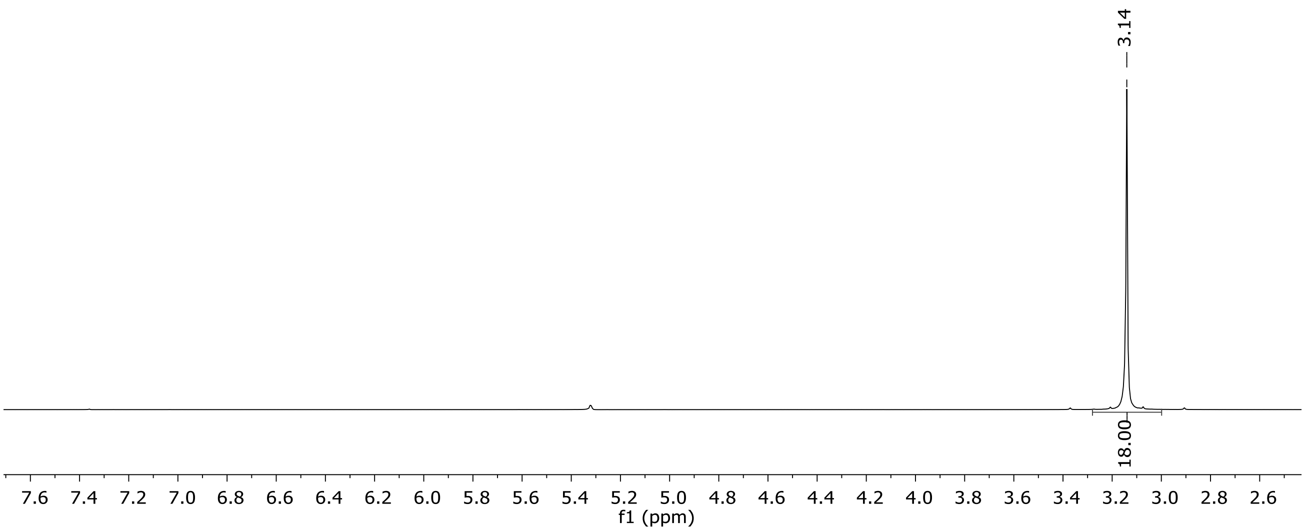

Figure S17. ^{1}H-NMR spectrum of tris(dimethylamino)cyclopropenylium 1,2-bis(dicyanomethylene)-3-iodo-cyclopropanid **1** in deuterated dichloromethane.

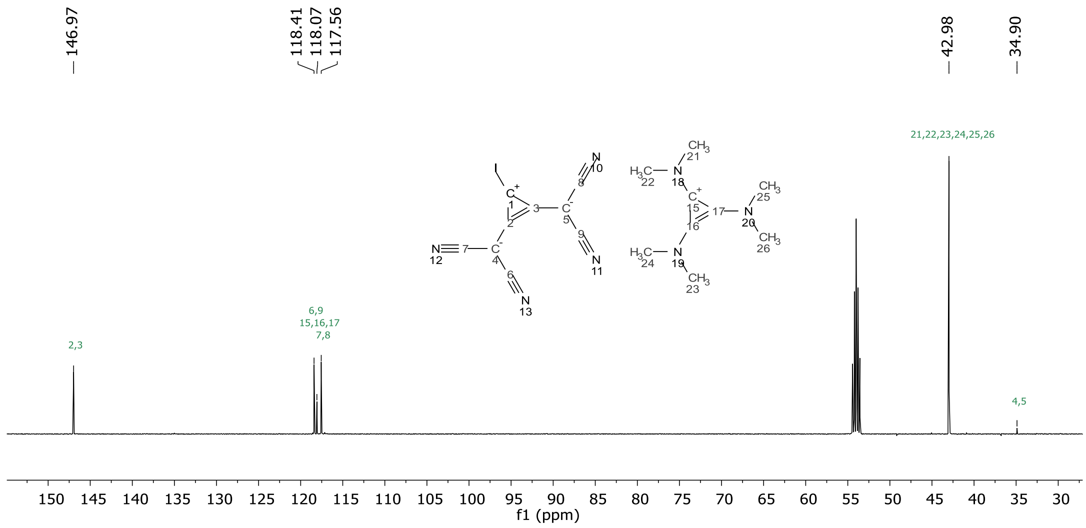

Figure S18. ^{13}C-NMR spectrum of tris(dimethylamino)cyclopropenylium 1,2-bis(dicyanomethylene)-3-iodo-cyclopropanid **1.** The signals for the C1 carbon could not be detected due to the low intensity of the signal. This spectrum was obtained using a cryoprobe.

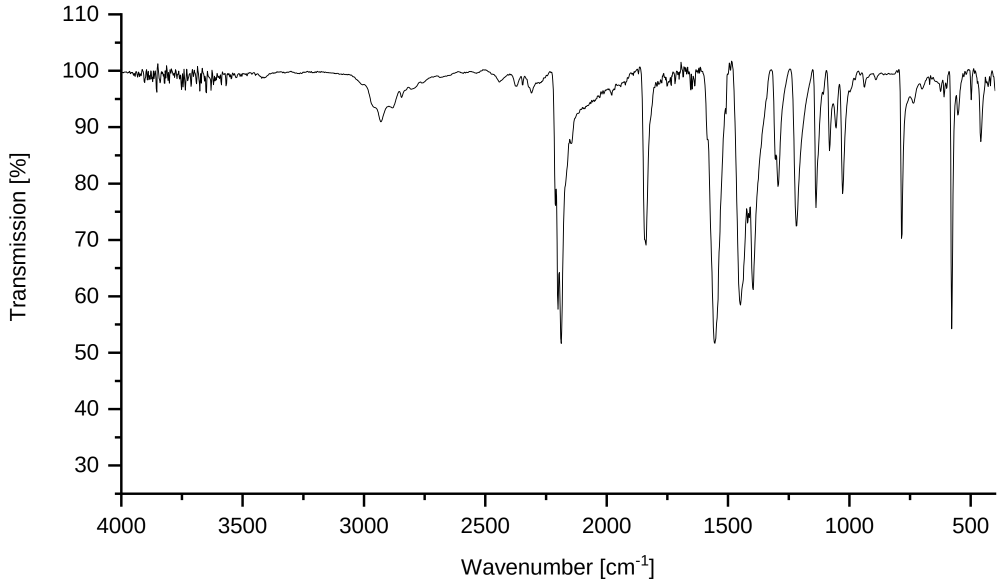

Figure S19. IR-Spectrum of tris(dimethylamino)cyclopropenylium 1,2-bis(dicyanomethylene)-3-iodo-cyclopropanid **1**.

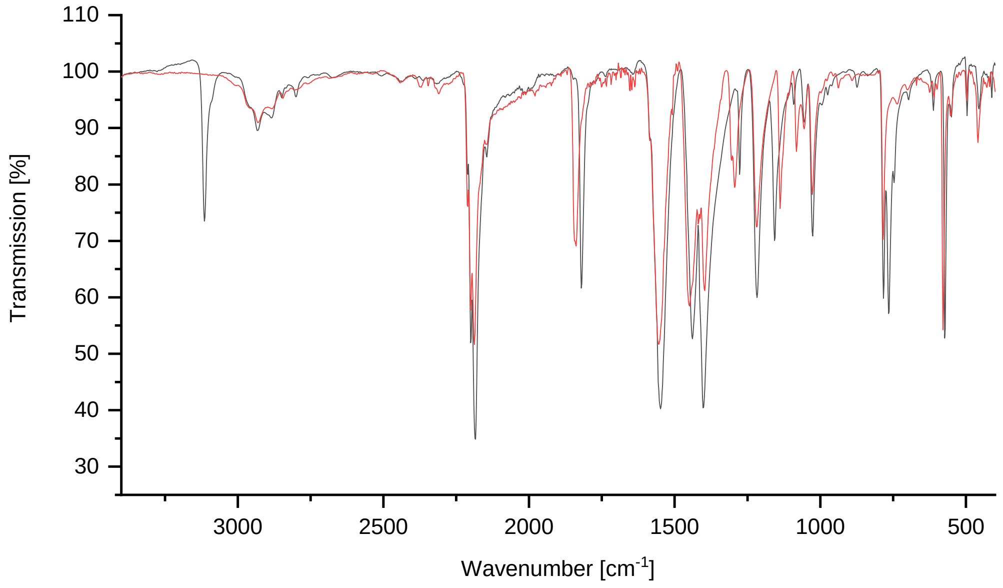

Figure S20. IR-Spectrum of tris(dimethylamino)cyclopropenylium 1,2-bis(dicyanomethylene)-3-iodo-cyclopropanid **1** (red) and of the starting material **3** (black).

Tris(dimethylamino)cyclopropenylium 1,2-bis(dicyanomethylene)-3-(2-iodoimidazolyl)-cyclopropanid 6a

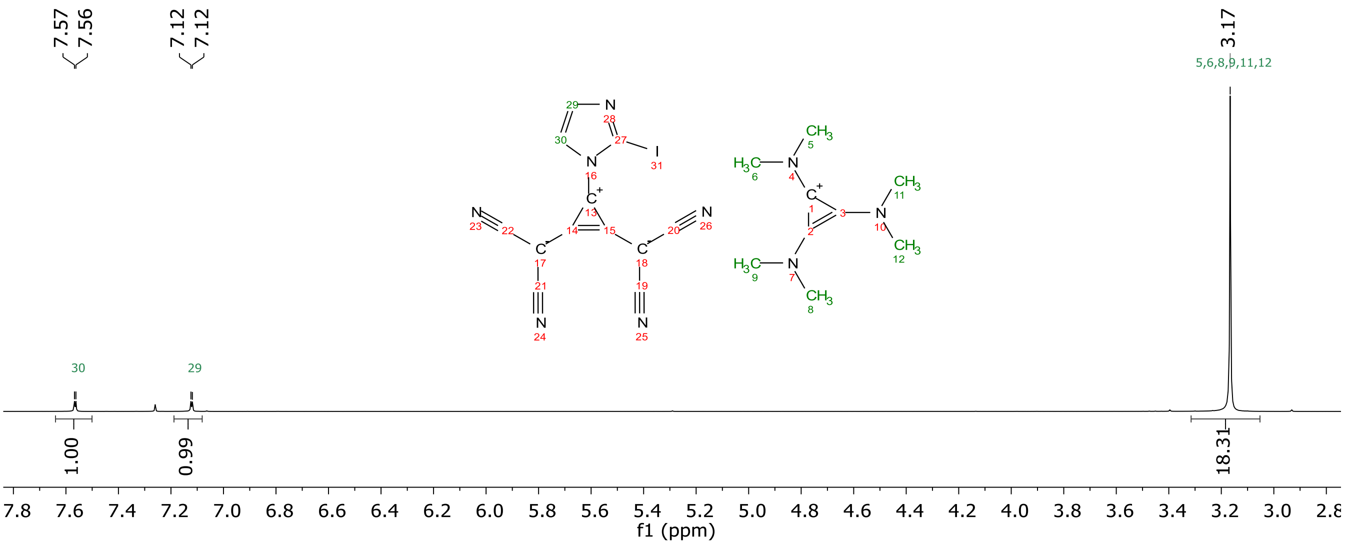

Figure S21. ^{1}H-NMR of tris(dimethylamino)cyclopropenylium 1,2-bis(dicyanomethylene)-3-(2-iodoimidazolyl)-cyclopropanid **6a** in deuterated chloroform.

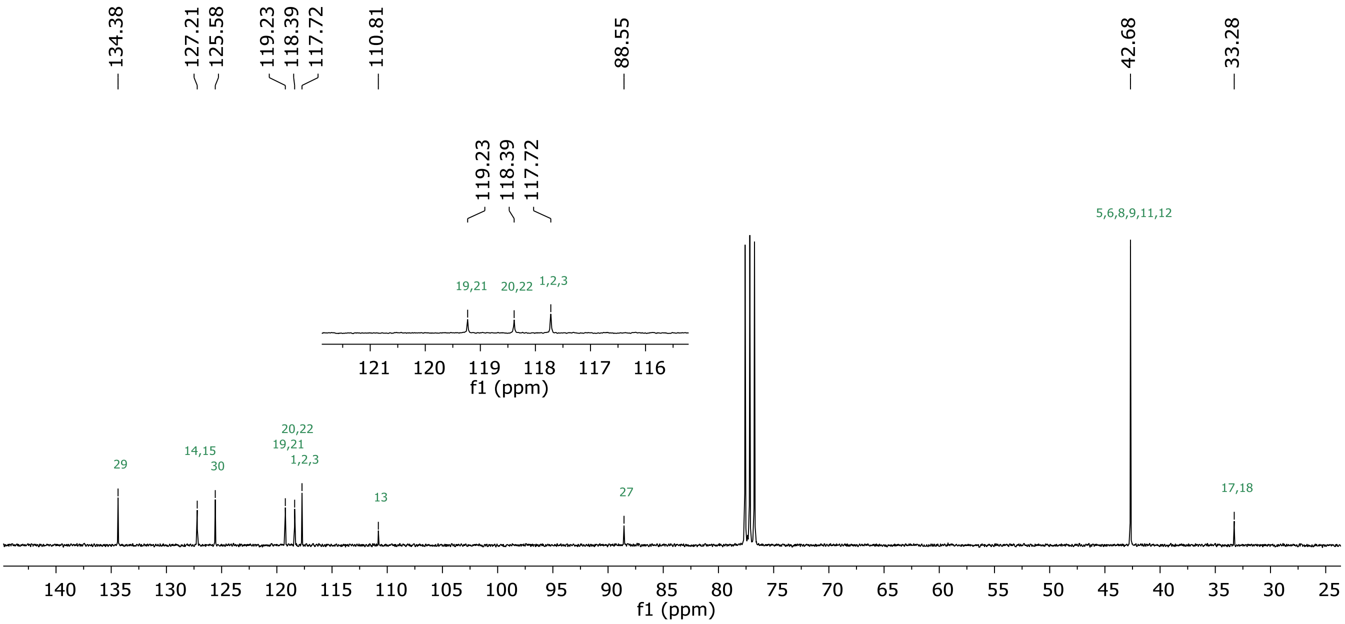

Figure S22. ^{13}C-NMR of tris(dimethylamino)cyclopropenylium 1,2-bis(dicyanomethylene)-3-(2-iodoimidazolyl)-cyclopropanid **6a** in deuterated chloroform.

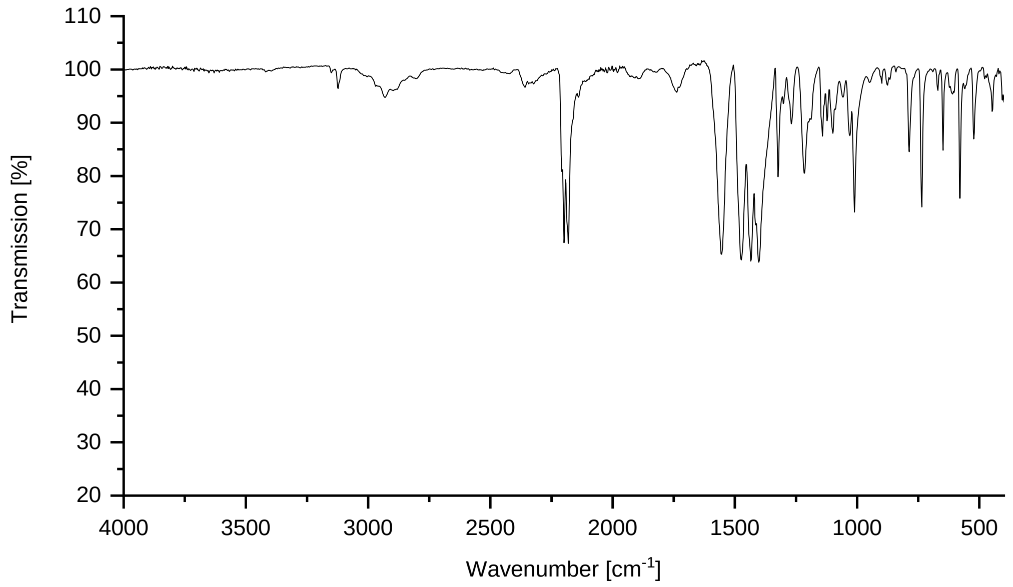

Figure S23. IR-Spectrum of tris(dimethylamino)cyclopropenylium 1,2-bis(dicyanomethylene)-3-(2-iodoimidazolyl)-cyclopropanid **6a**.

Tetrabutylammonium 1,2-bis(dicyanomethylene)-3-(2-iodoimidazolyl)-cyclopropanid 6b

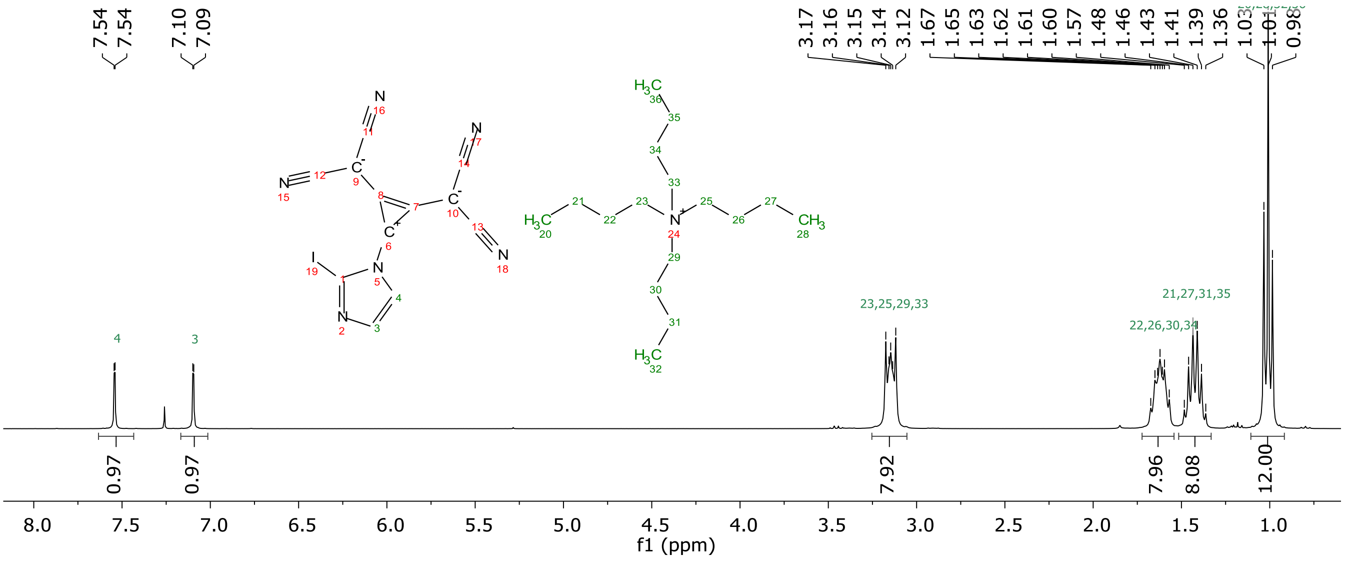

Figure S24. ^{1}H-NMR spectrum of tetrabutylammonium 1,2-bis(dicyanomethylene)-3-(2-iodoimidazolyl)-cyclopropanid **6b** in deuterated chloroform.

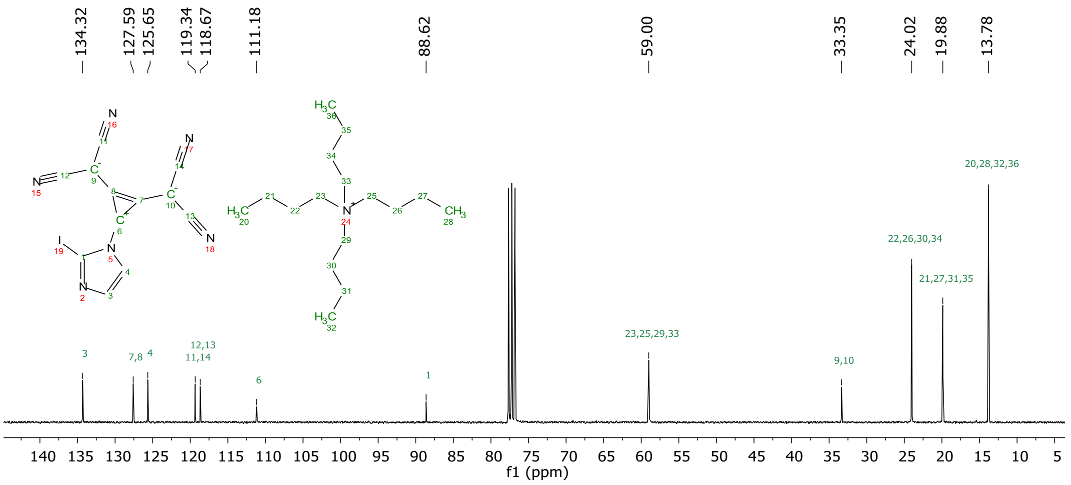

Figure S25. ^{13}C-NMR spectrum of tetrabutylammonium 1,2-bis(dicyanomethylene)-3-(2-iodoimidazolyl)-cyclopropanid **6b** in deuterated chloroform.

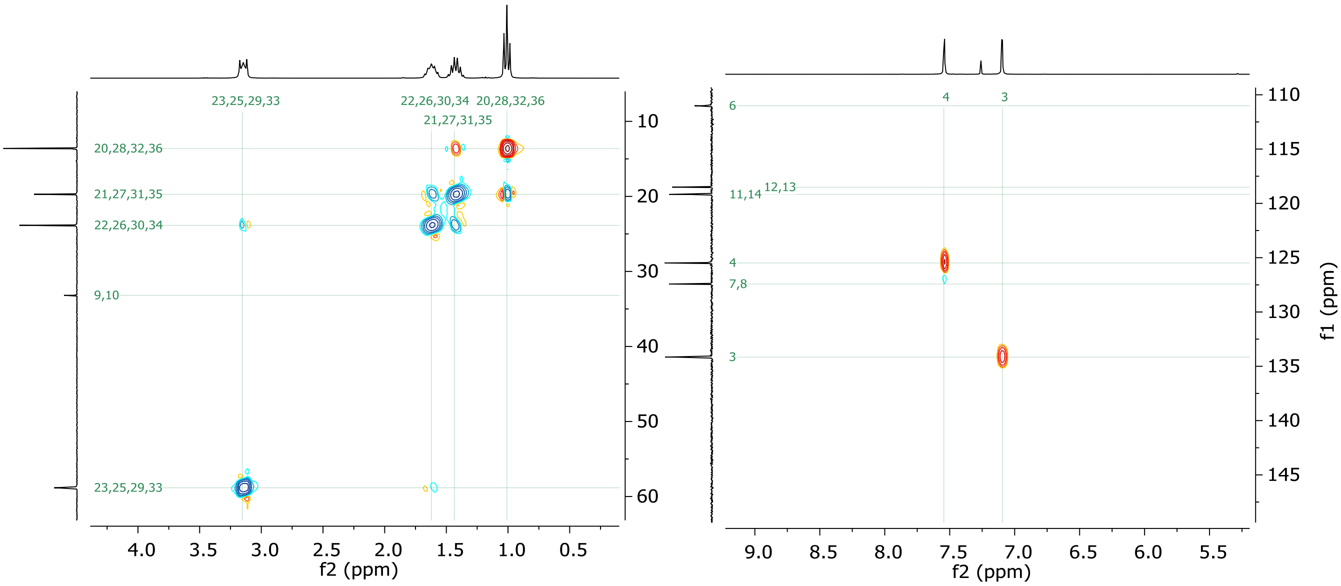

Figure S26. ^{1}H-^{13}C-HSQC NMR spectrum of tetrabutylammonium 1,2-bis(dicyanomethylene)-3-(2-iodoimidazolyl)-cyclopropanid **6b** in deuterated chloroform.

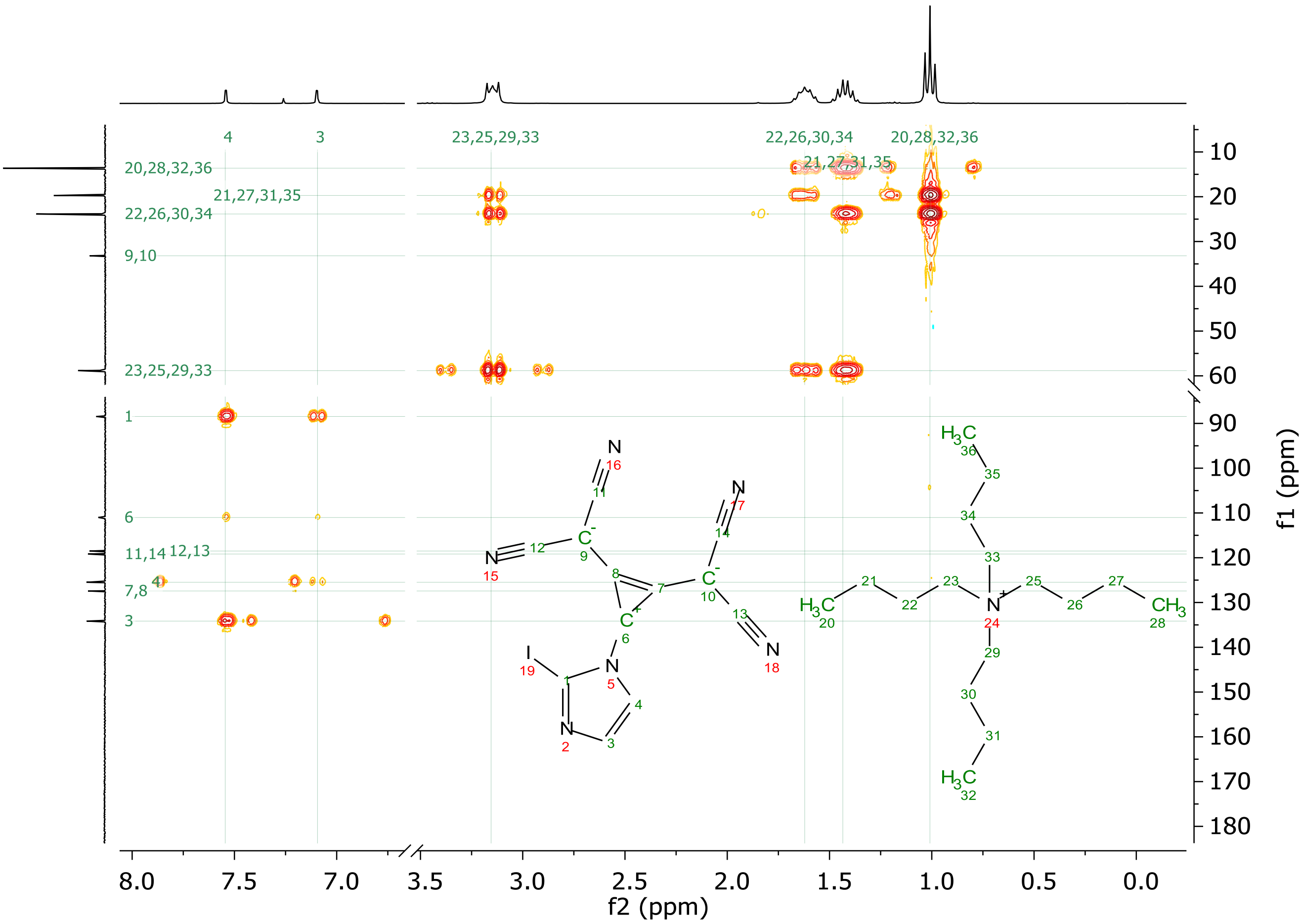

Figure S27. ^{1}H-^{13}C-HMBC NMR spectrum of tetrabutylammonium 1,2-bis(dicyanomethylene)-3-(2-iodoimidazolyl)-cyclopropanid **6b** in deuterated chloroform.

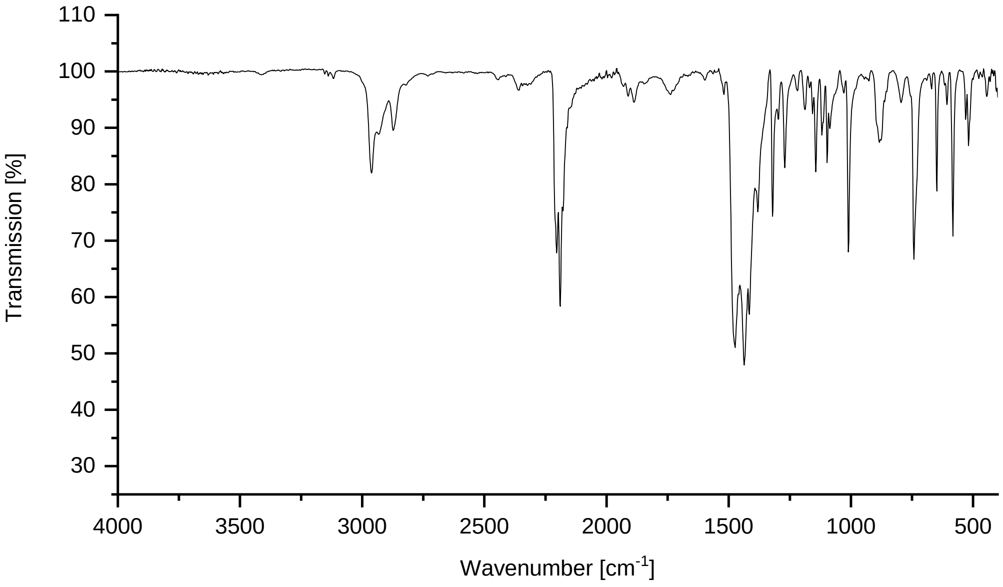

Figure S28. IR-Spectrum of tetrabutylammonium 1,2-bis(dicyanomethylene)-3-(2-iodoimidazolyl)-cyclopropanid **6b**.

III. X-Ray Structures

Single crystals were analysed on a *Rigaku* XtaLAB mini, equipped with a 600 W Mo micro-fine focus glass sealed tube, graphite monochromator (Mo K_α) and CCD detector or on a *Rigaku* Synergy dual source device, with Co and Mo micro focus sealed tubes (Cu & Mo K_α) using mirror monochromators and a HyPix-6000HE: Hybrid photon counting X-ray detector. Crystals were mounted in *Hampton* CrypLoops using Parabar/Paratone or *GE/Bayer* silicone grease. The crystals were cooled in a stream of cold nitrogen (Synergy) or with an *Oxford Crysosystems* Desktop Cooler, which cools pre-dried air to 170 K (mini). Data on *Rigaku* systems was recorded and reduced using the CrysalisPro[2] Software. Structures were solved using WinGX[3] in combination with ShelXT and refined with shelXle.[4] Graphics were generated using Diamond 4[5] or Mercury (3.6).

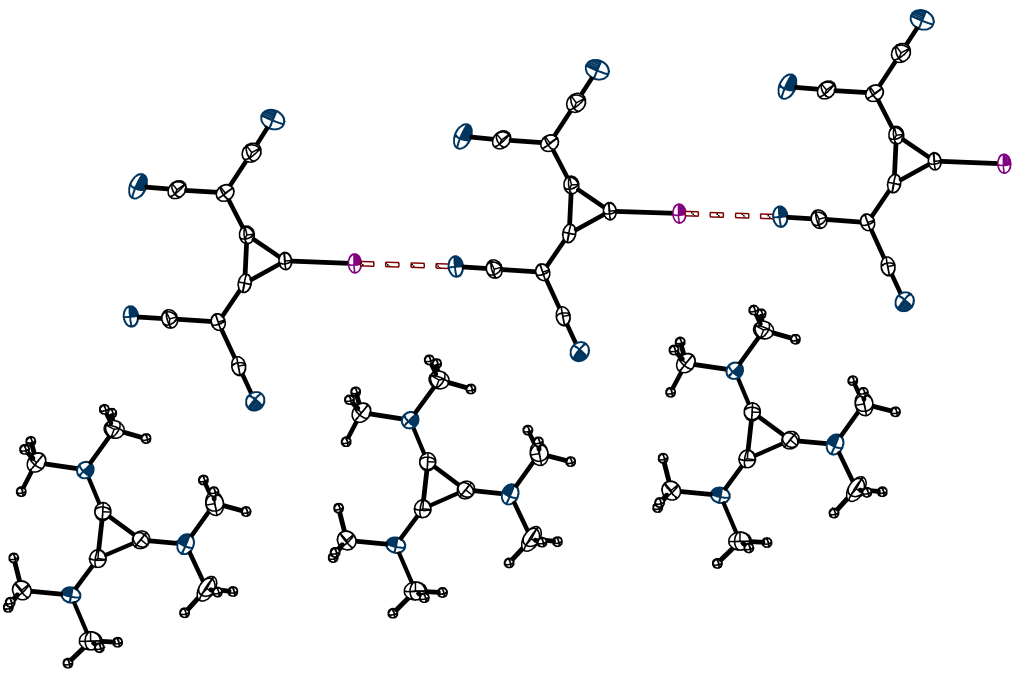

Figure S29. XB-bonded chains of iodine compound **1** alongside which the counterions are positioned. XB donor chains and bands of counterions are alternating thereby forming flat monolayers. Thermal ellipsoids at 50% probability level.

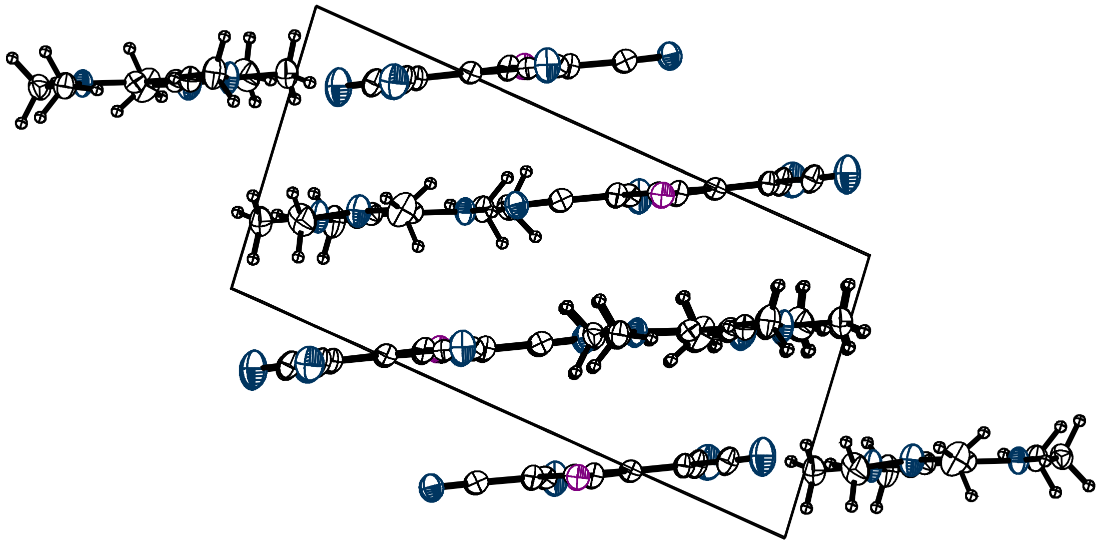

Figure S30. Packing in the unit cell found for **1**. Thermal ellipsoids at 50% probability level.

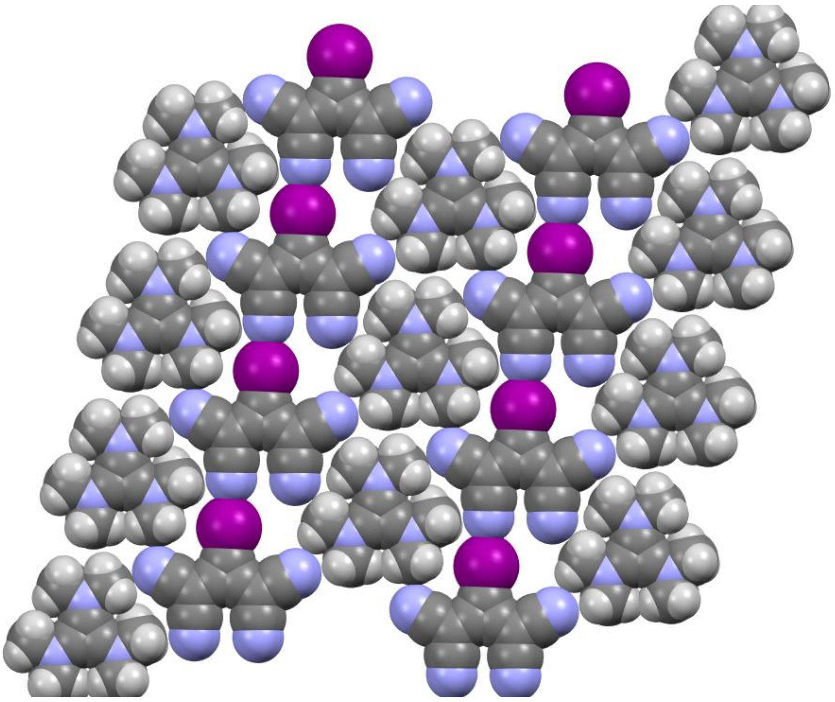

Figure S31. Coutout of a monolayer formed by XB-bonded chains of **1** and its counterions in the spacefilling model.

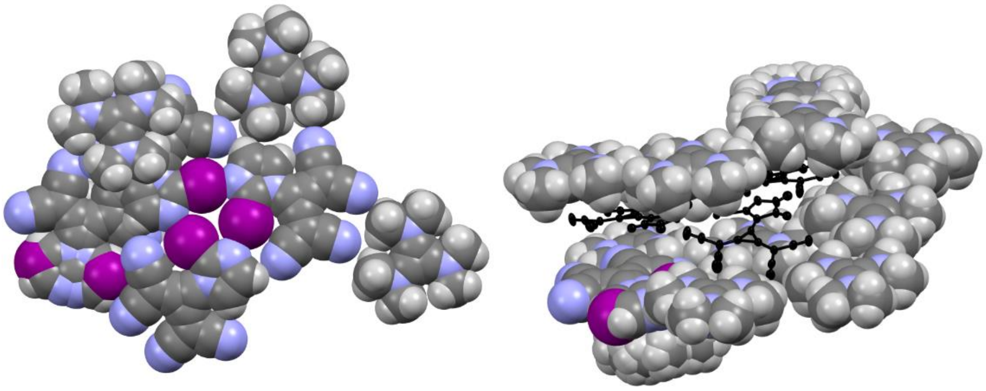

Figure S32. Left: Trimeric structure of **6a** together with the counterions (TDA+) and the additional XB donor molecule, which is not involved in the formation of halogen bonds (background left). It is positioned on the centre of symmetry and disordered. The last counterion is hidden behind this fourth imidazole molecule. Right: Trimer (black ellipsoids) in the "pocket" which is formed by the counterions.

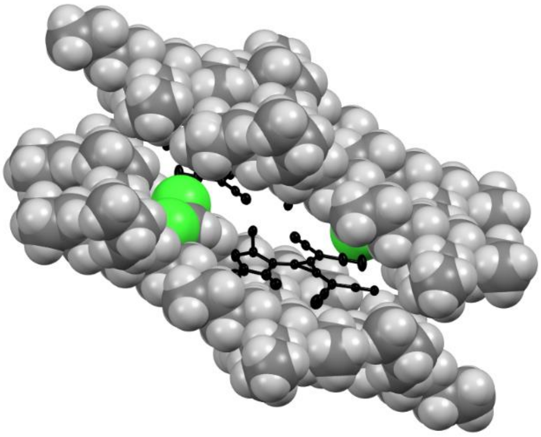

Figure S33. The dimeric structure of **6b** (black ellipsoids) is surrounded by eight weakly coordinating NBu_4^+ ions and DCM (both in the spacefilling model), which does not form any hydrogen bonds with the XB donor.

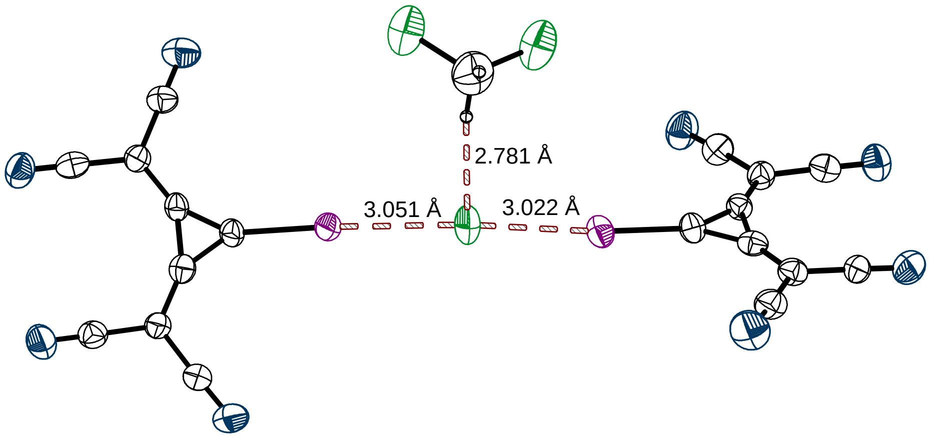

Figure S34. Analogue to the 2:1 co-crystal observed with iodide, this 2:1 co-crystal shows one shorter and one slightly elongated contact with the chloride/iodide as well as an additional hydrogen bond to a DCM molecule, which is incorporated in the crystal structure. The position of the chloride is partially occupied by an iodide ion (20%). Thermal ellipsoids at 50% probability level.

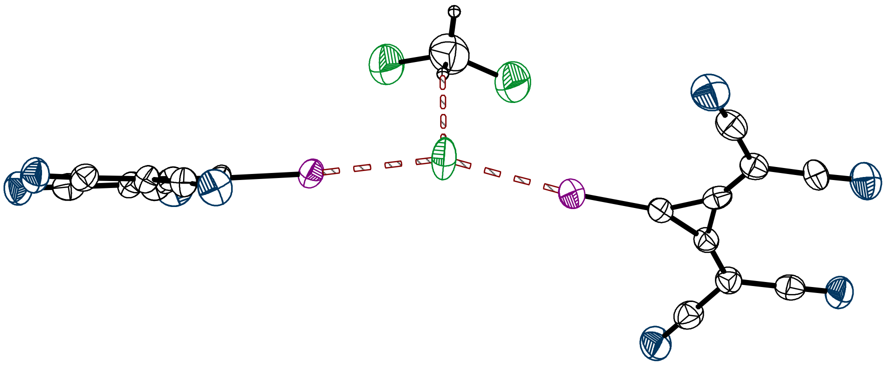

Figure S35. 2:1 co-crystal of **1** with Cl:I = 8:2. Thermal ellipsoids at 50% probability level.

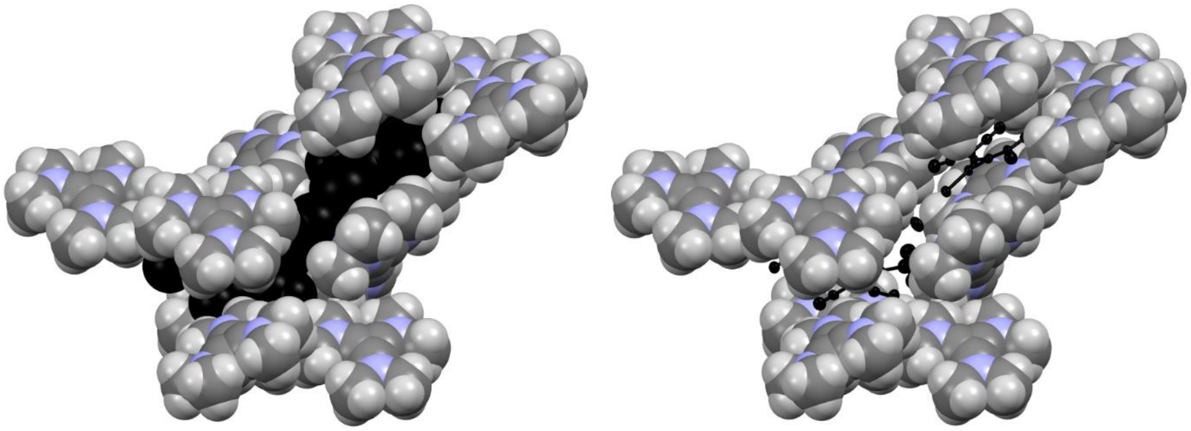

Figure S36. [**1**···Cl/I···**1**]$^{3-}$ with Cl:I = 8:2 surrounded by its counterions (in the spacefilling model). The XB donor and the halide are coloured in black.

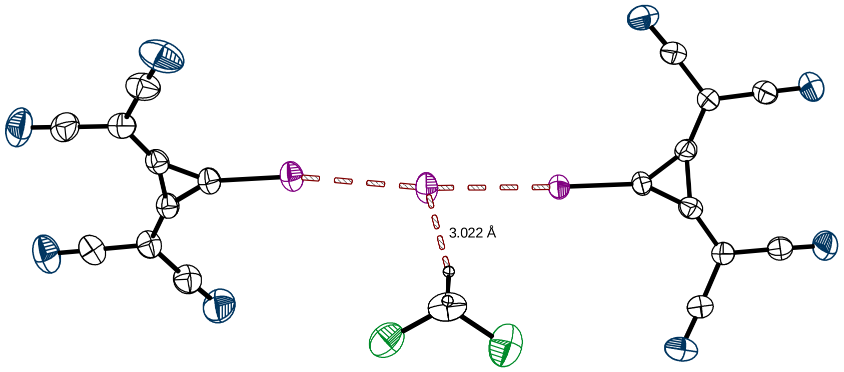

Figure S37. [**1**···I···**1**]$^{3-}$ co-crystal. Thermal ellipsoids at 50% probability level.

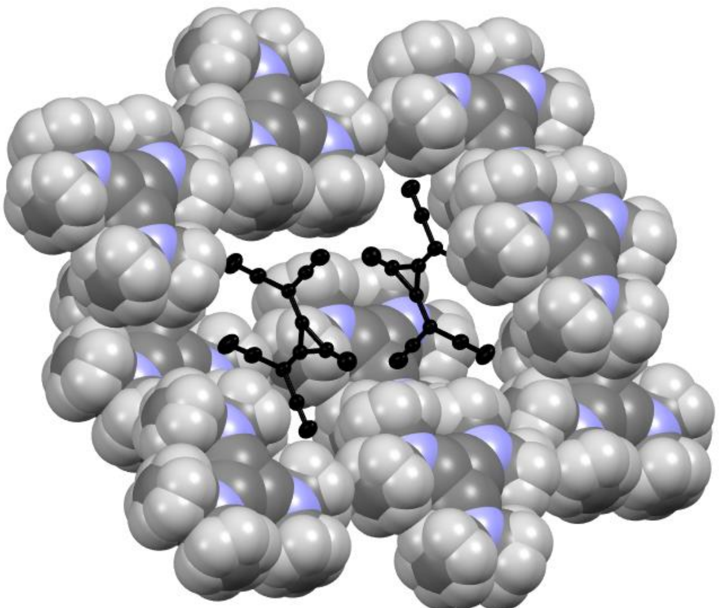

Figure S38. Dimer of H-compound **3** (black ellipsoids) "encapsulated" by 10 TDA-molecules in the spacefilling model (one capping the dimer from above is omitted for clarity): four lie within the same plane as the dimer, while the HB bonded dimer is sandwiched by another four (2 from above and 2 from below).

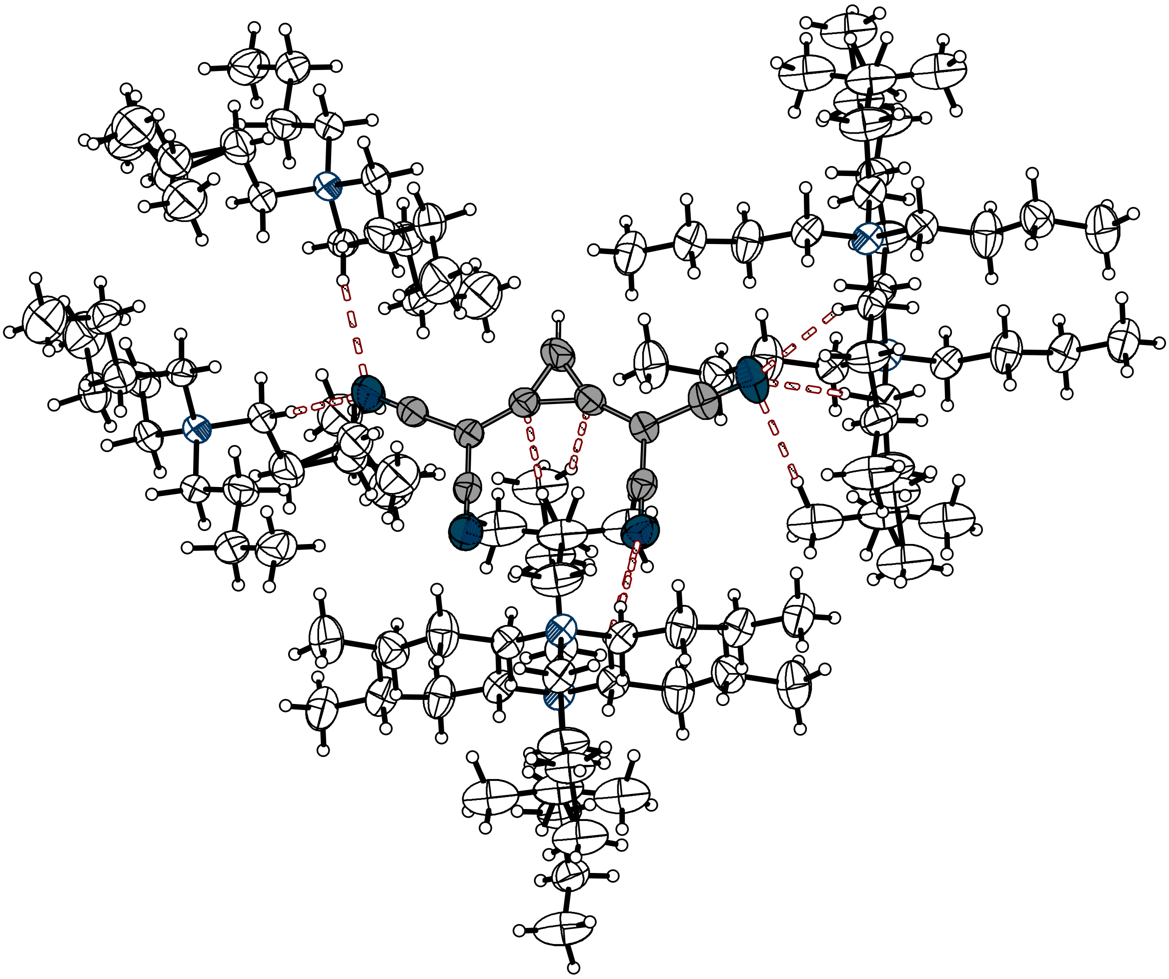

Figure S39. Tetrabutylammonium 1,2-bis(dicyanomethylene)-3-hydro-cyclopropanid surrounded by six tetrabutylammonium counterions. Contacts which are shorter than the sum of vdW radii are depicted. R_{HB} = 0.94-0.99 with an average of 0.97. For clarity one alkyl chain is omitted which would otherwise cover the anion. Thermal ellipsoids at 50% probability level.

Table S1. Crystal data and structure refinement for the the XB donors **1**, **6a** and 6**b**.

	**Iodine-compound 1**	**Iodoimidazole-compound TDA⁺ 6a**	**Iodoimidazole-compound NBu₄⁺ 6b**
Empirical formula	$C_{18}H_{18}I_1N_7$	$C_{147}H_{140}I_7N_{63}$	$C_{29}H_{40}Cl_2I_1N_7$
Formular weight [g/mol]	459.29	3677.51	684.48
Crystal system	triclinic	monoclinic	triclinic
Space group (Nr.)	P -1 (2)	C 1 2 1 (5)	P-1 (2)
Lattice parameters			
a [Å]	7.6844(7)	39.1532(6)	11.5154(6)
b [Å]	9.6564(8)	25.5343(3)	12.6226(7)
c [Å]	15.2980(10)	8.04170(10)	13.5713(8)
α [°]	74.738(6)	90	72.297(5)
β [°]	77.698(7)	95.5330(10)	72.226(5)
γ [°]	69.145(8)	90	64.433(5)
Cell volume [Å^3]	1014.38(15)	8002.23(18)	1660.10(19)
Z	2	2	2
Density [g/cm^3]	1.504	1.526	1.369
Diffraction Device	XtraLAB Mini	XtaLAB Synergy	XtraLAB Mini
Radiation type	0.7107 Å Mo-Kα	1.54184 Å Cu-Kα	0.71073 Å Mo-Kα
Temperature [K]	170	170	170
Absorption coefficient µ [mm^{-1}]	1.594	11.225	1.154
Absorption correction	semi-empirical	multi-scan	semi-empirical
F(000)	456	3668	700
Measured range	2.3-26.4	3.5-66.5	2.3 - 26.5
	-9<h<9	-46<h<46	-13 < h < 14
Index-range	-11<k<12	-30<k<30	-15 < k < 15
	-19<l<19	-9<l<8	-17 < l < 13
Measured reflections	7422	48528	11692
independent	4143	13955	6867
observed	3769	13637	5931
Completeness [%] / theta [°]	99.8 / 25.242	99.7 / 66.500	99.6 / 25.242
Transmission (min / max)	0.77652 / 1.00000	0.39796 / 1.00000	0.68915 / 1.00000
R_{int}	0.0374	0.045	0.0186
Structure solving/ refinement		SHELXT / SHELXL-2018/3	
R_1 (observed / all)	0.03985 / 0.0440	0.029/0.029	0.0350 / 0.0431
wR_2 (observed / all)	0.1097 / 0.1168	0.075/0.076	0.0857 / 0.0910
GooF = S	1.044	1.036	1.083
Residual electron density max/min [e Å^3]	1.514 / -1.514	0.610/-0.589	1.119 / -0.314
CCDC number	1987092	1987097	1987094

Table S2: Crystal data and structure refinement for the co-crystals of **1** with TDAI and TDACl. In all crystals one molecule of DCM is incorporated.

	**1···I⁻**	**1···I⁻···1**	**1···Cl⁻/I⁻···1**
Empirical formula	$C_{28}H_{38}Cl_2I_2N_{10}$	$C_{46}H_{56}C_{12}Cl_2I_3N_{17}$	$C_{46}H_{56}C_{12.81}I_{2.19}N_{17}$
Formular weight [g/mol]	839.38	1298.67	1224.72
Crystal system	orthorhombic	monoclinic	triclinic
Space group (Nr.)	P 2_1 2_1 2_1 (19)	P 2_1 (4)	P 1 (1)
Lattice parameters			
a [Å]	7.2319(3)	8:89139(6)	7.12096(7)
b [Å]	9.0069(4)	7.36890(5)	8.97898(8)
c [Å]	55.966(3)	43.5472(3)	22.46842(17)
α [°]	90	90	100.7911(7)
β [°]	90	91.4367(5)	97.2023(7)
γ [°]	90	90	91.5248(8)
Cell volume [Å^3]	3645.4(§9	2852.31(3)	1398.22(2)
Z	4	2	1
Density [g/cm^3]	1:529	1.512	1.454
Diffraction Device	XtaLAB Synergy	XtaLAB Synergy	XtaLAB Synergy
Radiation type	1.54184 Å Cu-Kα	1.54184 Å Cu-Kα	1.54184 Å Cu-Kα
Temperature [K]	170	170	170
Absorption coefficient μ [mm^{-1}]	15.159	14.142	11.275
Absorption correction	gaussian	gaussian	gaussian
F(000)	1664	1288	615
Measured range	3.2 – 66.5	3.0 – 67.1	4.0 – 66.5
	-8 < h < 8	-9 < h < 10	-8 < h < 8
Index-range	-10 < k < 10	-8 < k < 8	-10 < k < 10
	-65 < l < 66	-51 < l < 52	-26 < l < 26
Measured reflections	34843	37245	44758
independent	6399	10186	9701
observed	6011	10012	9449
Completeness [%] / theta [°]	99.7 / 66.482	100.0 / 67.060	99.9 / 66.492
Transmission (min / max)	0.264 / 0.741	0.370 / 0.707	0.301 / 0.970
R_{int}	0.0765	0.0408	0.0457
Structure solving/ refinement		SHELXT / SHELXL-2018/3	
R_1 (observed / all)	0.0425 / 0.0456	0.0287 / 0.0292	0.0367 / 0.0375
wR_2 (observed / all)	0.0941 / 0.0953	0.0762 / 0.0765	0.0947 / 0.0954
GooF = S	1.097	1.088	1.026
Residual electron density max/min [e Å^3]	0.871 / -0.871	0.543 / -0.589	0.692 / -0.779
CCDC number	1987093	1987096	1987095

Table S3: Crystal data and structure refinement for the crystals of **3**.

	**H-compound 3**	**H-compound 3•NBu$_4$**
Empirical formula	$C_{18}H_{19}N_7$	$C_{25}H_{37}N_5$
Formular weight [g/mol]	333.40	407.59
Crystal system	triclinic	monoclinic
Space group (Nr.)	P -1 (2)	C 1 2/c 1 (15)
Lattice parameters		
a [Å]	8.3107(13)	20.8006(3)
b [Å]	8.4240(12)	12.42402(11)
c [Å]	13.826(3)	23.3466(3)
α [°]	74.091(15)	90
β [°]	86.874(14)	116.9864(15)
γ [°]	82.828(12)	90
Cell volume [Å^3]	923.4(3)	5376.45(12)
Z	2	8
Density [g/cm^3]	1.199	1.007
Diffraction Device	SuperNova	XtaLAB Synergy
Radiation type	1.54184 Å Cu-Kα	1.54184 Å Cu-Kα
Temperature [K]	170	170
Absorption coefficient μ [mm^{-1}]	0.615	0.467
Absorption correction	semi-emirical	gaussian
F(000)	352	1776
Measured range	5.4-66.5	4.3-66.5
	-9<h<9	-24<h<24
Index-range	-10<k<10	-14<k<14
	-15<l<16	-27<l<22
Measured reflections	13246	18650
independent	3231	4740
observed	2494	4330
Completeness [%] / theta [°]	99.7 / 66.498	99.8 / 66.497
Transmission (min / max)	0.77499 / 1.000	0.391 / 1.000
R_{int}	0.0359	0.024
Structure solving/ refinement	SHELXT / SHELXL-2018/3	
R_1 (observed / all)	0.046 / 0.0611	0.0507 / 0.0538
wR_2 (observed / all)	0.1266 / 0.1384	0.1420 / 0.1447
GooF = S	1.055	1.032
Residual electron density max/min [e Å^3]	0.661 / -0.170	0.236 / -0.174
CCDC number	1992871	1993116

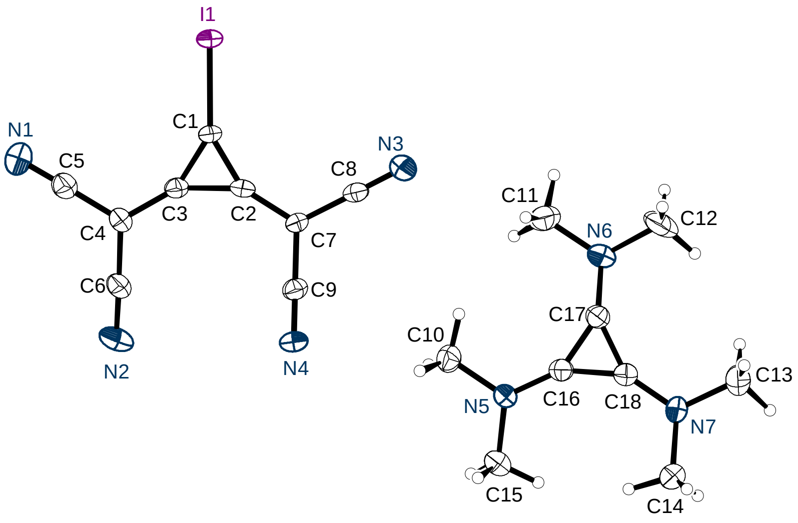

Figure S40. Crystal structure of iodine compound **1**.

Table S4. Fractional coordinates and isotropic thermal parameters of iodine-compound **1**.

Atom	x/a	y/b	z/c	U [Å²]
I1	0.10435(3)	1.20403(2)	0.64069(2)	
N1	-0.2217(7)	0.9951(5)	0.9259(3)	
C1	0.0766(6)	0.9939(4)	0.6645(2)	
N3	0.3406(5)	0.8693(4)	0.4132(2)	
C3	0.0084(5)	0.8850(4)	0.7227(2)	
N2	-0.1294(7)	0.5686(5)	0.8414(3)	
C2	0.1018(6)	0.8670(4)	0.6338(3)	
C4	-0.0845(6)	0.8302(4)	0.8055(2)	
N4	0.1366(6)	0.5117(4)	0.6055(3)	
C6	-0.1077(7)	0.6856(5)	0.8247(3)	
N6	0.5797(6)	0.6293(4)	0.1649(2)	
C5	-0.1585(7)	0.9215(5)	0.8718(3)	
N5	0.4298(5)	0.3344(4)	0.3350(2)	
C7	0.1666(5)	0.7780(4)	0.5690(2)	
N7	0.6519(6)	0.2662(4)	0.1048(2)	
C9	0.1490(5)	0.6303(4)	0.5892(3)	
C8	0.2614(6)	0.8288(4)	0.4822(3)	
C10	0.3578(6)	0.4324(5)	0.4016(3)	
H10A	0.41007	0.37850	0.45884	0.0520
H10B	0.22047	0.46050	0.41309	0.0520
H10C	0.39454	0.52409	0.37753	0.0520
C11	0.5153(9)	0.7254(6)	0.2331(4)	
H11A	0.60702	0.69085	0.27631	0.0740
H11B	0.39344	0.71878	0.26623	0.0740
H11C	0.50232	0.83061	0.20231	0.0740
C12	0.6866(8)	0.6793(6)	0.0790(4)	
H12A	0.80847	0.67658	0.09061	0.0760
H12B	0.61685	0.78298	0.05127	0.0760
H12C	0.70643	0.61199	0.03720	0.0760
C13	0.7478(7)	0.3148(6)	0.0148(3)	
H13A	0.87627	0.30583	0.02037	0.0630
H13B	0.67982	0.42051	-0.01016	0.0630
H13C	0.75156	0.25078	-0.02619	0.0630
C14	0.6458(8)	0.1126(5)	0.1309(3)	
H14A	0.57389	0.09742	0.19174	0.0630
H14B	0.77396	0.04161	0.13194	0.0630

H14C	0.58527	0.09455	0.08652	0.0630
C15	0.3971(6)	0.1888(5)	0.3622(3)	
H15A	0.26437	0.20363	0.38629	0.0530
H15B	0.47533	0.12304	0.40949	0.0530
H15C	0.42952	0.14142	0.30900	0.0530
C16	0.5080(5)	0.3802(4)	0.2517(2)	
C17	0.5695(5)	0.4911(5)	0.1878(2)	
C18	0.5946(6)	0.3551(5)	0.1655(3)	

Table S5. Anisotropic displacement parameters for the iodine-compound **1**.

Atom	U_{11}	U_{22}	U_{33}	U_{12}	U_{13}	U_{23}
I1	0.03622(19)	0.01862(17)	0.03239(17)	-0.01324(13)	-0.00341(11)	-0.00537(11)
N1	0.076(3)	0.052(3)	0.041(2)	-0.024(3)	0.008(2)	-0.020(2)
C1	0.037(2)	0.0177(17)	0.0263(17)	-0.0127(16)	-0.0027(14)	-0.0052(13)
N3	0.039(2)	0.033(2)	0.0341(19)	-0.0155(17)	0.0010(15)	-0.0055(14)
C3	0.0269(19)	0.0222(18)	0.0284(18)	-0.0104(16)	-0.0037(14)	-0.0061(14)
N2	0.066(3)	0.040(2)	0.052(2)	-0.034(2)	0.000(2)	0.0005(18)
C2	0.0271(19)	0.0174(18)	0.0289(18)	-0.0106(16)	-0.0049(14)	-0.0009(13)
C4	0.032(2)	0.029(2)	0.0239(17)	-0.0124(18)	-0.0012(14)	-0.0022(14)
N4	0.062(3)	0.026(2)	0.041(2)	-0.0232(19)	0.0039(18)	-0.0108(15)
C6	0.047(3)	0.033(2)	0.0250(19)	-0.019(2)	-0.0029(16)	-0.0005(16)
N6	0.059(2)	0.0301(19)	0.0348(19)	-0.0224(19)	0.0003(16)	-0.0025(14)
C5	0.047(3)	0.035(2)	0.028(2)	-0.018(2)	-0.0053(17)	-0.0022(17)
N5	0.0399(19)	0.0289(18)	0.0248(15)	-0.0170(16)	0.0015(13)	-0.0054(13)
C7	0.034(2)	0.0190(18)	0.0258(17)	-0.0095(16)	-0.0023(14)	-0.0068(13)
N7	0.057(2)	0.0350(19)	0.0239(16)	-0.0190(18)	0.0053(15)	-0.0110(13)
C9	0.028(2)	0.025(2)	0.0327(19)	-0.0103(17)	-0.0027(14)	-0.0093(15)
C8	0.032(2)	0.0189(18)	0.032(2)	-0.0093(17)	-0.0057(15)	-0.0064(14)
C10	0.035(2)	0.034(2)	0.032(2)	-0.0079(19)	0.0005(16)	-0.0102(17)
C11	0.078(4)	0.031(3)	0.044(3)	-0.020(3)	-0.013(2)	-0.007(2)
C12	0.056(3)	0.041(3)	0.054(3)	-0.029(3)	0.003(2)	0.006(2)
C13	0.048(3)	0.049(3)	0.032(2)	-0.020(2)	0.0029(18)	-0.0120(19)
C14	0.061(3)	0.034(2)	0.033(2)	-0.019(2)	0.0004(19)	-0.0104(18)
C15	0.040(2)	0.033(2)	0.032(2)	-0.016(2)	-0.0022(16)	-0.0012(16)
C16	0.0293(19)	0.0239(19)	0.0278(18)	-0.0095(17)	-0.0053(14)	-0.0042(14)
C17	0.031(2)	0.029(2)	0.0266(18)	-0.0124(17)	-0.0037(14)	-0.0039(14)
C18	0.037(2)	0.029(2)	0.0284(19)	-0.0159(19)	-0.0010(15)	-0.0067(15)

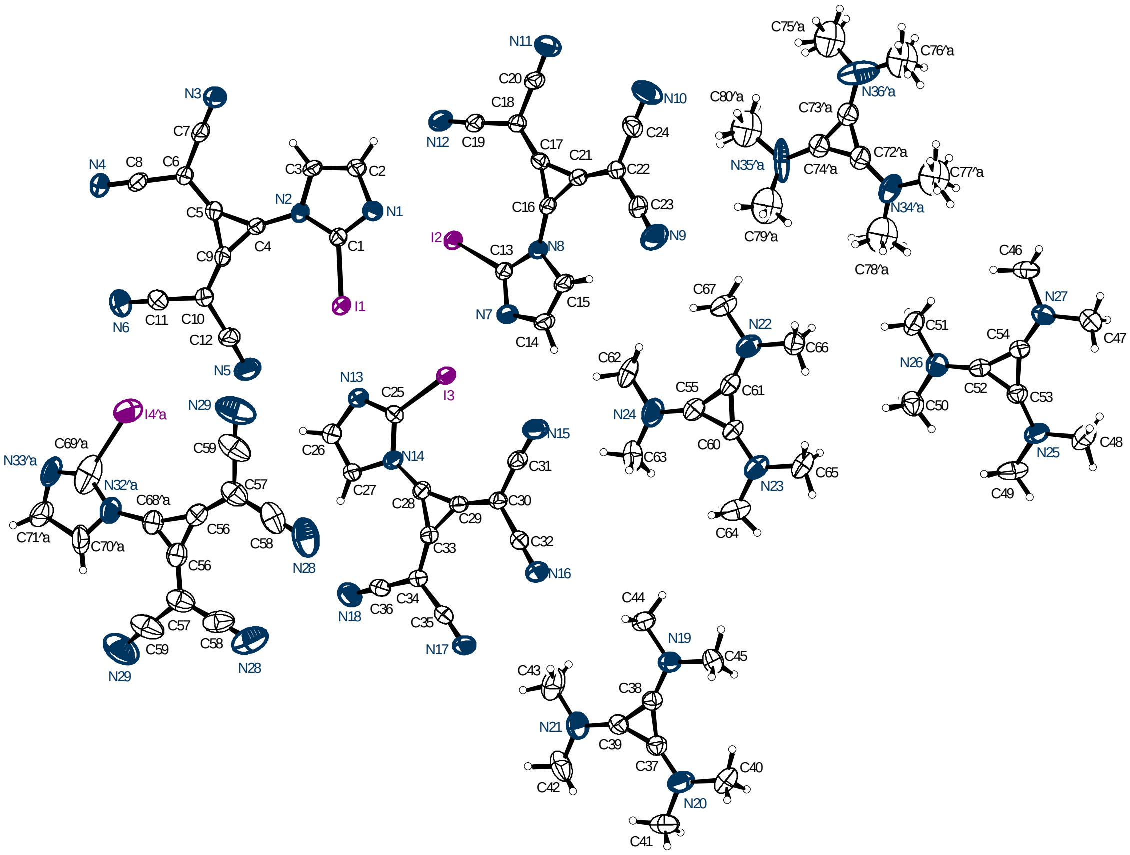

Figure S41. Crystal structure of iodoimdazole compound **6a**. The second, disordered iodoimdazole moiety of the XB donor which is not involved in the formation of a XB, is omitted for clarity. The iodine atom of the omitted ring points in the opposite direction, whereas the position of the ligand L$^-$ does not change. The atoms of the omitted fragment are named I4^b, C69^b, …

Table S6. Fractional coordinates and isotropic thermal parameters of tris(dimethylamino)cyclopropenylium 1,2-bis(dicyanomethylene)-3-(2-iodoimidazolyl)-cyclopropanid **6a**.

Atom	S.O.F.	x/a	y/b	z/c	U [Å²]
I1		0.21615(2)	0.34389(2)	0.49215(4)	
N1		0.28711(13)	0.32006(18)	0.3965(6)	
N2		0.25686(12)	0.24687(16)	0.3989(6)	
N3		0.28802(15)	0.1005(2)	0.1829(8)	
N4		0.18817(15)	0.0340(2)	0.2792(8)	
N5		0.14537(19)	0.2761(2)	0.6620(9)	
N6		0.1314(2)	0.1132(3)	0.5260(11)	
C1		0.25719(14)	0.3003(2)	0.4242(7)	
C2		0.30691(16)	0.2784(2)	0.3514(9)	
H2		0.32991	0.28111	0.32406	0.0490
C3		0.28902(15)	0.2337(2)	0.3518(7)	
H3		0.29676	0.19975	0.32521	0.0430
C4		0.23090(14)	0.2102(2)	0.4106(7)	
C5		0.22224(14)	0.1599(2)	0.3722(7)	
C6		0.23005(15)	0.1122(2)	0.3024(7)	
C7		0.26185(16)	0.1051(2)	0.2360(8)	
C8		0.20655(17)	0.0696(2)	0.2917(8)	
C9		0.19979(15)	0.1926(2)	0.4538(7)	
C10		0.16893(16)	0.1951(2)	0.5228(8)	
C11		0.14839(19)	0.1495(3)	0.5233(10)	
C12		0.15636(17)	0.2410(2)	0.5986(8)	

I2		0.29488(2)	0.42956(2)	0.31902(4)	
N7		0.26547(13)	0.53786(18)	0.2933(6)	
N8		0.31414(12)	0.53645(17)	0.1720(6)	
N9		0.3553(2)	0.6697(2)	-0.0723(11)	
N10		0.44473(18)	0.5713(3)	-0.1581(10)	
N11		0.44098(14)	0.4298(3)	-0.0734(7)	
N12		0.36086(17)	0.3505(2)	0.1515(8)	
C13		0.29119(14)	0.5082(2)	0.2591(6)	
C14		0.27137(17)	0.5867(2)	0.2287(8)	
H14		0.25664	0.61598	0.23595	0.0480
C15		0.30086(16)	0.5875(2)	0.1534(8)	
H15		0.31049	0.61632	0.09969	0.0490
C16		0.34430(15)	0.52162(19)	0.1129(7)	
C17		0.36890(14)	0.4869(2)	0.0736(7)	
C18		0.38440(14)	0.4389(2)	0.0616(6)	
C19		0.37069(16)	0.3905(2)	0.1133(8)	
C20		0.41582(14)	0.4343(2)	-0.0137(7)	
C21		0.37084(14)	0.5408(2)	0.0328(7)	
C22		0.38688(15)	0.5815(2)	-0.0404(7)	
C23		0.36990(19)	0.6309(2)	-0.0586(9)	
C24		0.41862(17)	0.5750(3)	-0.1089(8)	
I3		0.20805(2)	0.50335(2)	0.44368(4)	
N13		0.16529(12)	0.41716(18)	0.5741(6)	
N14		0.13823(11)	0.49320(17)	0.5933(5)	
N15		0.20017(16)	0.6436(2)	0.3144(8)	
N16		0.11367(15)	0.7280(2)	0.4727(8)	
N17		0.04900(15)	0.6818(2)	0.7110(9)	
N18		0.04555(15)	0.5169(2)	0.8570(8)	
C25		0.16702(13)	0.4679(2)	0.5459(7)	
C26		0.13421(17)	0.4086(2)	0.6404(9)	
H26		0.12600	0.37532	0.67093	0.0490
C27		0.11735(15)	0.4542(2)	0.6553(7)	
H27		0.09583	0.45904	0.69859	0.0430
C28		0.12877(14)	0.5444(2)	0.5821(6)	
C29		0.13178(13)	0.5959(2)	0.5372(6)	
C30		0.14592(14)	0.6400(2)	0.4679(7)	
C31		0.17598(16)	0.6401(2)	0.3839(7)	
C32		0.12790(14)	0.6887(2)	0.4713(7)	
C33		0.10416(14)	0.5786(2)	0.6253(6)	
C34		0.07493(14)	0.5898(2)	0.7017(7)	
C35		0.06089(15)	0.6407(2)	0.7046(8)	
C36		0.05835(15)	0.5497(2)	0.7860(8)	
I4^a	0.5	-0.05527(3)	0.20455(6)	0.58083(15)	
N32^a	0.5	0.0124(3)	0.2445(4)	0.4719(14)	
N33^a	0.5	0.0100(4)	0.1576(4)	0.471(2)	
C68^a	0.5	0.0042(5)	0.2971(4)	0.483(2)	
C69^a	0.5	0.001(2)	0.2001(6)	0.498(6)	
C70^a	0.5	0.0408(5)	0.2289(7)	0.417(2)	
H70^a	0.5	0.05875	0.25020	0.38288	0.0810
C71^a	0.5	0.0395(5)	0.1716(6)	0.420(2)	
H71^a	0.5	0.05698	0.14856	0.38967	0.0780
C72^a	0.5	0.0040(5)	0.3185(4)	-0.012(3)	
C73^a	0.5	-0.0178(4)	0.2804(5)	0.033(2)	
C74^a	0.5	0.0110(4)	0.2662(5)	-0.035(2)	
C75^a	0.5	-0.0576(8)	0.2156(10)	0.092(3)	
H75A^a	0.25	-0.07834	0.21181	0.15040	0.1410
H75B^a	0.25	-0.06292	0.20655	-0.02605	0.1410
H75C^a	0.25	-0.03973	0.19207	0.14251	0.1410
H75D^a	0.25	-0.04232	0.19515	0.02751	0.1410
H75E^a	0.25	-0.05774	0.20040	0.20396	0.1410
H75F^a	0.25	-0.08093	0.21488	0.03539	0.1410

C76^a	0.5	-0.0691(7)	0.3094(9)	0.154(3)	
H76A^a	0.25	-0.08794	0.29272	0.20631	0.1410
H76B^a	0.25	-0.05685	0.33345	0.23434	0.1410
H76C^a	0.25	-0.07833	0.32894	0.05524	0.1410
H76D^a	0.25	-0.06081	0.34402	0.12428	0.1410
H76E^a	0.25	-0.09189	0.30329	0.09625	0.1410
H76F^a	0.25	-0.07042	0.30780	0.27536	0.1410
C77^a	0.5	-0.0102(7)	0.4104(9)	0.022(3)	
H77A^a	0.25	0.00002	0.44447	0.00042	0.1410
H77B^a	0.25	-0.03280	0.40769	-0.04100	0.1410
H77C^a	0.25	-0.01272	0.40719	0.14202	0.1410
H77D^a	0.25	-0.03035	0.39510	0.06721	0.1410
H77E^a	0.25	0.00246	0.43187	0.10862	0.1410
H77F^a	0.25	-0.01761	0.43237	-0.07440	0.1410
C78^a	0.5	0.0420(7)	0.3849(10)	-0.106(3)	
H78A^a	0.25	0.04346	0.42323	-0.10684	0.1410
H78B^a	0.25	0.06252	0.37042	-0.04361	0.1410
H78C^a	0.25	0.04037	0.37190	-0.22151	0.1410
H78D^a	0.25	0.05411	0.35381	-0.14114	0.1410
H78E^a	0.25	0.03505	0.40662	-0.20436	0.1410
H78F^a	0.25	0.05720	0.40513	-0.02646	0.1410
C79^a	0.5	0.0673(7)	0.2453(10)	-0.126(3)	
H79A^a	0.25	0.07824	0.21388	-0.16694	0.1410
H79B^a	0.25	0.06671	0.27275	-0.21151	0.1410
H79C^a	0.25	0.08044	0.25762	-0.02378	0.1410
H79D^a	0.25	0.07202	0.28228	-0.10122	0.1410
H79E^a	0.25	0.08355	0.22341	-0.05664	0.1410
H79F^a	0.25	0.06982	0.23854	-0.24437	0.1410
C80^a	0.5	0.0266(7)	0.1768(9)	-0.073(3)	
H80A^a	0.25	0.04476	0.15708	-0.12102	0.1410
H80B^a	0.25	0.02652	0.16815	0.04523	0.1410
H80C^a	0.25	0.00431	0.16766	-0.13258	0.1410
H80D^a	0.25	0.00564	0.17151	-0.01789	0.1410
H80E^a	0.25	0.02387	0.16044	-0.18414	0.1410
H80F^a	0.25	0.04609	0.16093	-0.00634	0.1410
N34^a	0.5	0.0118(4)	0.3689(4)	-0.0283(16)	
N36^a	0.5	-0.0455(5)	0.2695(10)	0.106(2)	
N35^a	0.5	0.0327(5)	0.2328(7)	-0.091(2)	
N28		0.0417(3)	0.4719(4)	0.4283(11)	
N29		0.0888(2)	0.3298(5)	0.2510(12)	
C56		0.01531(17)	0.3420(3)	0.4627(7)	
C55		0.26199(18)	0.8165(2)	0.0568(8)	
C57		0.0411(2)	0.3716(4)	0.4026(9)	
C58		0.0409(2)	0.4260(4)	0.4158(9)	
C59		0.0674(2)	0.3478(5)	0.3208(10)	
C60		0.26404(17)	0.8701(2)	0.0602(7)	
C61		0.29014(17)	0.8416(3)	0.0020(7)	
C62		0.2613(3)	0.7221(3)	0.0709(10)	
H62A		0.24351	0.69665	0.03237	0.0950
H62B		0.27277	0.71070	0.17840	0.0950
H62C		0.27808	0.72470	-0.01136	0.0950
C63		0.2116(2)	0.7767(3)	0.1533(10)	
H63A		0.19513	0.79129	0.06606	0.0950
H63B		0.21298	0.79955	0.25173	0.0950
H63C		0.20415	0.74172	0.18394	0.0950
C64		0.2176(2)	0.9174(3)	0.1698(10)	
H64A		0.19944	0.90526	0.08657	0.0890
H64B		0.21269	0.95324	0.20355	0.0890
H64C		0.21879	0.89435	0.26772	0.0890
C65		0.2689(2)	0.9642(3)	0.0714(9)	
H65A		0.27654	0.96411	-0.04107	0.0800

H65B	0.28882	0.96686	0.15439	0.0800
H65C	0.25364	0.99418	0.08300	0.0800
C66	0.33868(19)	0.8870(3)	-0.0926(9)	
H66A	0.34383	0.88697	-0.20947	0.0740
H66B	0.36014	0.88883	-0.01927	0.0740
H66C	0.32437	0.91739	-0.07242	0.0740
C67	0.3357(2)	0.7887(3)	-0.0904(10)	
H67A	0.31836	0.76610	-0.15008	0.0830
H67B	0.34412	0.77225	0.01575	0.0830
H67C	0.35482	0.79374	-0.15883	0.0830
N22	0.32043(15)	0.8392(2)	-0.0580(6)	
N23	0.25056(16)	0.9163(2)	0.0976(7)	
N24	0.24546(18)	0.7733(2)	0.0908(8)	
N19	0.60565(13)	0.4271(2)	-0.5387(6)	
N20	0.54093(16)	0.5021(2)	-0.3401(7)	
N21	0.53686(17)	0.3580(2)	-0.3472(8)	
C37	0.55321(16)	0.4567(2)	-0.3846(7)	
C38	0.57728(15)	0.4282(2)	-0.4607(7)	
C39	0.55176(16)	0.4026(2)	-0.3861(8)	
C40	0.5575(2)	0.5511(3)	-0.3822(11)	
H40A	0.54096	0.57298	-0.45000	0.0910
H40B	0.57706	0.54337	-0.44539	0.0910
H40C	0.56549	0.56975	-0.27923	0.0910
C41	0.5114(2)	0.5053(4)	-0.2439(10)	
H41A	0.49444	0.52931	-0.29930	0.0940
H41B	0.51878	0.51830	-0.13144	0.0940
H41C	0.50121	0.47046	-0.23604	0.0940
C42	0.5092(2)	0.3590(4)	-0.2385(11)	
H42A	0.49582	0.39120	-0.25876	0.1030
H42B	0.51896	0.35808	-0.12156	0.1030
H42C	0.49432	0.32857	-0.26184	0.1030
C43	0.5526(3)	0.3084(3)	-0.3865(13)	
H43A	0.55484	0.30664	-0.50674	0.1100
H43B	0.53829	0.27939	-0.35423	0.1100
H43C	0.57544	0.30584	-0.32495	0.1100
C44	0.61919(18)	0.3774(3)	-0.5946(9)	
H44A	0.60703	0.36757	-0.70241	0.0700
H44B	0.61587	0.35006	-0.51204	0.0700
H44C	0.64373	0.38111	-0.60688	0.0700
C45	0.62068(18)	0.4754(3)	-0.5896(9)	
H45A	0.64205	0.46784	-0.63907	0.0710
H45B	0.62558	0.49817	-0.49203	0.0710
H45C	0.60461	0.49312	-0.67221	0.0710
N25	0.12155(15)	0.6909(2)	1.0246(7)	
N26	0.12011(15)	0.5500(2)	1.1108(7)	
N27	0.05058(14)	0.6357(2)	1.2338(8)	
C46	0.02944(19)	0.5908(3)	1.2693(11)	
H46A	0.01406	0.58218	1.16972	0.0900
H46B	0.01582	0.59942	1.36168	0.0900
H46C	0.04424	0.56076	1.30044	0.0900
C47	0.03591(19)	0.6882(3)	1.2368(12)	
H47A	0.02266	0.69539	1.12956	0.0940
H47B	0.05441	0.71399	1.25598	0.0940
H47C	0.02080	0.69050	1.32688	0.0940
C48	0.1051(2)	0.7418(3)	1.0244(11)	
H48A	0.11865	0.76720	0.96692	0.0980
H48B	0.10364	0.75331	1.13977	0.0980
H48C	0.08200	0.73944	0.96633	0.0980
C49	0.1508(2)	0.6829(3)	0.9284(9)	
H49A	0.14348	0.66317	0.82661	0.0880
H49B	0.16864	0.66316	0.99569	0.0880

H49C	0.16001	0.71691	0.89820	0.0880
C50	0.15124(19)	0.5439(3)	1.0259(9)	
H50A	0.14629	0.52179	0.92678	0.0750
H50B	0.16915	0.52734	1.10189	0.0750
H50C	0.15915	0.57831	0.99203	0.0750
C51	0.10466(19)	0.5033(3)	1.1724(9)	
H51A	0.09431	0.48263	1.07781	0.0750
H51B	0.08690	0.51315	1.24440	0.0750
H51C	0.12230	0.48233	1.23655	0.0750
C52	0.10538(15)	0.5962(2)	1.1157(7)	
C53	0.10602(15)	0.6494(2)	1.0836(7)	
C54	0.07926(15)	0.6283(2)	1.1611(7)	

Table S7. Anisotropic displacement parameters tris(dimethylamino)cyclopropenylium 1,2-bis(dicyanomethylene)-3-(2-iodoimidazolyl)-cyclopropanid **6a**.

Atom	U_{11}	U_{22}	U_{33}	U_{12}	U_{13}	U_{23}
I1	0.03231(17)	0.02342(16)	0.03633(17)	0.00175(13)	0.00515(13)	-0.00192(13)
N1	0.033(2)	0.027(2)	0.053(3)	0.0017(19)	0.012(2)	0.005(2)
N2	0.033(2)	0.0186(19)	0.043(2)	0.0000(17)	0.0088(18)	0.0029(17)
N3	0.045(3)	0.040(3)	0.064(3)	0.000(2)	0.023(3)	-0.004(2)
N4	0.048(3)	0.031(3)	0.069(4)	-0.008(2)	0.014(3)	-0.007(2)
N5	0.074(4)	0.040(3)	0.080(4)	0.005(3)	0.042(4)	-0.001(3)
N6	0.062(4)	0.052(4)	0.115(6)	-0.021(3)	0.038(4)	-0.010(4)
C1	0.030(3)	0.024(2)	0.040(3)	0.001(2)	0.002(2)	0.003(2)
C2	0.032(3)	0.028(3)	0.063(4)	0.003(2)	0.013(3)	0.004(3)
C3	0.037(3)	0.023(3)	0.050(3)	0.007(2)	0.014(2)	0.001(2)
C4	0.033(3)	0.024(3)	0.038(3)	0.003(2)	0.010(2)	0.001(2)
C5	0.033(3)	0.027(3)	0.038(3)	-0.002(2)	0.006(2)	0.003(2)
C6	0.035(3)	0.023(2)	0.045(3)	-0.001(2)	0.010(2)	0.001(2)
C7	0.045(3)	0.022(3)	0.047(3)	0.000(2)	0.010(3)	0.000(2)
C8	0.047(3)	0.025(3)	0.045(3)	0.004(3)	0.012(3)	-0.004(2)
C9	0.036(3)	0.023(3)	0.040(3)	-0.004(2)	0.004(2)	0.002(2)
C10	0.038(3)	0.030(3)	0.052(3)	-0.003(2)	0.016(2)	-0.003(2)
C11	0.046(4)	0.042(4)	0.069(4)	-0.006(3)	0.026(3)	-0.005(3)
C12	0.046(3)	0.037(3)	0.052(3)	0.003(3)	0.024(3)	0.002(3)
I2	0.03310(18)	0.02269(17)	0.03663(18)	0.00138(13)	0.00847(13)	0.00235(12)
N7	0.038(3)	0.027(2)	0.045(3)	0.0036(19)	0.017(2)	0.0032(19)
N8	0.033(2)	0.020(2)	0.040(2)	0.0015(17)	0.0140(18)	0.0037(17)
N9	0.084(5)	0.032(3)	0.108(6)	0.010(3)	0.036(4)	0.013(3)
N10	0.048(4)	0.075(5)	0.088(5)	0.009(3)	0.034(3)	0.028(4)
N11	0.038(3)	0.054(3)	0.058(3)	0.008(3)	0.013(2)	0.007(3)
N12	0.058(3)	0.031(3)	0.077(4)	0.004(3)	0.034(3)	0.002(3)
C13	0.035(3)	0.024(3)	0.037(3)	-0.001(2)	0.011(2)	-0.001(2)
C14	0.045(3)	0.025(3)	0.055(4)	0.007(2)	0.024(3)	0.005(2)
C15	0.045(3)	0.024(3)	0.056(3)	0.005(2)	0.020(3)	0.005(2)
C16	0.034(3)	0.019(2)	0.039(3)	0.001(2)	0.009(2)	0.0022(19)
C17	0.026(2)	0.032(3)	0.031(3)	0.000(2)	0.006(2)	0.003(2)
C18	0.032(3)	0.026(3)	0.035(3)	0.002(2)	0.008(2)	0.004(2)
C19	0.038(3)	0.026(3)	0.047(3)	0.003(2)	0.012(2)	0.002(2)
C20	0.034(3)	0.029(3)	0.041(3)	0.008(2)	0.004(2)	0.003(2)
C21	0.032(3)	0.026(3)	0.035(3)	0.003(2)	0.007(2)	-0.001(2)
C22	0.035(3)	0.027(3)	0.048(3)	-0.001(2)	0.015(2)	0.003(2)
C23	0.054(4)	0.030(3)	0.061(4)	-0.005(3)	0.022(3)	0.005(3)
C24	0.038(3)	0.042(3)	0.053(4)	-0.003(3)	0.010(3)	0.009(3)
I3	0.03316(17)	0.02514(16)	0.03664(18)	0.00207(13)	0.00947(13)	-0.00062(13)
N13	0.034(2)	0.027(2)	0.049(3)	0.0018(19)	0.011(2)	-0.0028(19)
N14	0.033(2)	0.025(2)	0.035(2)	0.0021(18)	0.0082(17)	-0.0036(17)
N15	0.055(4)	0.037(3)	0.065(4)	0.010(2)	0.031(3)	0.009(2)
N16	0.041(3)	0.032(3)	0.074(4)	0.006(2)	0.012(3)	-0.002(2)
N17	0.041(3)	0.033(3)	0.099(5)	0.008(2)	0.026(3)	0.000(3)
N18	0.042(3)	0.049(3)	0.065(4)	-0.002(2)	0.012(3)	0.009(3)

C25	0.027(3)	0.033(3)	0.034(3)	-0.002(2)	0.006(2)	-0.001(2)
C26	0.040(3)	0.032(3)	0.053(4)	-0.001(3)	0.011(3)	0.000(3)
C27	0.031(3)	0.028(3)	0.048(3)	0.000(2)	0.011(2)	0.002(2)
C28	0.034(3)	0.027(3)	0.031(3)	0.000(2)	0.005(2)	-0.002(2)
C29	0.027(3)	0.034(3)	0.031(3)	0.003(2)	0.003(2)	-0.006(2)
C30	0.032(3)	0.028(2)	0.034(3)	0.001(2)	0.010(2)	-0.001(2)
C31	0.044(3)	0.027(3)	0.036(3)	0.003(2)	0.010(2)	0.003(2)
C32	0.031(3)	0.034(3)	0.041(3)	-0.001(2)	0.006(2)	0.001(2)
C33	0.029(3)	0.029(3)	0.035(3)	0.001(2)	0.002(2)	-0.001(2)
C34	0.030(3)	0.030(3)	0.042(3)	0.003(2)	0.008(2)	-0.002(2)
C35	0.028(3)	0.038(3)	0.057(4)	-0.003(2)	0.013(2)	-0.002(3)
C36	0.029(3)	0.039(3)	0.049(3)	0.003(2)	0.011(2)	0.000(3)
I4^a	0.0633(7)	0.0579(7)	0.0813(7)	-0.0148(6)	0.0102(5)	0.0090(5)
N32^a	0.059(7)	0.033(6)	0.050(6)	0.007(5)	0.009(5)	-0.002(5)
N33^a	0.077(12)	0.021(5)	0.094(12)	0.004(5)	0.002(8)	-0.002(6)
C68^a	0.051(3)	0.038(2)	0.042(3)	0.003(3)	0.001(2)	-0.005(2)
C69^a	0.154(16)	0.044(8)	0.051(8)	-0.01(3)	0.001(10)	0.031(17)
C70^a	0.074(11)	0.046(9)	0.087(12)	0.030(9)	0.022(9)	0.007(8)
C71^a	0.082(12)	0.027(7)	0.088(12)	-0.006(8)	0.024(9)	-0.012(7)
C72^a	0.042(5)	0.041(4)	0.046(4)	0.000(3)	-0.008(3)	0.000(4)
C73^a	0.042(5)	0.041(4)	0.046(4)	0.000(3)	-0.008(3)	0.000(4)
C74^a	0.042(5)	0.041(4)	0.046(4)	0.000(3)	-0.008(3)	0.000(4)
C75^a	0.116(8)	0.086(6)	0.079(5)	0.008(5)	0.002(5)	-0.015(4)
C76^a	0.116(8)	0.086(6)	0.079(5)	0.008(5)	0.002(5)	-0.015(4)
C77^a	0.116(8)	0.086(6)	0.079(5)	0.008(5)	0.002(5)	-0.015(4)
C78^a	0.116(8)	0.086(6)	0.079(5)	0.008(5)	0.002(5)	-0.015(4)
C79^a	0.116(8)	0.086(6)	0.079(5)	0.008(5)	0.002(5)	-0.015(4)
C80^a	0.116(8)	0.086(6)	0.079(5)	0.008(5)	0.002(5)	-0.015(4)
N34^a	0.076(10)	0.026(5)	0.065(8)	0.000(5)	0.001(6)	-0.005(5)
N36^a	0.094(14)	0.107(16)	0.082(11)	-0.058(13)	0.007(9)	0.012(11)
N35^a	0.110(14)	0.057(9)	0.076(10)	0.062(10)	-0.022(9)	-0.021(8)
N28	0.097(6)	0.085(6)	0.079(5)	-0.034(5)	-0.002(4)	0.024(4)
N29	0.075(6)	0.146(9)	0.091(6)	0.016(6)	0.042(5)	0.009(6)
C56	0.051(3)	0.038(2)	0.042(3)	0.003(3)	0.001(2)	-0.005(2)
C55	0.054(4)	0.035(3)	0.038(3)	0.003(3)	0.003(3)	0.001(2)
C57	0.048(4)	0.081(6)	0.045(4)	-0.005(4)	0.008(3)	0.002(4)
C58	0.061(5)	0.085(6)	0.046(4)	-0.023(5)	-0.001(3)	0.016(4)
C59	0.049(4)	0.123(8)	0.053(4)	-0.005(5)	0.012(3)	0.007(5)
C60	0.051(4)	0.027(3)	0.034(3)	0.004(2)	0.007(2)	-0.001(2)
C61	0.054(4)	0.027(3)	0.033(3)	0.003(3)	0.002(2)	0.001(2)
C62	0.097(6)	0.027(3)	0.065(5)	-0.008(4)	0.004(4)	0.004(3)
C63	0.076(5)	0.055(5)	0.060(5)	-0.027(4)	0.006(4)	0.002(3)
C64	0.070(5)	0.057(5)	0.054(4)	0.023(4)	0.015(3)	0.002(3)
C65	0.078(5)	0.030(3)	0.050(4)	0.001(3)	0.002(3)	-0.002(3)
C66	0.054(4)	0.041(3)	0.054(4)	-0.006(3)	0.010(3)	-0.002(3)
C67	0.070(5)	0.041(4)	0.057(4)	0.017(3)	0.014(3)	0.000(3)
N22	0.058(3)	0.035(3)	0.043(3)	0.009(2)	0.012(2)	0.003(2)
N23	0.064(3)	0.032(3)	0.044(3)	0.007(2)	0.010(2)	-0.001(2)
N24	0.074(4)	0.031(3)	0.067(4)	-0.008(3)	0.014(3)	0.007(2)
N19	0.039(3)	0.038(2)	0.046(3)	0.003(2)	0.013(2)	-0.002(2)
N20	0.055(3)	0.044(3)	0.056(3)	0.012(3)	0.014(2)	-0.004(3)
N21	0.059(4)	0.043(3)	0.067(4)	-0.011(3)	0.017(3)	0.007(3)
C37	0.037(3)	0.038(3)	0.040(3)	0.002(2)	0.003(2)	-0.001(2)
C38	0.037(3)	0.034(3)	0.035(3)	0.003(2)	0.004(2)	0.003(2)
C39	0.037(3)	0.038(3)	0.039(3)	-0.001(2)	0.007(2)	0.001(2)
C40	0.077(6)	0.036(4)	0.069(5)	0.003(3)	0.005(4)	-0.010(3)
C41	0.050(4)	0.075(5)	0.065(4)	0.019(4)	0.012(3)	-0.013(4)
C42	0.065(5)	0.080(6)	0.064(5)	-0.034(4)	0.019(4)	0.002(4)
C43	0.093(7)	0.038(4)	0.086(6)	-0.003(4)	-0.007(5)	0.012(4)
C44	0.047(4)	0.043(3)	0.052(4)	0.011(3)	0.008(3)	-0.007(3)
C45	0.046(4)	0.050(4)	0.047(4)	-0.009(3)	0.008(3)	0.007(3)

N25	0.049(3)	0.038(3)	0.054(3)	-0.010(2)	0.013(2)	0.007(2)
N26	0.046(3)	0.037(3)	0.057(3)	0.001(2)	0.008(2)	-0.003(2)
N27	0.034(3)	0.042(3)	0.083(4)	-0.003(2)	0.018(3)	0.000(3)
C46	0.043(4)	0.057(4)	0.082(5)	-0.016(3)	0.022(3)	0.002(4)
C47	0.042(4)	0.053(4)	0.095(6)	0.004(3)	0.015(4)	0.001(4)
C48	0.079(6)	0.039(4)	0.077(5)	-0.007(4)	0.002(4)	0.010(3)
C49	0.064(5)	0.065(5)	0.049(4)	-0.027(4)	0.022(3)	-0.003(3)
C50	0.053(4)	0.052(4)	0.046(3)	0.008(3)	0.010(3)	-0.002(3)
C51	0.058(4)	0.032(3)	0.059(4)	0.000(3)	0.000(3)	0.004(3)
C52	0.038(3)	0.037(3)	0.037(3)	-0.007(2)	0.005(2)	-0.002(2)
C53	0.036(3)	0.036(3)	0.039(3)	-0.003(2)	0.003(2)	0.000(2)
C54	0.034(3)	0.034(3)	0.046(3)	-0.006(2)	0.004(2)	-0.001(2)

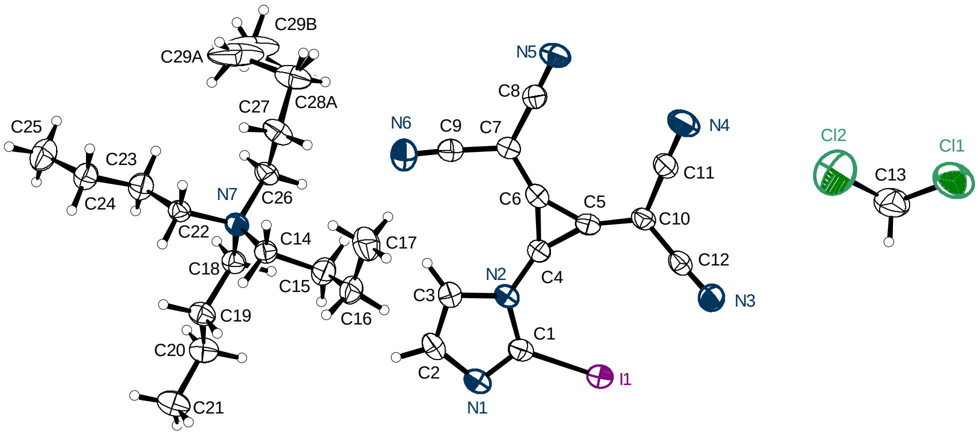

Figure 42. Crystal structure of XB donor **6b**.

Table S8. Fractional coordinates and isotropic thermal parameters tetrabutylammonium 1,2-bis(dicyanomethylene)-3-(2-iodoimidazolyl)-cyclopropanid **6b**.

Atom	S.O.F.	x/a	y/b	z/c	U [Å²]
I1		-0.10290(2)	0.74415(2)	0.48595(2)	
N1		-0.2311(2)	0.9983(2)	0.51814(19)	
N2		-0.06990(19)	0.89334(19)	0.60749(16)	
N3		0.0705(2)	0.4524(2)	0.61559(19)	
N4		0.3476(3)	0.4290(2)	0.7864(2)	
N5		0.3623(2)	0.6701(2)	0.86038(17)	
N6		0.0701(3)	1.0381(3)	0.7769(2)	
C1		-0.1408(2)	0.8926(2)	0.5424(2)	
C2		-0.2190(3)	1.0707(3)	0.5706(2)	
H2		-0.27283	1.15312	0.56833	0.0590
C3		-0.1222(3)	1.0102(2)	0.6246(2)	
H3		-0.09480	1.04040	0.66604	0.0510
C4		0.0319(2)	0.8032(2)	0.65043(19)	
C5		0.1111(2)	0.6851(2)	0.67857(19)	
C6		0.1208(2)	0.7791(2)	0.70979(19)	
C7		0.1749(2)	0.8158(2)	0.7642(2)	
C8		0.2788(2)	0.7346(2)	0.81687(19)	
C9		0.1206(3)	0.9387(3)	0.7715(2)	
C10		0.1600(2)	0.5626(2)	0.6864(2)	
C11		0.2640(3)	0.4886(2)	0.7424(2)	
C12		0.1094(2)	0.5038(2)	0.6461(2)	
Cl1		0.48988(13)	-0.10896(11)	0.68088(10)	
Cl2		0.39396(14)	0.13409(13)	0.7152(1)	
C13		0.4306(4)	0.0430(4)	0.6283(3)	
H13A		0.35014	0.06329	0.60281	0.0870

H13B		0.49681	0.06080	0.56638	0.0870
N7		0.25782(18)	0.59898(18)	0.18550(15)	
C14		0.3464(2)	0.6705(2)	0.1339(2)	
H14A		0.43821	0.61560	0.13780	0.0390
H14B		0.34173	0.70006	0.05820	0.0390
C15		0.3143(3)	0.7769(3)	0.1816(2)	
H15A		0.33348	0.74761	0.25353	0.0530
H15B		0.21941	0.82681	0.18754	0.0530
C16		0.3943(3)	0.8526(3)	0.1139(2)	
H16A		0.38721	0.90880	0.15489	0.0570
H16B		0.48793	0.79928	0.09877	0.0570
C17		0.3521(4)	0.9242(3)	0.0101(3)	
H17A		0.40224	0.97610	-0.02587	0.0970
H17B		0.25807	0.97359	0.02384	0.0970
H17C		0.36872	0.86921	-0.03475	0.0970
C18		0.2591(2)	0.5521(2)	0.30276(18)	
H18A		0.22789	0.62131	0.33691	0.0400
H18B		0.19595	0.51111	0.33429	0.0400
C19		0.3916(2)	0.4665(2)	0.3277(2)	
H19A		0.45654	0.50539	0.29443	0.0450
H19B		0.42145	0.39438	0.29783	0.0450
C20		0.3848(3)	0.4293(3)	0.4463(2)	
H20A		0.35214	0.50194	0.47610	0.0510
H20B		0.32123	0.38888	0.47896	0.0510
C21		0.5172(3)	0.3458(3)	0.4741(2)	
H21A		0.54713	0.27140	0.44908	0.0770
H21B		0.50879	0.32757	0.55092	0.0770
H21C		0.58112	0.38461	0.44036	0.0770
C22		0.3125(2)	0.4954(2)	0.12787(18)	
H22A		0.30677	0.52943	0.05268	0.0370
H22B		0.40684	0.45203	0.13062	0.0370
C23		0.2461(3)	0.4053(3)	0.1694(2)	
H23A		0.15214	0.44602	0.16470	0.0500
H23B		0.25122	0.36935	0.24445	0.0500
C24		0.3136(3)	0.3078(2)	0.1045(2)	
H24A		0.40879	0.27219	0.10497	0.0510
H24B		0.30334	0.34404	0.03046	0.0510
C25		0.2580(3)	0.2090(3)	0.1465(3)	
H25A		0.16325	0.24412	0.14847	0.0850
H25B		0.27394	0.16873	0.21797	0.0850
H25C		0.30116	0.15068	0.10015	0.0850
C26		0.1153(2)	0.6758(2)	0.1770(2)	
H26A		0.06315	0.62412	0.20710	0.0430
H26B		0.08126	0.73690	0.22097	0.0430
C27		0.0928(3)	0.7391(3)	0.0665(2)	
H27A		0.14633	0.68300	0.01773	0.0540
H27B		0.12237	0.80736	0.04342	0.0540
C28A	0.27(2)	-0.0502(3)	0.7849(3)	0.0602(2)	
H28A	0.27(2)	-0.07164	0.85163	-0.00105	0.0690
H28B	0.27(2)	-0.11004	0.81025	0.12585	0.0690
C29A	0.27(2)	-0.052(3)	0.6696(19)	0.046(2)	
H29A	0.27(2)	-0.01091	0.65909	-0.02712	0.1580
H29B	0.27(2)	-0.14342	0.67552	0.06080	0.1580
H29C	0.27(2)	-0.00376	0.60076	0.09440	0.1580
C28B	0.73(2)	-0.0502(3)	0.7849(3)	0.0602(2)	
H28C	0.73(2)	-0.10213	0.83953	0.11048	0.0690
H28D	0.73(2)	-0.05960	0.83351	-0.01160	0.0690
C29B	0.73(2)	-0.1105(14)	0.6941(10)	0.0821(9)	
H29D	0.73(2)	-0.19883	0.73416	0.06618	0.1580
H29E	0.73(2)	-0.11638	0.65432	0.15675	0.1580
H29F	0.73(2)	-0.05594	0.63438	0.03786	0.1580

Table S9. Anisotropic displacement parameters of tetrabutylammonium 1,2-bis(dicyanomethylene)-3-(2-iodoimidazolyl)-cyclopropanid **6b**

Atom	U_{11}	U_{22}	U_{33}	U_{12}	U_{13}	U_{23}
I1	0.03923(11)	0.03926(11)	0.04544(12)	-0.01117(8)	-0.01697(7)	-0.00770(8)
N1	0.0399(13)	0.0395(13)	0.0569(15)	-0.0043(10)	-0.0224(11)	-0.0094(11)
N2	0.0309(11)	0.0319(11)	0.0403(11)	-0.0061(9)	-0.0134(8)	-0.0059(9)
N3	0.0398(12)	0.0387(13)	0.0523(14)	-0.0167(10)	-0.0097(10)	-0.0085(11)
N4	0.0473(15)	0.0435(15)	0.092(2)	-0.0187(12)	-0.0359(14)	0.0102(14)
N5	0.0327(12)	0.0548(15)	0.0385(12)	-0.0059(10)	-0.0115(9)	-0.0116(11)
N6	0.079(2)	0.0423(16)	0.083(2)	-0.0068(14)	-0.0363(16)	-0.0221(15)
C1	0.0321(13)	0.0384(14)	0.0400(14)	-0.0106(11)	-0.0119(10)	-0.0052(11)
C2	0.0433(16)	0.0331(15)	0.0620(18)	0.0018(12)	-0.0208(13)	-0.0119(13)
C3	0.0429(15)	0.0351(14)	0.0489(16)	-0.0085(12)	-0.0146(12)	-0.0113(12)
C4	0.0322(12)	0.0317(13)	0.0367(13)	-0.0102(10)	-0.010(1)	-0.0055(10)
C5	0.0273(12)	0.0374(14)	0.0333(12)	-0.0118(10)	-0.0072(9)	-0.0048(10)
C6	0.0301(12)	0.0307(13)	0.0345(13)	-0.0076(10)	-0.0072(9)	-0.0035(10)
C7	0.0324(13)	0.0336(13)	0.0386(13)	-0.0088(10)	-0.0131(10)	-0.0059(11)
C8	0.0320(13)	0.0392(14)	0.0329(12)	-0.0121(11)	-0.0036(10)	-0.0113(11)
C9	0.0435(15)	0.0428(16)	0.0480(16)	-0.0093(12)	-0.0209(12)	-0.0120(13)
C10	0.0278(12)	0.0323(13)	0.0422(14)	-0.0106(10)	-0.0097(10)	-0.0025(11)
C11	0.0330(14)	0.0309(14)	0.0634(18)	-0.0144(11)	-0.0153(12)	-0.0003(13)
C12	0.0273(12)	0.0310(13)	0.0420(14)	-0.0084(10)	-0.006(1)	-0.0030(11)
Cl1	0.1017(8)	0.0732(7)	0.1108(9)	-0.0388(6)	-0.0546(7)	0.0222(6)
Cl2	0.1151(10)	0.1124(10)	0.0816(7)	-0.0525(8)	-0.0035(6)	-0.0267(7)
C13	0.079(3)	0.071(3)	0.070(2)	-0.025(2)	-0.0365(19)	0.0017(19)
N7	0.0231(9)	0.0306(11)	0.0336(10)	-0.0079(8)	-0.0049(7)	-0.0078(8)
C14	0.0284(12)	0.0315(13)	0.0386(13)	-0.0117(10)	-0.0058(9)	-0.007(1)
C15	0.0454(15)	0.0411(15)	0.0495(16)	-0.0171(12)	-0.0071(12)	-0.0156(13)
C16	0.0488(16)	0.0364(15)	0.0632(18)	-0.0187(13)	-0.0136(13)	-0.0116(13)
C17	0.074(2)	0.0383(17)	0.075(2)	-0.0181(16)	-0.0237(18)	0.0017(16)
C18	0.0285(12)	0.0368(14)	0.0319(12)	-0.0098(10)	-0.0036(9)	-0.0094(11)
C19	0.0306(13)	0.0434(15)	0.0346(13)	-0.0098(11)	-0.0062(10)	-0.0075(11)
C20	0.0389(14)	0.0522(17)	0.0349(13)	-0.0179(12)	-0.0065(11)	-0.0064(12)
C21	0.0451(16)	0.061(2)	0.0423(15)	-0.0163(14)	-0.0158(12)	0.0001(14)
C22	0.0275(11)	0.0321(13)	0.0322(12)	-0.0085(9)	-0.0057(9)	-0.0081(10)
C23	0.0375(14)	0.0421(15)	0.0456(15)	-0.0172(12)	-0.0023(11)	-0.0131(12)
C24	0.0454(15)	0.0390(15)	0.0456(15)	-0.0179(12)	-0.0088(12)	-0.0104(12)
C25	0.071(2)	0.0485(18)	0.0605(19)	-0.0311(16)	-0.0124(16)	-0.0123(15)
C26	0.0224(11)	0.0377(14)	0.0433(14)	-0.0042(10)	-0.0056(9)	-0.0126(11)
C27	0.0305(13)	0.0451(16)	0.0459(16)	-0.0085(12)	-0.0094(11)	0.0023(12)
C28A	0.0354(15)	0.080(2)	0.0506(17)	-0.0122(15)	-0.0152(13)	-0.0098(17)
C29A	0.100(8)	0.157(6)	0.092(7)	-0.101(7)	-0.065(6)	0.056(5)
C28B	0.0354(15)	0.080(2)	0.0506(17)	-0.0122(15)	-0.0152(13)	-0.0098(17)
C29B	0.100(8)	0.157(6)	0.092(7)	-0.101(7)	-0.065(6)	0.056(5)

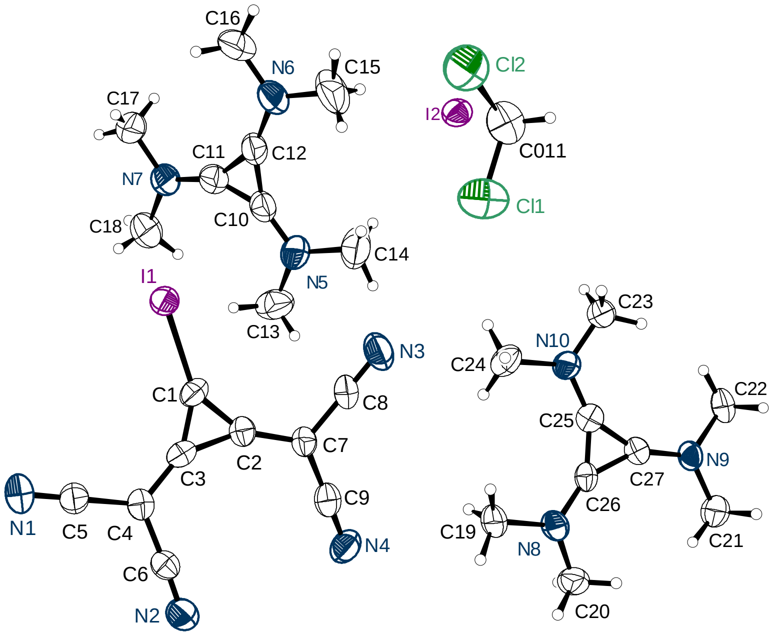

Figure S43. Crystal structure of the co-crystal between **1** and one equivalent of iodide.

Table S10. Fractional Coordinates and Isotropic Thermal Parameters of the crystal structure of the co-crystal **1**···I^-.

Atom	x/a	y/b	z/c	U [Å²]
I1	0.38919(8)	0.95334(6)	0.35575(2)	
N1	0.2007(14)	1.3292(10)	0.41974(18)	
C1	0.4054(13)	0.9206(10)	0.39279(14)	
Cl1	0.8672(7)	0.2535(5)	0.33486(6)	
I2	0.37781(9)	-0.00596(7)	0.29661(2)	
N2	0.3619(13)	1.0268(9)	0.47751(14)	
C2	0.4570(12)	0.8296(10)	0.41099(16)	
Cl2	0.9503(5)	0.3022(4)	0.28464(7)	
C5	0.2599(13)	1.2169(11)	0.42518(17)	
N5	0.2468(13)	0.506(1)	0.34612(16)	
C4	0.3277(11)	1.0756(9)	0.43224(16)	
N4	0.5495(12)	0.635(1)	0.46367(16)	
C3	0.3823(11)	0.9701(9)	0.41561(13)	
N3	0.6817(13)	0.5233(10)	0.38802(17)	
N6	0.3404(13)	0.5463(11)	0.28186(16)	
C6	0.3496(12)	1.0459(10)	0.45719(16)	
N7	-0.0700(14)	0.7167(11)	0.30766(17)	
C7	0.5360(12)	0.700(1)	0.41902(16)	
C8	0.6141(15)	0.5984(9)	0.40203(17)	
N8	0.8343(11)	0.1520(8)	0.47076(14)	
C9	0.5446(12)	0.6629(11)	0.44372(19)	
N9	0.9966(11)	-0.2190(8)	0.45350(14)	
N10	0.8369(11)	0.0437(9)	0.40758(13)	
C10	0.2014(14)	0.5603(11)	0.32505(19)	
C011	0.896(2)	0.1706(13)	0.3067(2)	
H01A	0.78053	0.11847	0.30225	0.0880
H01B	0.99617	0.09599	0.30764	0.0880
C11	0.0849(16)	0.6402(12)	0.31062(18)	
C12	0.2387(15)	0.5764(12)	0.3011(2)	
C13	0.139(2)	0.5410(14)	0.36697(19)	
H13A	0.09342	0.64330	0.36580	0.1120
H13B	0.21566	0.53071	0.38129	0.1120

H13C	0.03336	0.47284	0.36802	0.1120
C14	0.387(3)	0.3909(14)	0.3479(3)	
H14A	0.34626	0.30309	0.33896	0.1410
H14B	0.40545	0.36455	0.36468	0.1410
H14C	0.50326	0.42758	0.34112	0.1410
C16	0.287(2)	0.6036(16)	0.2588(2)	
H16A	0.38660	0.58597	0.24724	0.1260
H16B	0.26346	0.71048	0.26001	0.1260
H16C	0.17430	0.55321	0.25335	0.1260
C17	-0.116(2)	0.7740(13)	0.28375(19)	
H17A	-0.02237	0.84622	0.27879	0.1070
H17B	-0.23754	0.82219	0.28424	0.1070
H17C	-0.11919	0.69159	0.27232	0.1070
C18	-0.189(2)	0.7490(17)	0.3278(3)	
H18A	-0.31862	0.74635	0.32258	0.1320
H18B	-0.16015	0.84783	0.33406	0.1320
H18C	-0.16972	0.67462	0.34031	0.1320
C19	0.7630(16)	0.2976(10)	0.46394(19)	
H19A	0.82487	0.33108	0.44933	0.0780
H19B	0.78654	0.36872	0.47683	0.0780
H19C	0.62954	0.29060	0.46108	0.0780
C20	0.8260(16)	0.1119(11)	0.49587(17)	
H20A	0.70011	0.08033	0.49990	0.0830
H20B	0.85987	0.19797	0.50564	0.0830
H20C	0.91236	0.03037	0.49900	0.0830
C21	1.0251(16)	-0.2587(11)	0.47843(19)	
H21A	1.14526	-0.30779	0.48020	0.0820
H21B	0.92676	-0.32630	0.48361	0.0820
H21C	1.02247	-0.16874	0.48828	0.0820
C22	1.0280(13)	-0.3346(10)	0.43576(19)	
H22A	1.01564	-0.29234	0.41970	0.0710
H22B	0.93687	-0.41398	0.43789	0.0710
H22C	1.15282	-0.37521	0.43775	0.0710
C23	0.8963(15)	-0.0540(12)	0.38873(16)	
H23A	0.78903	-0.08447	0.37922	0.0790
H23B	0.95516	-0.14200	0.39568	0.0790
H23C	0.98505	-0.00215	0.37847	0.0790
C24	0.7680(15)	0.1915(11)	0.40134(19)	
H24A	0.69147	0.18493	0.38693	0.0790
H24B	0.87282	0.25800	0.39834	0.0790
H24C	0.69376	0.23067	0.41456	0.0790
C25	0.8680(12)	0.0122(8)	0.43051(14)	
C26	0.8631(12)	0.0480(9)	0.45401(14)	
C27	0.9253(11)	-0.0906(9)	0.44744(15)	
C15	0.5031(18)	0.4516(16)	0.2838(3)	
H15A	0.58600	0.47093	0.27028	0.1340
H15B	0.46495	0.34723	0.28357	0.1340
H15C	0.56776	0.47306	0.29877	0.1340

Table S11. Anisotropic Displacement Parameters of the crystal structure of the co-crystal **1**···I$^-$.

Atom	U_{11}	U_{22}	U_{33}	U_{12}	U_{13}	U_{23}
I1	0.0393(3)	0.0440(3)	0.0385(3)	0.0047(3)	0.0047(3)	0.0032(2)
N1	0.075(6)	0.038(5)	0.072(6)	0.012(4)	-0.001(5)	0.001(4)
C1	0.037(5)	0.049(5)	0.035(4)	0.005(4)	0.001(4)	0.011(4)
Cl1	0.091(3)	0.121(3)	0.077(2)	-0.015(3)	0.005(2)	-0.0104(19)
I2	0.0458(3)	0.0708(4)	0.0469(3)	0.0039(3)	0.0051(3)	0.0058(3)
N2	0.073(6)	0.044(5)	0.049(5)	0.001(5)	0.002(4)	-0.005(4)
C2	0.035(4)	0.032(5)	0.044(5)	-0.002(3)	0.003(4)	0.002(4)

Cl2	0.079(2)	0.098(3)	0.091(2)	-0.0030(18)	-0.0073(17)	-0.006(2)
C5	0.045(5)	0.040(6)	0.045(5)	0.003(4)	0.002(4)	0.001(4)
N5	0.074(6)	0.042(5)	0.061(5)	0.002(4)	-0.001(4)	0.010(4)
C4	0.031(4)	0.035(5)	0.046(5)	0.000(3)	0.005(3)	0.002(4)
N4	0.057(5)	0.064(6)	0.046(5)	0.012(4)	0.000(4)	0.014(4)
C3	0.025(3)	0.047(5)	0.031(4)	-0.003(5)	0.000(3)	0.007(3)
N3	0.071(6)	0.045(5)	0.072(6)	0.015(4)	0.007(5)	-0.007(4)
N6	0.076(6)	0.062(6)	0.065(6)	0.021(5)	0.024(5)	0.001(5)
C6	0.041(5)	0.036(5)	0.043(5)	-0.003(4)	0.003(4)	-0.004(4)
N7	0.066(7)	0.072(6)	0.058(5)	0.029(5)	0.014(5)	0.006(4)
C7	0.036(4)	0.038(5)	0.045(5)	0.007(4)	0.001(4)	0.000(4)
C8	0.045(5)	0.033(5)	0.059(5)	0.008(5)	-0.001(5)	0.001(4)
N8	0.054(5)	0.027(4)	0.049(4)	-0.001(3)	-0.001(3)	-0.003(3)
C9	0.032(4)	0.040(5)	0.059(7)	0.002(4)	0.002(4)	0.001(4)
N9	0.046(4)	0.027(4)	0.048(4)	0.005(3)	-0.002(3)	0.000(3)
N10	0.061(5)	0.036(4)	0.039(4)	0.005(4)	-0.002(3)	0.004(3)
C10	0.060(6)	0.036(6)	0.057(6)	0.005(5)	0.006(5)	-0.002(5)
C011	0.053(7)	0.072(8)	0.094(9)	-0.008(7)	0.003(7)	-0.007(6)
C11	0.062(7)	0.052(6)	0.051(6)	0.007(5)	0.011(5)	0.003(4)
C12	0.059(6)	0.048(6)	0.060(6)	0.017(5)	0.014(5)	0.007(5)
C13	0.103(9)	0.070(8)	0.051(6)	-0.016(8)	0.000(7)	0.007(5)
C14	0.122(12)	0.063(8)	0.097(10)	0.030(9)	-0.036(10)	-0.003(6)
C16	0.111(11)	0.081(9)	0.060(8)	0.017(8)	0.032(7)	-0.005(7)
C17	0.078(8)	0.080(8)	0.055(6)	0.030(8)	-0.002(7)	0.007(5)
C18	0.085(10)	0.098(11)	0.081(9)	0.046(8)	0.021(8)	0.002(8)
C19	0.070(7)	0.028(5)	0.058(6)	0.007(4)	-0.001(5)	0.000(4)
C20	0.076(7)	0.044(6)	0.046(5)	0.000(5)	-0.011(5)	-0.006(4)
C21	0.072(7)	0.039(6)	0.053(6)	0.008(5)	-0.004(5)	0.010(5)
C22	0.040(5)	0.032(5)	0.071(7)	0.005(4)	0.003(5)	-0.002(4)
C23	0.044(5)	0.069(6)	0.045(5)	0.014(6)	0.003(4)	0.001(4)
C24	0.063(7)	0.045(6)	0.050(6)	-0.004(5)	-0.009(5)	0.012(5)
C25	0.034(4)	0.030(4)	0.045(4)	-0.011(4)	-0.002(4)	0.002(3)
C26	0.036(4)	0.028(4)	0.045(4)	0.003(4)	0.002(4)	0.006(3)
C27	0.034(5)	0.029(5)	0.043(5)	-0.004(3)	0.003(3)	-0.001(3)
C15	0.069(8)	0.084(10)	0.114(11)	0.028(8)	0.030(8)	-0.005(9)

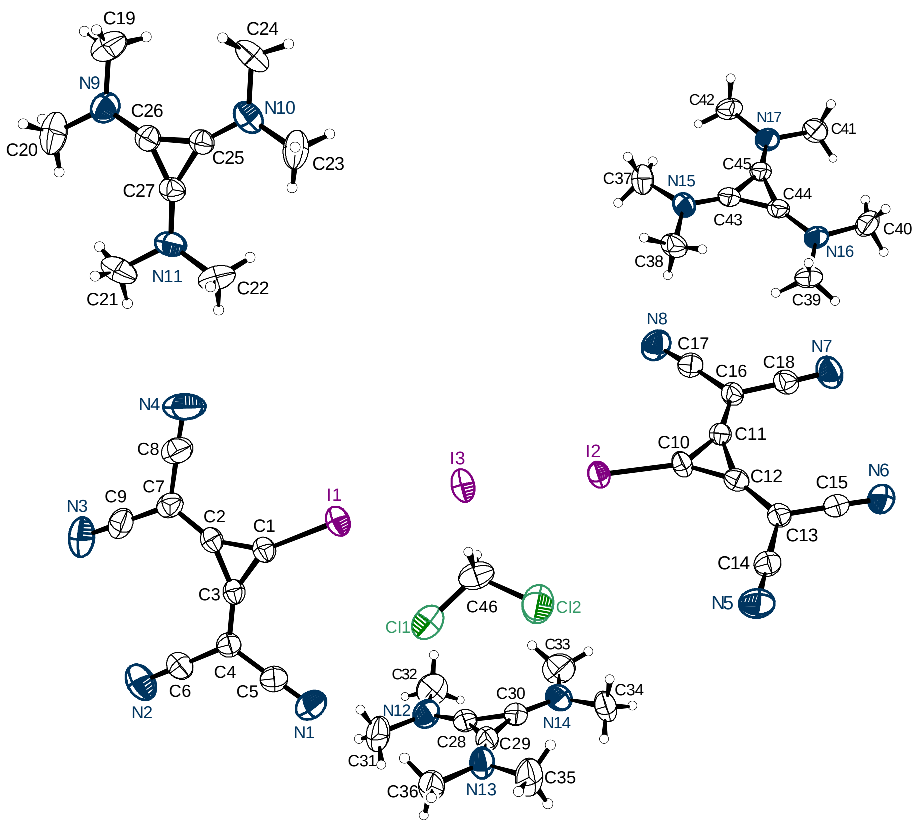

Figure S44. Crystal structure of the co-crystal between two molecules of **1** and iodide.

Table S12. Fractional Coordinates and Isotropic Thermal Parameters of the crystal structure of the co-crystal **1**···I···**1**.

Atom	x/a	y/b	z/c	U [Å²]
I1	0.24116(4)	0.10429(5)	0.33110(2)	
N1	-0.2462(7)	0.3600(12)	0.34935(17)	
N2	-0.1584(8)	0.4260(11)	0.44982(16)	
N3	0.2515(10)	0.3022(13)	0.48315(15)	
N4	0.5769(8)	0.0641(13)	0.4186(2)	
C1	0.1796(6)	0.1862(8)	0.37424(13)	
C2	0.2160(6)	0.2080(8)	0.40482(13)	
C3	0.0720(6)	0.2562(8)	0.39260(13)	
C4	-0.0705(6)	0.3247(9)	0.39662(14)	
C5	-0.1698(7)	0.3424(10)	0.37062(17)	
C6	-0.1197(7)	0.3801(9)	0.42613(16)	
C7	0.3177(7)	0.1954(9)	0.42907(15)	
C8	0.4623(8)	0.1239(12)	0.42372(18)	
C9	0.2798(8)	0.2532(11)	0.45899(16)	
Cl1	-0.0127(2)	0.4843(3)	0.28221(5)	
Cl2	0.0219(3)	0.4241(4)	0.21652(5)	
C46	0.1139(8)	0.4442(12)	0.2528(2)	
H46A	0.16930	0.33343	0.25719	0.0780
H46B	0.18568	0.54318	0.25215	0.0780
I2	0.32290(4)	-0.03268(5)	0.18386(2)	
N5	-0.0640(6)	-0.2210(11)	0.10449(16)	
N6	0.2229(6)	-0.0541(11)	0.02820(12)	
N7	0.6244(7)	0.1415(10)	0.04577(13)	
N8	0.7485(7)	0.2559(10)	0.14399(15)	
C10	0.3465(6)	-0.0110(8)	0.13675(12)	
C11	0.4373(6)	0.0439(8)	0.11330(13)	
C12	0.2940(5)	-0.0336(8)	0.10738(12)	

C13	0.1843(6)	-0.0855(8)	0.08603(13)	
C14	0.0458(6)	-0.1600(9)	0.09599(14)	
C15	0.2073(6)	-0.0671(9)	0.05380(13)	
C16	0.5672(6)	0.1218(8)	0.10327(12)	
C17	0.6717(6)	0.1934(9)	0.12535(14)	
C18	0.5988(6)	0.1333(9)	0.07153(14)	
I3	0.30027(5)	0.92700(5)	0.25994(2)	
N9	0.3619(6)	0.7220(9)	0.45762(13)	
N10	0.2897(6)	0.6397(9)	0.37559(13)	
N11	-0.0051(6)	0.8404(8)	0.42244(14)	
C19	0.5147(8)	0.6492(12)	0.4542(2)	
H19A	0.51264	0.51956	0.45627	0.1000
H19B	0.58026	0.69980	0.46985	0.1000
H19C	0.55091	0.68094	0.43434	0.1000
C20	0.3078(12)	0.7696(14)	0.48787(18)	
H20A	0.21104	0.82651	0.48575	0.1130
H20B	0.37744	0.85183	0.49774	0.1130
H20C	0.29945	0.66170	0.50008	0.1130
C21	-0.0621(9)	0.8897(12)	0.4525(2)	
H21A	-0.13703	0.98268	0.45009	0.0950
H21B	0.01926	0.93381	0.46537	0.0950
H21C	-0.10589	0.78487	0.46187	0.0950
C22	-0.1092(7)	0.8343(11)	0.3960(2)	
H22A	-0.05350	0.84114	0.37739	0.0860
H22B	-0.17744	0.93504	0.39685	0.0860
H22C	-0.16501	0.72286	0.39625	0.0860
C23	0.1877(10)	0.6267(10)	0.34913(15)	
H23A	0.23946	0.66140	0.33096	0.0870
H23B	0.10349	0.70614	0.35191	0.0870
H23C	0.15255	0.50406	0.34702	0.0870
C24	0.4425(8)	0.5715(13)	0.37272(19)	
H24A	0.45760	0.53399	0.35191	0.0960
H24B	0.45760	0.46985	0.38624	0.0960
H24C	0.51290	0.66567	0.37812	0.0960
C25	0.2422(6)	0.6949(8)	0.40240(14)	
C26	0.2698(7)	0.7275(8)	0.43342(14)	
C27	0.1312(6)	0.7689(8)	0.42018(14)	
N12	0.7450(7)	0.8914(9)	0.28519(14)	
N13	0.5783(6)	0.4826(9)	0.25078(12)	
N14	0.7740(6)	0.8113(10)	0.20225(13)	
C28	0.7156(6)	0.7901(10)	0.26086(14)	
C29	0.6518(7)	0.6371(10)	0.24761(14)	
C30	0.7266(6)	0.7602(10)	0.22945(14)	
C31	0.6885(11)	0.8381(15)	0.31488(17)	
H31A	0.75767	0.75481	0.32460	0.1070
H31B	0.59222	0.78062	0.31207	0.1070
H31C	0.67820	0.94368	0.32757	0.1070
C32	0.8296(10)	1.0591(13)	0.2821(2)	
H32A	0.81464	1.13420	0.29979	0.1100
H32B	0.79532	1.12223	0.26396	0.1100
H32C	0.93464	1.03155	0.28055	0.1100
C33	0.8726(10)	0.9689(17)	0.2006(2)	
H33A	0.81414	1.07767	0.20246	0.1270
H33B	0.92174	0.96912	0.18119	0.1270
H33C	0.94679	0.96362	0.21694	0.1270
C34	0.7367(10)	0.7061(14)	0.17483(16)	
H34A	0.80897	0.61050	0.17263	0.0970
H34B	0.73830	0.78369	0.15713	0.0970
H34C	0.63810	0.65461	0.17663	0.0970
C35	0.5305(11)	0.3794(13)	0.22399(19)	
H35A	0.48969	0.26521	0.23035	0.1080

H35B	0.61518	0.35833	0.21121	0.1080
H35C	0.45494	0.44619	0.21264	0.1080
C36	0.5193(8)	0.4336(12)	0.28039(15)	
H36A	0.59756	0.44407	0.29589	0.0830
H36B	0.48339	0.31080	0.27967	0.0830
H36C	0.43800	0.51352	0.28522	0.0830
N15	0.2265(5)	0.4222(8)	0.12020(11)	
N16	0.1066(5)	0.4182(8)	0.03902(11)	
N17	0.4829(5)	0.5866(8)	0.06103(11)	
C37	0.3306(8)	0.4783(11)	0.14450(15)	
H37A	0.27857	0.55137	0.15908	0.0780
H37B	0.41063	0.54766	0.13590	0.0780
H37C	0.37157	0.37296	0.15463	0.0780
C38	0.0791(7)	0.355(1)	0.12875(16)	
H38A	0.08810	0.29048	0.14786	0.0730
H38B	0.04061	0.27493	0.11304	0.0730
H38C	0.01146	0.45548	0.13098	0.0730
C39	-0.0389(6)	0.3434(11)	0.04773(16)	
H39A	-0.02912	0.21525	0.05117	0.0740
H39B	-0.11190	0.36486	0.03152	0.0740
H39C	-0.07097	0.40123	0.06620	0.0740
C40	0.1445(7)	0.4189(14)	0.00682(15)	
H40A	0.06666	0.47974	-0.00489	0.0860
H40B	0.15364	0.29628	-0.00033	0.0860
H40C	0.23821	0.48128	0.00435	0.0860
C41	0.5179(7)	0.6162(12)	0.02881(15)	
H41A	0.58276	0.71977	0.02716	0.0770
H41B	0.42653	0.63739	0.01716	0.0770
H41C	0.56758	0.51095	0.02094	0.0770
C42	0.6022(6)	0.6182(10)	0.08387(16)	
H42A	0.55901	0.63436	0.10368	0.0680
H42B	0.65720	0.72528	0.07854	0.0680
H42C	0.66897	0.51588	0.08449	0.0680
C43	0.2564(5)	0.4543(8)	0.09094(12)	
C44	0.2119(5)	0.4495(8)	0.06060(12)	
C45	0.3540(6)	0.5122(8)	0.06866(13)	

Table S13. Anisotropic Displacement Parameters of the crystal structure of the co-crystal **1**···I···**1**.

Atom	U_{11}	U_{22}	U_{33}	U_{12}	U_{13}	U_{23}
I1	0.0564(2)	0.0354(2)	0.03653(18)	0.00160(16)	0.01046(14)	-0.00082(14)
N1	0.054(3)	0.095(6)	0.059(4)	0.018(3)	-0.010(3)	0.002(3)
N2	0.086(4)	0.068(5)	0.058(4)	0.007(4)	0.026(3)	-0.008(4)
N3	0.104(5)	0.083(5)	0.039(3)	-0.007(4)	-0.004(3)	0.002(3)
N4	0.046(3)	0.093(6)	0.123(7)	0.015(4)	0.009(4)	0.026(5)
C1	0.042(3)	0.036(3)	0.034(3)	0.002(2)	0.005(2)	-0.001(2)
C2	0.041(3)	0.029(3)	0.035(3)	-0.003(2)	0.006(2)	0.004(2)
C3	0.041(3)	0.031(3)	0.032(3)	0.001(2)	0.003(2)	0.002(2)
C4	0.041(3)	0.040(3)	0.039(3)	0.008(2)	0.004(2)	0.003(2)
C5	0.044(3)	0.050(4)	0.050(4)	0.010(3)	0.002(3)	0.001(3)
C6	0.050(3)	0.040(3)	0.048(4)	0.006(3)	0.011(3)	0.005(3)
C7	0.040(3)	0.040(3)	0.047(3)	-0.001(2)	-0.003(2)	0.008(3)
C8	0.044(4)	0.059(4)	0.069(4)	0.000(3)	-0.004(3)	0.018(4)
C9	0.059(4)	0.056(4)	0.044(4)	-0.007(3)	-0.009(3)	0.012(3)
Cl1	0.0773(11)	0.0700(13)	0.0694(11)	0.003(1)	-0.0167(9)	0.0019(10)
Cl2	0.1059(16)	0.0924(18)	0.0672(12)	0.0184(14)	-0.0073(11)	-0.0043(13)
C46	0.053(4)	0.049(4)	0.091(6)	0.000(3)	-0.011(3)	0.006(4)
I2	0.04018(17)	0.0395(2)	0.02894(16)	-0.00558(14)	0.00528(12)	-0.00394(13)
N5	0.040(3)	0.079(5)	0.068(4)	-0.017(3)	0.003(3)	0.001(3)

N6	0.048(3)	0.080(4)	0.036(3)	0.001(3)	-0.002(2)	0.003(3)
N7	0.059(3)	0.073(4)	0.043(3)	-0.015(3)	0.014(2)	-0.006(3)
N8	0.049(3)	0.073(4)	0.057(3)	-0.020(3)	-0.007(3)	0.000(3)
C10	0.039(3)	0.038(3)	0.027(2)	-0.007(2)	0.0078(19)	-0.004(2)
C11	0.030(2)	0.035(3)	0.032(3)	0.000(2)	0.000(2)	-0.006(2)
C12	0.031(2)	0.030(3)	0.038(3)	-0.002(2)	0.0049(19)	0.000(2)
C13	0.032(2)	0.040(3)	0.034(3)	-0.003(2)	0.002(2)	-0.004(2)
C14	0.035(3)	0.047(3)	0.042(3)	-0.007(2)	-0.001(2)	-0.002(3)
C15	0.030(2)	0.048(3)	0.039(3)	0.002(2)	-0.002(2)	0.000(3)
C16	0.033(2)	0.041(3)	0.032(3)	-0.006(2)	0.004(2)	-0.003(2)
C17	0.035(3)	0.046(3)	0.043(3)	-0.009(2)	0.002(2)	0.001(3)
C18	0.036(3)	0.043(3)	0.042(3)	-0.006(2)	0.006(2)	-0.002(3)
I3	0.0652(2)	0.0398(2)	0.03215(18)	-0.00252(17)	0.00866(15)	-0.00210(15)
N9	0.055(3)	0.053(3)	0.046(3)	0.004(3)	-0.009(2)	-0.003(3)
N10	0.054(3)	0.057(4)	0.047(3)	-0.003(3)	0.012(2)	-0.008(3)
N11	0.036(2)	0.049(3)	0.060(3)	0.005(2)	0.009(2)	0.003(3)
C19	0.050(4)	0.068(5)	0.080(5)	0.001(3)	-0.019(4)	0.011(4)
C20	0.115(7)	0.068(6)	0.042(4)	-0.006(5)	-0.015(4)	0.000(4)
C21	0.053(4)	0.061(5)	0.078(5)	0.008(3)	0.025(4)	-0.008(4)
C22	0.038(3)	0.050(4)	0.084(5)	0.003(3)	-0.012(3)	0.000(4)
C23	0.094(5)	0.041(4)	0.039(3)	-0.004(4)	-0.004(3)	0.005(3)
C24	0.057(4)	0.073(5)	0.064(4)	-0.001(4)	0.029(3)	-0.011(4)
C25	0.036(3)	0.033(3)	0.039(3)	-0.001(2)	0.005(2)	0.004(2)
C26	0.041(3)	0.035(3)	0.040(3)	-0.001(2)	0.002(2)	0.003(2)
C27	0.034(3)	0.036(3)	0.043(3)	-0.002(2)	0.005(2)	0.003(2)
N12	0.055(3)	0.058(4)	0.051(3)	-0.012(3)	-0.006(3)	-0.010(3)
N13	0.061(3)	0.056(3)	0.039(3)	-0.024(3)	0.001(2)	-0.003(3)
N14	0.046(3)	0.066(4)	0.048(3)	-0.008(3)	0.008(2)	0.011(3)
C28	0.036(3)	0.050(4)	0.042(3)	-0.008(3)	0.000(2)	-0.003(3)
C29	0.042(3)	0.052(4)	0.037(3)	-0.009(3)	0.002(2)	-0.002(3)
C30	0.036(3)	0.053(4)	0.041(3)	-0.004(3)	0.001(2)	0.002(3)
C31	0.091(6)	0.079(6)	0.042(4)	-0.010(4)	-0.009(4)	-0.012(4)
C32	0.079(5)	0.061(5)	0.079(6)	-0.019(4)	-0.005(4)	-0.020(4)
C33	0.065(4)	0.107(8)	0.082(6)	-0.031(5)	0.002(4)	0.039(6)
C34	0.071(5)	0.087(6)	0.036(3)	0.011(4)	0.011(3)	0.001(4)
C35	0.089(6)	0.076(6)	0.050(4)	-0.043(5)	-0.001(4)	-0.012(4)
C36	0.058(4)	0.063(5)	0.045(3)	-0.021(3)	0.001(3)	0.006(3)
N15	0.039(2)	0.055(3)	0.034(2)	-0.002(2)	0.0073(18)	0.001(2)
N16	0.027(2)	0.063(3)	0.039(2)	-0.004(2)	0.0002(17)	-0.001(2)
N17	0.029(2)	0.054(3)	0.042(2)	-0.006(2)	0.0039(18)	0.004(2)
C37	0.069(4)	0.048(4)	0.039(3)	-0.005(3)	0.000(3)	-0.006(3)
C38	0.041(3)	0.055(4)	0.049(4)	-0.001(3)	0.015(3)	0.010(3)
C39	0.027(3)	0.067(4)	0.055(4)	-0.005(3)	0.002(2)	-0.001(3)
C40	0.043(3)	0.091(6)	0.037(3)	-0.001(3)	-0.006(2)	0.010(4)
C41	0.037(3)	0.071(5)	0.046(3)	-0.007(3)	0.012(2)	0.010(3)
C42	0.030(3)	0.048(4)	0.060(4)	-0.005(2)	-0.004(2)	-0.005(3)
C43	0.027(2)	0.036(3)	0.040(3)	0.001(2)	0.0037(19)	-0.001(2)
C44	0.026(2)	0.038(3)	0.037(3)	0.002(2)	0.0045(18)	0.002(2)
C45	0.028(2)	0.037(3)	0.035(3)	0.001(2)	0.002(2)	0.001(2)

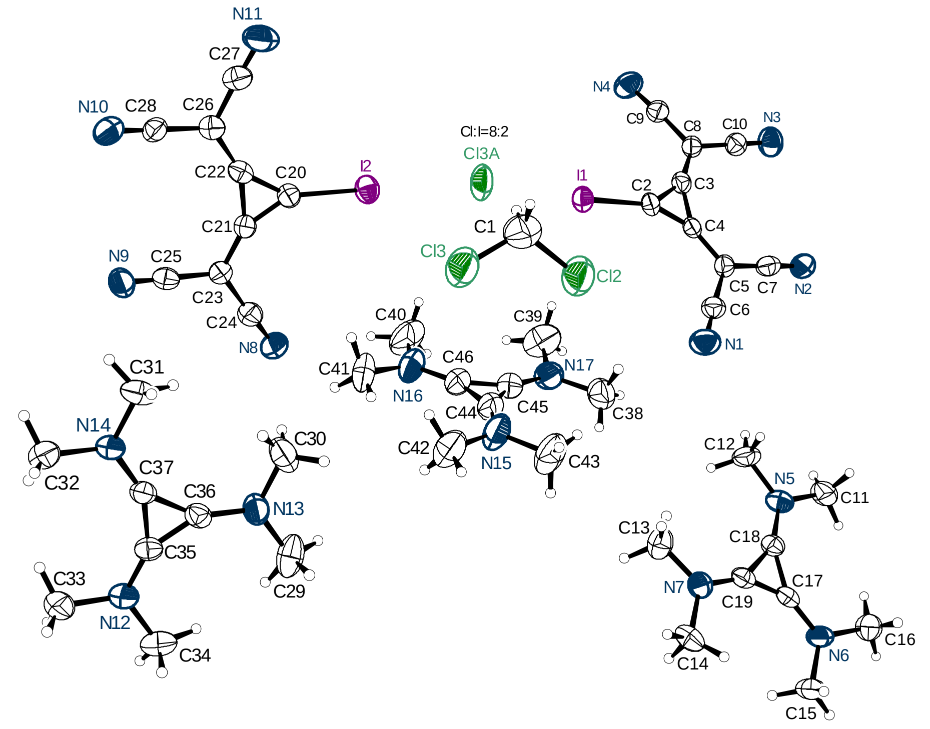

Figure S45. Crystal structure of the co-crystal of **1** with Cl:I = 8:1.

Table S14. Fractional Coordinates and Isotropic Thermal Parameters of the crystal structure of the co-crystal **1**···I$^-$/Cl$^-$···**1**.

Atom	S.O.F.	x/a	y/b	z/c	U [Å²]
I1		0.56151(4)	-0.12427(3)	0.36777(2)	
I2		0.73027(5)	0.08674(4)	0.63769(2)	
N1		0.3248(12)	0.1817(8)	0.2074(4)	
N2		0.4075(13)	-0.1909(9)	0.0575(4)	
N3		0.5971(11)	-0.5753(9)	0.0933(3)	
N4		0.8013(11)	-0.5929(9)	0.2871(4)	
N8		1.0339(12)	0.5965(9)	0.7057(3)	
N9		1.0159(11)	0.5830(8)	0.9023(3)	
N10		0.8641(14)	0.2033(10)	0.9469(4)	
N11		0.6433(14)	-0.1817(9)	0.7974(4)	
C2		0.5434(11)	-0.1972(9)	0.2730(3)	
C3		0.5703(9)	-0.3107(8)	0.2270(3)	
C4		0.4966(12)	-0.1736(9)	0.2160(4)	
C5		0.4296(10)	-0.0883(8)	0.1729(3)	
C6		0.3704(10)	0.0622(8)	0.1927(4)	
C7		0.4176(11)	-0.1448(9)	0.1091(4)	
C8		0.6337(10)	-0.4522(8)	0.2073(3)	
C9		0.7221(10)	-0.5339(8)	0.2508(3)	
C10		0.6145(10)	-0.5200(8)	0.1437(4)	
C20		0.8065(11)	0.1843(9)	0.7283(4)	
C21		0.8776(9)	0.3094(8)	0.7720(3)	
C22		0.8163(11)	0.1731(9)	0.7897(4)	
C23		0.954(1)	0.4559(8)	0.7896(3)	
C24		0.9964(11)	0.5361(9)	0.7436(4)	
C25		0.9889(10)	0.5273(8)	0.8521(4)	
C26		0.7844(11)	0.0902(9)	0.8326(4)	
C27		0.7059(12)	-0.0599(9)	0.8147(4)	
C28		0.8293(12)	0.1539(9)	0.8960(4)	
Cl3A	0.809(4)	0.5746(3)	-0.0446(2)	0.50636(6)	
I3B	0.191(4)	0.5746(3)	-0.0446(2)	0.50636(6)	

Cl2	0.0540(5)	0.1976(4)	0.43218(14)	
Cl3	0.1719(5)	0.2847(4)	0.56379(14)	
C1	0.107(2)	0.1341(15)	0.5021(6)	
H1A	-0.00530	0.07696	0.50989	0.1050
H1AB	0.21194	0.06409	0.49888	0.1050
N5	0.0673(9)	0.5892(6)	0.1213(3)	
N6	-0.109(1)	0.9395(7)	0.0778(3)	
N7	-0.0351(10)	0.9099(7)	0.2395(3)	
C11	0.0622(14)	0.5178(9)	0.0578(4)	
H11A	-0.04770	0.44565	0.04569	0.0770
H11B	0.17854	0.46393	0.05237	0.0770
H11C	0.05227	0.59558	0.03240	0.0770
C12	0.1104(11)	0.4947(9)	0.1680(4)	
H12A	-0.00575	0.43970	0.17281	0.0730
H12B	0.16128	0.55979	0.20706	0.0730
H12C	0.20437	0.42178	0.15509	0.0730
C13	0.0380(14)	0.8315(12)	0.2874(4)	
H13A	0.11109	0.74663	0.27036	0.0800
H13B	-0.06771	0.79270	0.30518	0.0800
H13C	0.12017	0.90200	0.31926	0.0800
C14	-0.0950(12)	1.0640(8)	0.2564(4)	
H14A	-0.19109	1.08509	0.22423	0.0750
H14B	0.01444	1.13582	0.26134	0.0750
H14C	-0.14870	1.07484	0.29496	0.0750
C15	-0.1676(14)	1.0938(8)	0.0953(4)	
H15A	-0.15805	1.14857	0.06189	0.0760
H15B	-0.08529	1.14572	0.13199	0.0760
H15C	-0.29913	1.09102	0.10392	0.0760
C16	-0.1376(15)	0.8678(10)	0.0139(4)	
H16A	-0.27139	0.83483	0.00158	0.0780
H16B	-0.05919	0.77962	0.00755	0.0780
H16C	-0.10157	0.94045	-0.01079	0.0780
C17	-0.0619(11)	0.8586(8)	0.1205(4)	
C18	0.0025(10)	0.7263(8)	0.1368(3)	
C19	-0.0336(10)	0.8480(8)	0.1807(4)	
N12	0.4614(9)	0.4211(7)	0.8794(3)	
N13	0.2646(12)	0.0966(9)	0.7596(3)	
N14	0.3266(10)	0.0621(7)	0.9208(3)	
C29	0.2822(14)	0.1861(15)	0.7130(4)	
H29A	0.33303	0.12427	0.67846	0.0980
H29B	0.36808	0.27506	0.73006	0.0980
H29C	0.15721	0.21937	0.69886	0.0980
C30	0.1928(16)	-0.0607(11)	0.7420(5)	
H30A	0.11314	-0.07548	0.70231	0.1040
H30B	0.11745	-0.08538	0.77284	0.1040
H30C	0.29939	-0.12736	0.73909	0.1040
C31	0.2464(13)	-0.0936(8)	0.9029(5)	
H31A	0.10783	-0.09273	0.89607	0.0820
H31B	0.28482	-0.14982	0.93551	0.0820
H31C	0.29276	-0.14288	0.86522	0.0820
C32	0.3534(15)	0.1329(10)	0.9861(4)	
H32A	0.46069	0.20779	0.99446	0.0840
H32B	0.37899	0.05467	1.01073	0.0840
H32C	0.23833	0.18343	0.99654	0.0840
C33	0.5106(14)	0.4934(9)	0.9437(4)	
H33A	0.63162	0.55202	0.94903	0.0860
H33B	0.52200	0.41521	0.96895	0.0860
H33C	0.41129	0.56126	0.95627	0.0860
C34	0.4732(12)	0.5155(9)	0.8339(4)	
H34A	0.47515	0.45072	0.79369	0.0790
H34B	0.58949	0.58104	0.84476	0.0790

H34C	0.36304	0.57850	0.83245	0.0790
C35	0.386(1)	0.2818(8)	0.8632(3)	
C36	0.3146(11)	0.1592(8)	0.8186(4)	
C37	0.3370(11)	0.1462(9)	0.8795(4)	
N15	0.1233(14)	0.6886(11)	0.5025(3)	
N16	0.5651(13)	0.5521(11)	0.5696(4)	
N17	0.4193(12)	0.4467(8)	0.4054(3)	
C38	0.2895(16)	0.4557(12)	0.3504(4)	
H38A	0.35586	0.43083	0.31437	0.1000
H38B	0.24482	0.55882	0.35344	0.1000
H38C	0.18098	0.38348	0.34641	0.1000
C39	0.565(2)	0.3355(14)	0.4010(6)	
H39A	0.62148	0.33456	0.36334	0.1270
H39B	0.50805	0.23462	0.40040	0.1270
H39C	0.66358	0.36268	0.43634	0.1270
C40	0.7274(18)	0.4596(17)	0.5633(7)	
H40A	0.68612	0.35216	0.55696	0.1390
H40B	0.81902	0.48594	0.60044	0.1390
H40C	0.78690	0.47807	0.52811	0.1390
C41	0.526(2)	0.6252(16)	0.6323(5)	
H41A	0.42078	0.69221	0.62877	0.1420
H41B	0.63967	0.68464	0.65439	0.1420
H41C	0.49325	0.54646	0.65460	0.1420
C42	0.100(2)	0.7794(14)	0.5605(5)	
H42A	0.20954	0.85088	0.57480	0.1300
H42B	0.08892	0.71335	0.59022	0.1300
H42C	-0.01555	0.83583	0.55625	0.1300
C43	-0.003(2)	0.7042(17)	0.4491(5)	
H43A	0.01889	0.80497	0.43965	0.1290
H43B	-0.13445	0.69201	0.45676	0.1290
H43C	0.02051	0.62618	0.41438	0.1290
C44	0.2778(15)	0.6115(10)	0.4955(4)	
C45	0.3869(13)	0.5214(9)	0.4597(4)	
C46	0.4411(14)	0.5608(10)	0.5219(4)	

Table S15. Anisotropic Displacement Parameters **1**···I^-/Cl^-···**1**.

Atom	U_{11}	U_{22}	U_{33}	U_{12}	U_{13}	U_{23}
I1	0.0391(3)	0.0430(3)	0.0355(2)	0.00568(19)	-0.00111(19)	0.0021(2)
I2	0.0474(3)	0.0508(3)	0.0388(3)	0.0028(2)	0.0105(2)	0.0024(2)
N1	0.063(4)	0.046(4)	0.074(5)	0.011(3)	0.007(4)	0.014(4)
N2	0.069(5)	0.051(4)	0.041(4)	-0.004(4)	0.004(3)	0.014(3)
N3	0.062(4)	0.061(4)	0.046(4)	0.008(3)	0.006(3)	-0.002(3)
N4	0.064(4)	0.054(4)	0.062(4)	0.018(3)	0.008(4)	0.020(4)
N8	0.076(5)	0.056(4)	0.049(4)	-0.009(4)	0.007(4)	0.019(3)
N9	0.063(4)	0.052(4)	0.043(4)	-0.007(3)	0.007(3)	0.001(3)
N10	0.078(5)	0.055(5)	0.051(5)	0.008(4)	0.009(4)	0.021(4)
N11	0.094(6)	0.046(4)	0.080(6)	-0.010(4)	0.020(5)	0.017(4)
C2	0.036(4)	0.036(4)	0.031(4)	0.000(3)	-0.001(3)	-0.001(3)
C3	0.031(3)	0.039(4)	0.035(3)	-0.004(3)	0.000(3)	0.005(3)
C4	0.037(4)	0.031(4)	0.029(3)	-0.005(3)	0.001(3)	0.000(3)
C5	0.034(4)	0.038(4)	0.035(3)	0.002(3)	0.000(3)	0.004(3)
C6	0.041(4)	0.036(4)	0.048(4)	0.003(3)	0.004(3)	0.009(3)
C7	0.040(4)	0.037(4)	0.055(5)	0.003(3)	0.004(3)	0.017(4)
C8	0.037(3)	0.035(3)	0.035(3)	0.004(3)	0.000(3)	0.004(3)
C9	0.044(4)	0.036(3)	0.041(4)	0.007(3)	0.006(3)	0.006(3)
C10	0.037(4)	0.041(4)	0.044(4)	0.004(3)	0.002(3)	0.007(3)
C20	0.037(4)	0.040(4)	0.035(4)	-0.003(3)	0.009(3)	0.005(3)
C21	0.033(3)	0.037(3)	0.033(3)	0.003(3)	0.007(3)	0.006(3)

C22	0.026(3)	0.032(4)	0.047(4)	0.001(3)	0.002(3)	0.005(3)
C23	0.041(4)	0.038(3)	0.037(3)	0.001(3)	0.006(3)	0.009(3)
C24	0.046(4)	0.042(4)	0.048(4)	-0.004(3)	0.002(3)	0.009(3)
C25	0.039(4)	0.035(3)	0.050(4)	-0.001(3)	0.010(3)	0.010(3)
C26	0.039(4)	0.038(4)	0.044(4)	0.004(3)	0.010(3)	0.009(3)
C27	0.055(5)	0.043(4)	0.053(4)	0.005(3)	0.016(4)	0.017(4)
C28	0.051(5)	0.041(4)	0.035(4)	0.008(3)	0.011(3)	0.012(3)
Cl3A	0.0868(12)	0.0833(12)	0.0338(8)	0.0121(9)	-0.0008(7)	-0.0025(7)
I3B	0.0868(12)	0.0833(12)	0.0338(8)	0.0121(9)	-0.0008(7)	-0.0025(7)
Cl2	0.100(2)	0.108(2)	0.0633(15)	-0.0154(18)	0.0025(14)	0.0225(15)
Cl3	0.102(2)	0.111(2)	0.0644(16)	-0.0151(19)	0.0016(15)	0.0288(15)
C1	0.102(9)	0.080(8)	0.086(8)	-0.004(7)	0.017(7)	0.026(6)
N5	0.046(3)	0.030(3)	0.048(3)	0.004(2)	0.012(3)	0.005(2)
N6	0.060(4)	0.029(3)	0.042(3)	0.003(3)	0.006(3)	0.011(3)
N7	0.050(4)	0.040(3)	0.034(3)	0.004(3)	0.006(3)	0.003(3)
C11	0.070(5)	0.038(4)	0.043(4)	0.007(4)	0.010(4)	-0.003(3)
C12	0.040(4)	0.035(4)	0.073(5)	0.006(3)	-0.001(4)	0.022(4)
C13	0.046(5)	0.068(6)	0.044(5)	0.012(4)	0.004(4)	0.009(4)
C14	0.059(5)	0.035(4)	0.051(4)	-0.002(3)	0.014(4)	-0.006(3)
C15	0.070(5)	0.030(3)	0.055(5)	0.007(3)	0.012(4)	0.012(3)
C16	0.076(6)	0.049(5)	0.032(4)	0.006(4)	0.009(4)	0.007(3)
C17	0.040(4)	0.024(3)	0.040(4)	-0.003(3)	0.004(3)	-0.001(3)
C18	0.035(3)	0.031(3)	0.037(3)	-0.003(3)	0.007(3)	0.007(3)
C19	0.031(3)	0.026(3)	0.045(4)	-0.001(3)	0.004(3)	0.003(3)
N12	0.051(4)	0.033(3)	0.048(3)	0.001(3)	0.005(3)	0.009(3)
N13	0.068(5)	0.049(4)	0.040(4)	0.014(3)	-0.007(3)	-0.002(3)
N14	0.058(4)	0.029(3)	0.044(4)	0.000(3)	0.000(3)	0.008(3)
C29	0.045(5)	0.112(9)	0.039(5)	0.010(5)	0.010(4)	0.013(5)
C30	0.073(6)	0.053(5)	0.064(6)	0.017(4)	-0.019(5)	-0.015(4)
C31	0.062(5)	0.031(4)	0.072(6)	-0.002(3)	0.008(4)	0.010(4)
C32	0.070(6)	0.046(5)	0.052(5)	-0.001(4)	-0.006(4)	0.017(4)
C33	0.069(6)	0.040(4)	0.056(5)	-0.006(4)	-0.007(4)	0.004(4)
C34	0.052(4)	0.041(4)	0.073(6)	0.005(3)	0.016(4)	0.023(4)
C35	0.036(4)	0.032(3)	0.043(4)	0.008(3)	0.007(3)	0.009(3)
C36	0.036(4)	0.032(4)	0.042(4)	0.007(3)	0.004(3)	0.003(3)
C37	0.038(4)	0.027(4)	0.041(4)	0.001(3)	0.000(3)	0.006(3)
N15	0.094(6)	0.080(6)	0.039(4)	0.042(5)	-0.001(4)	0.009(4)
N16	0.079(6)	0.080(6)	0.050(4)	0.011(4)	-0.014(4)	0.019(4)
N17	0.075(5)	0.050(4)	0.049(4)	0.012(3)	0.014(4)	0.009(3)
C38	0.084(7)	0.064(6)	0.048(5)	-0.009(5)	0.008(5)	0.004(4)
C39	0.105(9)	0.084(7)	0.078(7)	0.041(7)	0.043(7)	0.024(6)
C40	0.074(7)	0.109(9)	0.100(9)	0.024(7)	-0.018(6)	0.049(8)
C41	0.133(11)	0.101(9)	0.039(5)	0.006(8)	-0.022(6)	0.008(5)
C42	0.126(11)	0.087(8)	0.049(6)	0.059(8)	0.015(6)	0.014(5)
C43	0.084(8)	0.109(9)	0.064(7)	0.048(7)	-0.007(6)	0.020(6)
C44	0.076(6)	0.050(5)	0.048(5)	0.018(4)	-0.003(4)	0.007(4)
C45	0.058(5)	0.044(4)	0.049(4)	0.004(4)	0.000(4)	0.008(4)
C46	0.067(5)	0.049(4)	0.050(5)	0.011(4)	-0.006(4)	0.011(4)

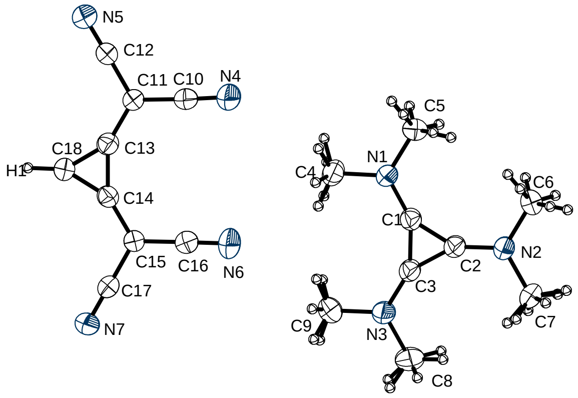

Figure S46. Crystal structure of H-compound **3**.

Table S16. Fractional Coordinates and Isotropic Thermal Parameters of the crystal structure of **3**.

Atom	S.O.F.	x/a	y/b	z/c	U [Å²]
N1		0.57264(17)	0.02318(19)	0.64056(12)	
N2		0.28799(18)	-0.1375(2)	0.84639(12)	
N3		0.16103(18)	0.2382(2)	0.64462(13)	
C1		0.4265(2)	0.0330(2)	0.68353(13)	
C2		0.3194(2)	-0.0275(2)	0.76055(13)	
C3		0.2715(2)	0.1119(2)	0.68436(13)	
C4		0.6097(2)	0.1420(3)	0.54642(15)	
H4A	0.67(2)	0.70368	0.09487	0.51331	0.0720
H4B	0.67(2)	0.63433	0.24453	0.55995	0.0720
H4C	0.67(2)	0.51601	0.16666	0.50246	0.0720
H4D	0.33(2)	0.53233	0.24250	0.53717	0.0720
H4E	0.33(2)	0.60168	0.09285	0.49053	0.0720
H4F	0.33(2)	0.72000	0.17071	0.54802	0.0720
C5		0.6960(2)	-0.1116(2)	0.68542(16)	
H5A	0.36(2)	0.73213	-0.17471	0.63675	0.0720
H5B	0.36(2)	0.65040	-0.18522	0.74551	0.0720
H5C	0.36(2)	0.78847	-0.06553	0.70422	0.0720
H5D	0.64(2)	0.71521	-0.10893	0.75424	0.0720
H5E	0.64(2)	0.79693	-0.09842	0.64548	0.0720
H5F	0.64(2)	0.65886	-0.21811	0.68677	0.0720
C6		0.4141(2)	-0.2682(2)	0.89300(16)	
H6A	0.75(3)	0.45575	-0.33038	0.84472	0.0770
H6B	0.75(3)	0.36872	-0.34376	0.95235	0.0770
H6C	0.75(3)	0.50275	-0.21852	0.91328	0.0770
H6D	0.25(3)	0.42906	-0.26473	0.96218	0.0770
H6E	0.25(3)	0.51609	-0.25134	0.85455	0.0770
H6F	0.25(3)	0.38206	-0.37659	0.89362	0.0770
C7		0.1290(2)	-0.1273(2)	0.89570(15)	
H7A	0.51(2)	0.10444	-0.01835	0.90911	0.0720
H7B	0.51(2)	0.12912	-0.21470	0.95930	0.0720
H7C	0.51(2)	0.04648	-0.14193	0.85185	0.0720
H7D	0.49(2)	0.08225	-0.23163	0.90440	0.0720
H7E	0.49(2)	0.05758	-0.03528	0.85421	0.0720
H7F	0.49(2)	0.14021	-0.10805	0.96166	0.0720
C8		-0.0078(2)	0.2332(3)	0.6780(2)	
H8A	0.78(3)	-0.06081	0.34680	0.66881	0.0940
H8B	0.78(3)	-0.01308	0.17236	0.74929	0.0940
H8C	0.78(3)	-0.06321	0.17706	0.63833	0.0940
H8D	0.22(3)	-0.03059	0.11735	0.70215	0.0940
H8E	0.22(3)	-0.07832	0.29178	0.62166	0.0940
H8F	0.22(3)	-0.02819	0.28709	0.73262	0.0940

C9		0.1958(3)	0.3563(3)	0.54955(17)	
H9A	0.08(3)	0.26343	0.29850	0.50656	0.0910
H9B	0.08(3)	0.25348	0.44303	0.56216	0.0910
H9C	0.08(3)	0.09386	0.40700	0.51587	0.0910
H9D	0.92(3)	0.14375	0.46718	0.54983	0.0910
H9E	0.92(3)	0.15370	0.32265	0.49424	0.0910
H9F	0.92(3)	0.31332	0.35868	0.54052	0.0910
N4		0.8878(2)	0.2602(3)	0.35332(15)	
N5		1.2085(2)	0.4695(2)	0.09818(14)	
N6		0.4373(2)	0.4722(2)	0.35682(14)	
N7		0.2749(2)	0.9237(2)	0.11901(13)	
C10		0.9046(2)	0.3608(2)	0.27866(15)	
C11		0.9297(2)	0.4820(2)	0.18710(14)	
C12		1.0841(2)	0.4742(2)	0.13880(14)	
C13		0.8097(2)	0.6063(2)	0.14455(14)	
C14		0.6492(2)	0.6840(2)	0.14658(13)	
C15		0.4983(2)	0.6890(2)	0.19334(13)	
C16		0.4649(2)	0.5691(2)	0.28334(15)	
C17		0.3743(2)	0.8177(2)	0.15200(14)	
C18		0.7557(2)	0.7439(2)	0.07066(15)	
H1		0.779(3)	0.824(3)	0.0059(18)	0.060(6)

Table S17. Anisotropic Displacement Parameters for H-compound **3**.

Atom	U_{11}	U_{22}	U_{33}	U_{12}	U_{13}	U_{23}
N1	0.0351(8)	0.0459(9)	0.0389(9)	-0.0007(6)	0.0060(6)	-0.0025(7)
N2	0.0371(8)	0.0482(9)	0.0419(9)	-0.0030(7)	0.0053(7)	0.0025(7)
N3	0.0324(8)	0.0585(10)	0.051(1)	0.0019(7)	0.0003(7)	0.0059(8)
C1	0.0349(9)	0.0378(9)	0.0352(9)	-0.0050(7)	-0.0021(7)	-0.0056(7)
C2	0.0330(8)	0.0405(9)	0.0377(10)	-0.0053(7)	0.0009(7)	-0.0066(8)
C3	0.0316(9)	0.0436(10)	0.0364(10)	-0.0059(7)	-0.0002(7)	-0.0044(7)
C4	0.0444(10)	0.0535(11)	0.0414(11)	-0.0116(8)	0.0057(8)	-0.0047(9)
C5	0.0402(10)	0.0436(10)	0.0555(13)	0.0025(8)	0.0041(9)	-0.0097(9)
C6	0.0504(11)	0.0426(10)	0.0503(12)	-0.0004(8)	0.0032(9)	0.0030(9)
C7	0.0437(10)	0.0500(11)	0.0455(11)	-0.0090(8)	0.0121(8)	-0.0066(9)
C8	0.0307(10)	0.0685(14)	0.0802(17)	-0.0016(9)	-0.0028(10)	-0.0069(12)
C9	0.0524(12)	0.0604(13)	0.0517(13)	0.0069(10)	-0.0024(10)	0.0083(10)
N4	0.0546(11)	0.0725(13)	0.0558(12)	0.0056(9)	0.0105(9)	0.0127(10)
N5	0.0434(10)	0.0562(10)	0.0593(12)	0.0009(8)	0.0084(8)	-0.0007(8)
N6	0.0687(12)	0.0650(12)	0.0485(11)	-0.0076(9)	0.0104(9)	0.0086(9)
N7	0.0456(9)	0.0513(10)	0.051(1)	0.0099(8)	0.0049(8)	-0.0010(8)
C10	0.0357(10)	0.0493(11)	0.0480(12)	0.0040(8)	0.0043(8)	-0.0035(9)
C11	0.0340(9)	0.0386(9)	0.040(1)	-0.0013(7)	0.0045(7)	-0.0040(7)
C12	0.0379(10)	0.0363(9)	0.0438(11)	0.0005(7)	0.0015(8)	-0.0024(8)
C13	0.0359(9)	0.0359(9)	0.0395(10)	-0.0055(7)	0.0026(7)	-0.0098(7)
C14	0.0364(9)	0.0307(8)	0.0389(10)	-0.0025(7)	-0.0010(7)	-0.0078(7)
C15	0.0342(9)	0.0336(9)	0.037(1)	-0.0024(7)	0.0017(7)	-0.0054(7)
C16	0.0387(10)	0.044(1)	0.0423(11)	-0.0012(8)	0.0026(8)	-0.0074(9)
C17	0.0372(9)	0.0413(10)	0.0367(10)	-0.0045(8)	0.0071(8)	-0.0075(8)
C18	0.0386(9)	0.0375(9)	0.0424(11)	-0.0039(7)	0.0048(8)	-0.0033(8)

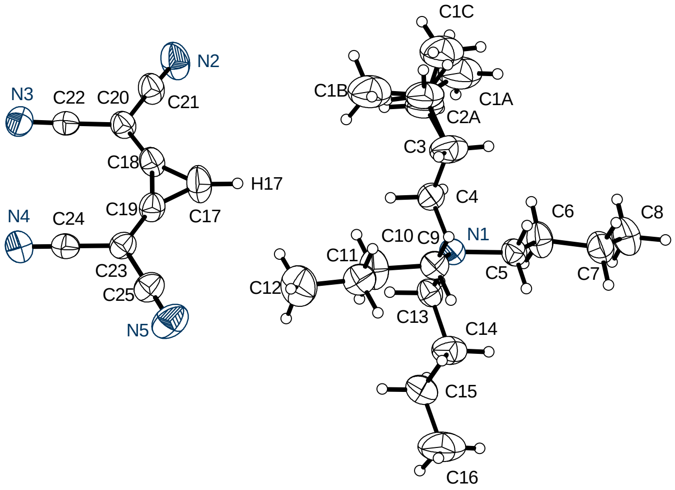

Figure S47. Crystal structure of the tetrabutylammonium salt of the H-compound **3•NBu₄**.

Table S18. Fractional Coordinates and Isotropic Thermal Parameters of the crystal structure of the tetrabutylammonium 1,2-bis(dicyanomethylene)-3-hydro-cyclopropanid **3•NBu₄**.

Atom	S.O.F.	x/a	y/b	z/c	U [Å²]
N1		0.61387(6)	0.36474(9)	0.48705(5)	
C3		0.52412(10)	0.23013(17)	0.40733(11)	
H3A		0.50020	0.28770	0.37533	0.0920
H3B		0.49978	0.22594	0.43517	0.0920
C4		0.60219(8)	0.26143(12)	0.44837(7)	
H4A		0.62571	0.26950	0.42005	0.0560
H4AB		0.62672	0.20178	0.47857	0.0560
C5		0.57842(8)	0.46022(12)	0.44309(7)	
H5A		0.52572	0.44760	0.42029	0.0540
H5AB		0.58667	0.52537	0.46995	0.0540
C6		0.60523(10)	0.48204(15)	0.39355(8)	
H6A		0.65853	0.48694	0.41551	0.0720
H6AB		0.59161	0.42106	0.36298	0.0720
C7		0.57459(10)	0.58442(15)	0.35692(8)	
H7A		0.58708	0.64505	0.38763	0.0720
H7AB		0.52135	0.57870	0.33406	0.0720
C8		0.60271(11)	0.60822(18)	0.30857(9)	
H8A		0.57954	0.67348	0.28446	0.1110
H8AB		0.59171	0.54753	0.27877	0.1110
H8AC		0.65505	0.61903	0.33125	0.1110
C9		0.58041(8)	0.35613(12)	0.53279(7)	
H9A		0.58646	0.42602	0.55503	0.0550
H9AB		0.52799	0.34332	0.50718	0.0550
C10		0.61141(11)	0.26834(16)	0.58305(10)	
H10A		0.60769	0.19841	0.56143	0.0820
H10B		0.66316	0.28309	0.61093	0.0820
C11		0.57274(10)	0.26102(15)	0.62383(8)	
H11A		0.52141	0.24339	0.59606	0.0710
H11B		0.57464	0.33197	0.64384	0.0710
C12		0.60516(14)	0.1765(2)	0.67629(11)	
H12A		0.57540	0.16977	0.69877	0.1310
H12B		0.65416	0.19801	0.70691	0.1310
H12C		0.60681	0.10709	0.65703	0.1310
C13		0.69517(7)	0.38104(12)	0.52443(7)	
H13A		0.71614	0.31915	0.55379	0.0520
H13B		0.71585	0.38104	0.49370	0.0520
C14		0.71762(9)	0.48360(16)	0.56341(9)	
H14A		0.70131	0.54617	0.53398	0.0780

H14B		0.69373	0.48736	0.59163	0.0780
C15		0.79787(9)	0.48992(16)	0.60390(9)	
H15A		0.81352	0.42903	0.63460	0.0750
H15B		0.82154	0.48177	0.57570	0.0750
C16		0.82282(14)	0.5928(2)	0.64075(14)	
H16A		0.87491	0.58916	0.66872	0.1660
H16B		0.79761	0.60375	0.66700	0.1660
H16C		0.81227	0.65301	0.61067	0.1660
C1A	0.443(3)	0.5171(3)	0.1492(5)	0.3112(3)	
H1AA	0.443(3)	0.50249	0.08570	0.28325	0.1510
H1AB	0.443(3)	0.56652	0.16921	0.32092	0.1510
H1AC	0.443(3)	0.48446	0.20910	0.28941	0.1510
C2A	0.443(3)	0.5136(18)	0.1241(10)	0.3721(10)	
H2AA	0.443(3)	0.55199	0.07246	0.39832	0.0870
H2AB	0.443(3)	0.46623	0.09223	0.36288	0.0870
C1B	0.416(3)	0.5212(3)	0.0359(5)	0.4173(3)	
H1BA	0.416(3)	0.51121	-0.03321	0.39467	0.1510
H1BB	0.416(3)	0.48662	0.04756	0.43442	0.1510
H1BC	0.416(3)	0.57030	0.03535	0.45271	0.1510
C2B	0.416(3)	0.5144(19)	0.1245(11)	0.3716(10)	
H2BA	0.416(3)	0.46633	0.12204	0.33376	0.0870
H2BB	0.416(3)	0.55171	0.11685	0.35667	0.0870
C1C	0.140(3)	0.4696(9)	0.0961(16)	0.323(1)	
H1CA	0.140(3)	0.47518	0.02773	0.30476	0.1510
H1CB	0.140(3)	0.44782	0.14953	0.28863	0.1510
H1CC	0.140(3)	0.43835	0.08536	0.34376	0.1510
C2C	0.140(3)	0.5424(8)	0.1353(15)	0.3719(10)	
H2CA	0.140(3)	0.57125	0.16173	0.35077	0.0870
H2CB	0.140(3)	0.56945	0.07735	0.40246	0.0870
N2		0.30755(10)	0.29077(14)	0.15025(7)	
N3		0.38112(8)	0.45110(11)	0.33937(7)	
N4		0.39476(8)	0.29052(12)	0.47928(7)	
N5		0.30509(11)	-0.03664(13)	0.45657(9)	
C17		0.27894(11)	0.09526(15)	0.28060(8)	
H17		0.25565	0.03678	0.25260	0.0810
C18		0.30717(8)	0.19591(13)	0.28878(7)	
C19		0.30760(8)	0.13740(12)	0.34088(7)	
C20		0.32537(8)	0.28799(12)	0.26645(7)	
C21		0.31520(9)	0.29142(13)	0.20202(7)	
C22		0.35647(7)	0.37757(11)	0.30694(7)	
C23		0.32731(8)	0.13319(11)	0.40544(7)	
C24		0.36414(8)	0.22017(12)	0.44625(7)	
C25		0.31454(9)	0.03914(13)	0.43348(8)	

Table S19. Anisotropic Displacement Parameters for the crystal structure of **3•NBu₄**.

**Atom**	**U_{11}**	**U_{22}**	**U_{33}**	**U_{12}**	**U_{13}**	**U_{23}**
N1	0.0406(6)	0.0451(7)	0.0391(6)	0.0068(5)	0.0187(5)	-0.0014(5)
C3	0.056(1)	0.0693(12)	0.0896(14)	0.0045(9)	0.0191(10)	-0.0323(11)
C4	0.0503(8)	0.0445(8)	0.0462(8)	0.0085(6)	0.0235(7)	-0.0038(6)
C5	0.0442(7)	0.0466(8)	0.0419(7)	0.0102(6)	0.0174(6)	0.0002(6)
C6	0.0664(10)	0.0668(11)	0.0536(9)	0.0244(8)	0.0328(8)	0.0153(8)
C7	0.0674(10)	0.061(1)	0.0481(8)	0.0128(8)	0.0234(8)	0.0074(7)
C8	0.0792(12)	0.0860(14)	0.0567(10)	0.0129(10)	0.0303(9)	0.0209(10)
C9	0.0462(8)	0.0515(8)	0.0468(8)	0.0045(6)	0.0270(6)	-0.0032(6)
C10	0.0764(12)	0.0782(12)	0.0697(11)	0.0254(10)	0.0506(10)	0.0210(9)
C11	0.0704(11)	0.0627(10)	0.0584(9)	-0.0039(8)	0.0404(9)	-0.0038(8)
C12	0.1089(17)	0.0973(16)	0.0759(13)	-0.0005(13)	0.0591(13)	0.0145(12)
C13	0.0393(7)	0.0513(8)	0.0397(7)	0.0070(6)	0.0181(6)	0.0034(6)

C14	0.0476(9)	0.0696(11)	0.0699(11)	0.0012(8)	0.0195(8)	-0.0179(9)
C15	0.0512(9)	0.0716(11)	0.0548(9)	-0.0029(8)	0.0151(7)	-0.0029(8)
C16	0.0765(15)	0.112(2)	0.114(2)	-0.0233(14)	0.0163(14)	-0.0448(17)
C1A	0.067(2)	0.100(3)	0.110(3)	-0.0084(19)	0.019(2)	-0.030(2)
C2A	0.0469(15)	0.0720(14)	0.0844(15)	-0.0031(12)	0.0173(14)	-0.0299(12)
C1B	0.067(2)	0.100(3)	0.110(3)	-0.0084(19)	0.019(2)	-0.030(2)
C2B	0.0469(15)	0.0720(14)	0.0844(15)	-0.0031(12)	0.0173(14)	-0.0299(12)
C1C	0.067(2)	0.100(3)	0.110(3)	-0.0084(19)	0.019(2)	-0.030(2)
C2C	0.0469(15)	0.0720(14)	0.0844(15)	-0.0031(12)	0.0173(14)	-0.0299(12)
N2	0.0960(12)	0.0791(11)	0.0483(8)	-0.0332(9)	0.0385(8)	-0.0105(7)
N3	0.0765(9)	0.0462(8)	0.0524(8)	-0.0044(7)	0.0266(7)	-0.0029(6)
N4	0.0670(9)	0.0607(9)	0.0534(8)	-0.0043(7)	0.0192(7)	-0.0089(7)
N5	0.1065(13)	0.0580(9)	0.0856(11)	-0.0023(8)	0.0631(10)	0.0132(8)
C17	0.0909(13)	0.0635(11)	0.0526(9)	-0.0355(10)	0.0369(9)	-0.0160(8)
C18	0.0493(8)	0.0538(9)	0.0401(7)	-0.0103(6)	0.0207(6)	-0.0056(6)
C19	0.0514(8)	0.0442(8)	0.0507(8)	-0.0093(6)	0.0269(7)	-0.0031(6)
C20	0.0456(7)	0.0483(8)	0.0385(7)	-0.0074(6)	0.0186(6)	-0.0014(6)
C21	0.0582(9)	0.0549(9)	0.0461(9)	-0.0170(7)	0.0250(7)	-0.0046(7)
C22	0.0436(7)	0.0428(8)	0.0387(7)	0.0032(6)	0.0175(6)	0.0052(6)
C23	0.0483(8)	0.0415(7)	0.0472(8)	-0.0016(6)	0.0254(6)	0.0022(6)
C24	0.0440(7)	0.0482(8)	0.0431(8)	0.0061(6)	0.0195(6)	0.0045(6)
C25	0.0658(10)	0.0493(9)	0.0587(9)	0.0006(7)	0.0378(8)	0.0038(7)

IV. Computational Details

All calculations were performed as described in our previous report on SMD18[6],[7] solvation model: Computations were performed using Gaussian 09 software suite, rev. E01[8] with the M06-2X[9] DFT functional, applying the def2-TZVP[10] basis set with additional diffuse functions according to Rappoport and Furche[11] on iodine (def2-TZVPD), as described elsewhere.[12] All geometries were fully optimized with *scrf=(SMD,solvent=acetonitrile,read)* on an ultrafine grid. The vdW. radius of iodine was set to 2.74 Å using the *modifysph* command. Frequencies were computed separately from the geometry optimization. Relevant partition functions for vibrations of frequencies below 100 cm^{-1} were corrected according to the method of Grimme.[13] Free energies of association at 1 atm were corrected to a 1 M solution standard state, according to the equation below:

$$D \quad + \quad A \quad \rightleftharpoons \quad DA \qquad\qquad | \ -1.89 \text{ kcal/mol}$$

$$\Delta G = G_{DA} - G_{D} - G_{A} - 1.89 \text{ kcal/mol}$$

In all computations complete dissociation of the ions was assumed and the non-coordinating counterions were omitted. All geometries can be found in an additional xyz file containing the electronic energies and the corrected free enthalpies in hartree.

Gaussian output files were analysed using the GoodVibes software package, which additionally corrected the frequencies for low vibrational modes according to Grimme et al. up to 100 cm^{-1}. Electrostatic potentials were plotted using GaussView 6.1.1. The NCI-plots[14] were generated using the website http://www.ch.ic.ac.uk/rzepa/cub2nci/.

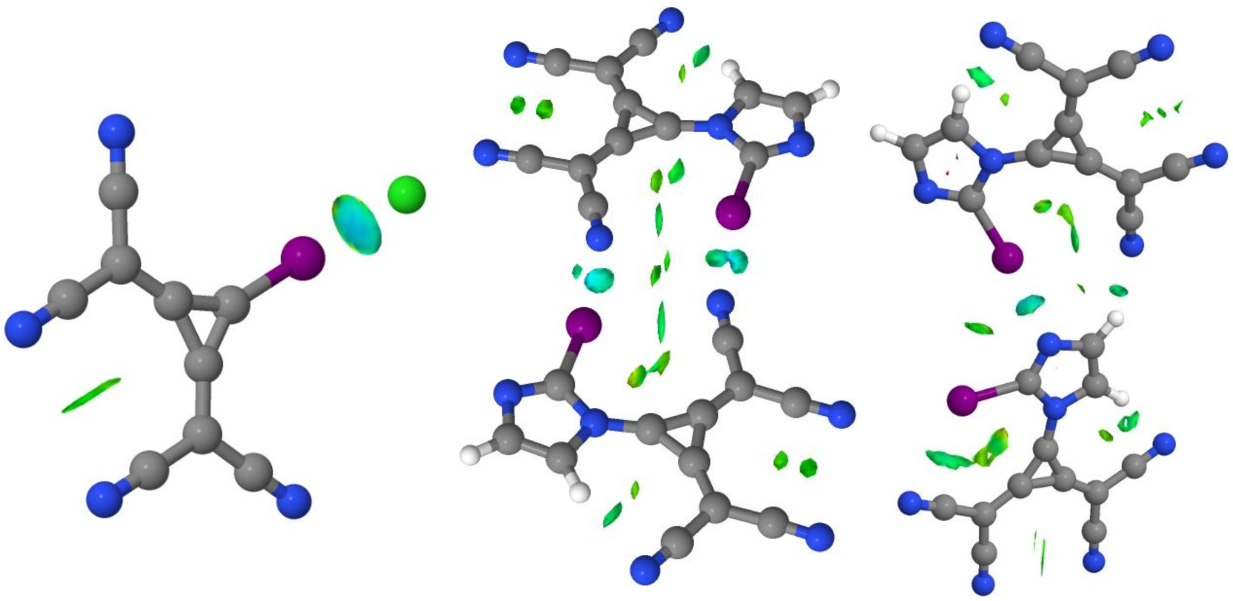

Figure S48. NCI-plots of the XB-bonded complexes [**1**···Cl]$^{2-}$ (left), [**6**···**6**]$^{2-}_{2XB}$ (middle), [**6**···**6**]$^{2-}_{1XB}$. (right) NCI-plots visualise non-covalent interaction with blue surfaces for attractive and red ones for repulsive interactions. Inhere, only attractive forces are observed between the involved anions.

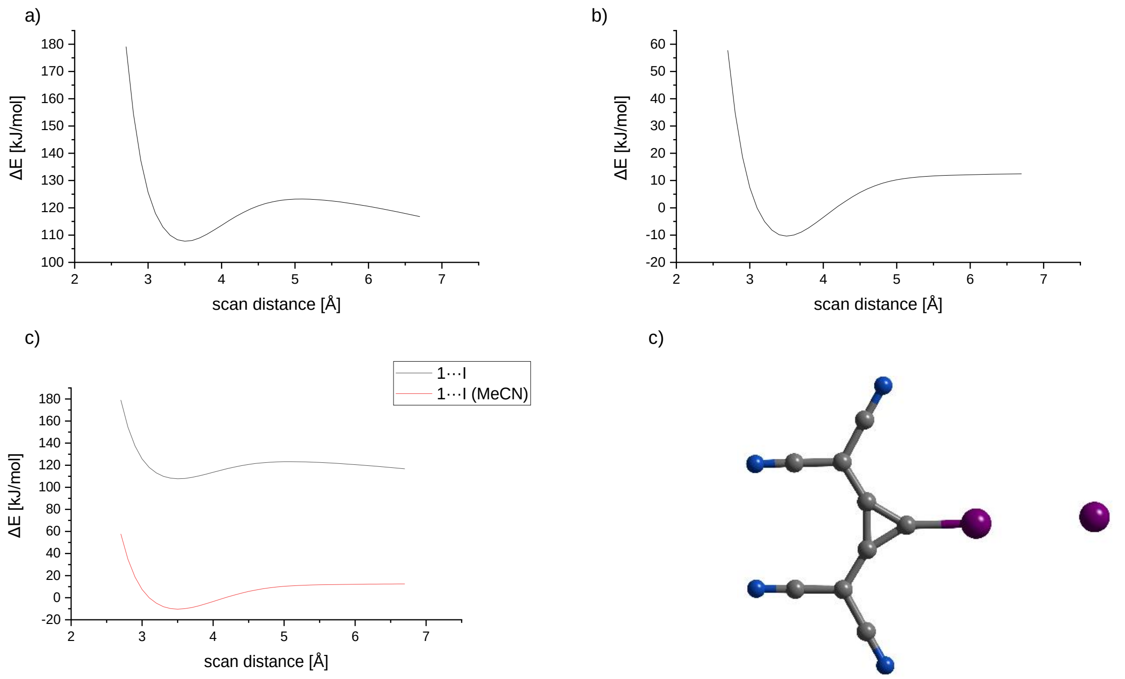

Figure S49. Scan of **1** with iodide in the gas phase (a) and in acetonitrile (b). d) showes the minimum structure which is almost identical for both mcalculations.

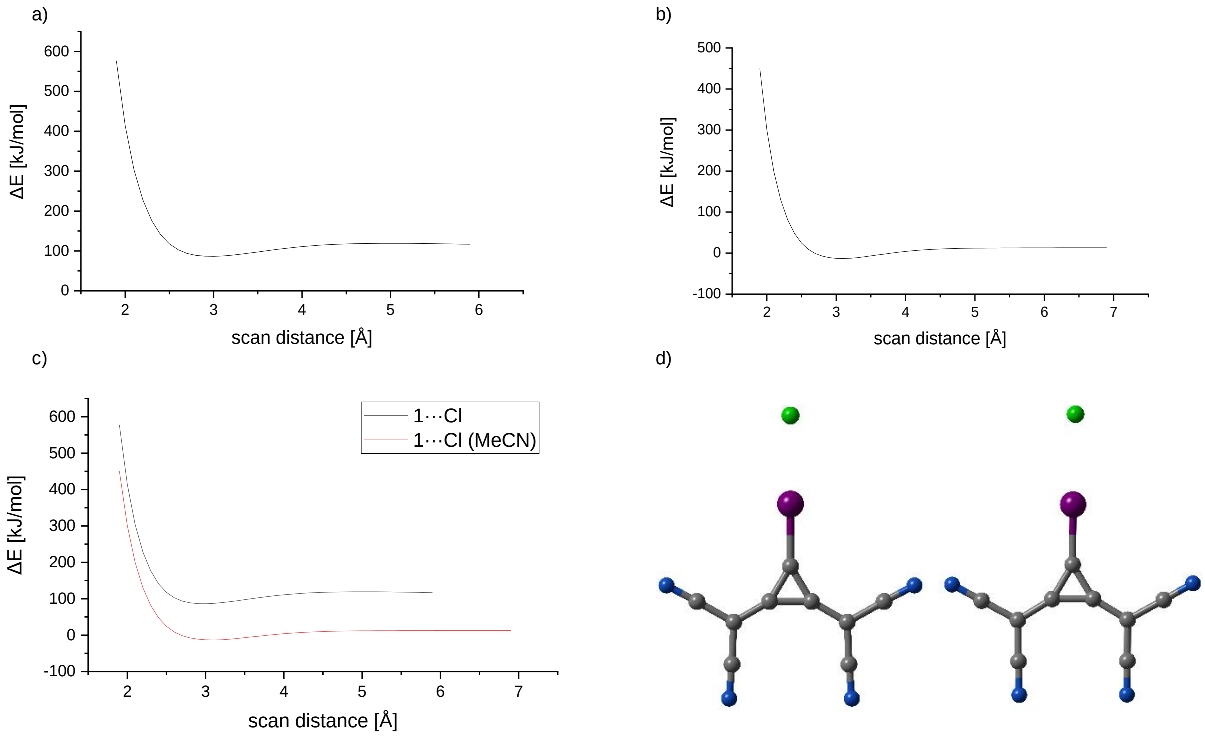

Figure S50. Scan of **1** with chloride in the gas phase (a) and in acetonitrile (b). d) showes the minimum structures with $d_{I\text{-}Cl}$ = 3.0 in the gasphase (left) and 3.1 Å in acetonirtrile (right) and an equally linear arrangement for both calculations.

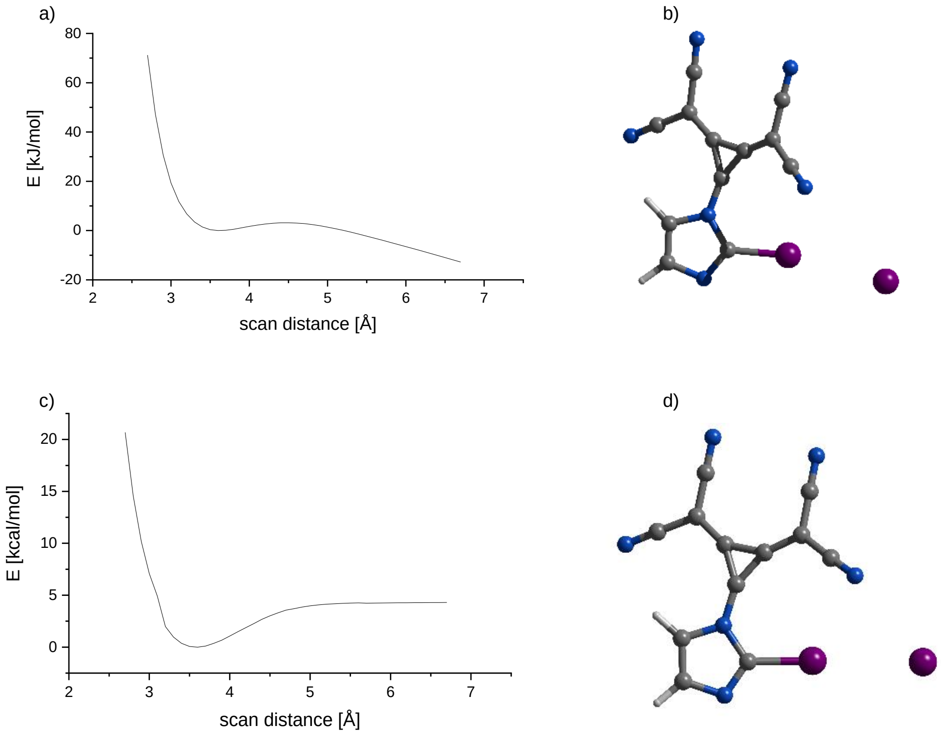

Figure 51. Scan of **6** with iodide in the gas phase (a) and in acetonitrile (c). b) and d) show the corresponding minimum structures.

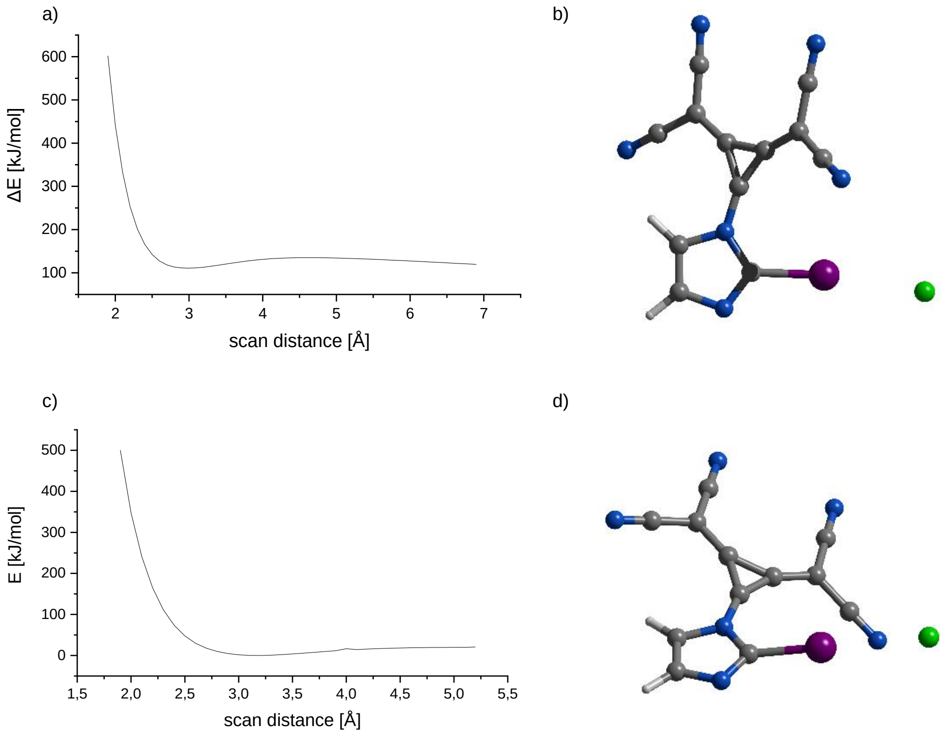

Figure S52. Scan of **6** with chloride in the gas phase (a) and in acetonitrile (c). b) and d) show the corresponding minimum structures.

a) b)

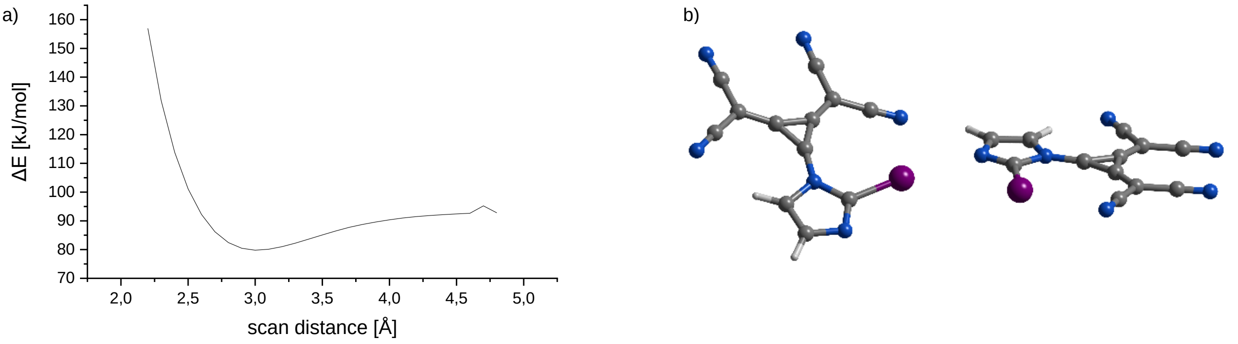

c) d)

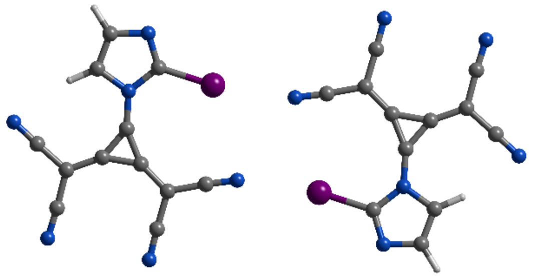

	**6**···**6**1XB,scan	**6**···**6**1XB,crystal	**6**···**6**2XB,crystal
d_{I-N} [Å]	3.0	3.0	3.0
∡$_{C-I-N}$ [°]	173.6	173.6	170.7
∡$_{C-N-C-C}$ [°]	19.1	19.0	2.7
∡$_{C-N-C-C}$ [°]	-3.2	-3.1	2.7
d_{N-H} [Å]	2.4	2.4	-

Figure S53. Dimers of **6**···**6** with one (scan profile for the dimer **6**···**6**$_{1XB}$ (a)), b) structure of the dimer at the energy minimum found in the scan calculation) or two XB contracts (c). Since the scan of the dimeric structure with two XBs could not be completed, we performed an additional optimisation for **6**···**6**$_{1XB}$ on the basis of the coordinates found in the crystal structure. As result, we noticed that we obtained an almost identical geometry as well as energies for gor both calculations (see table). Therefore in case of **6**···**6**$_{2XB}$ we also used the XRD-strucuture coordinates for further optimisation.

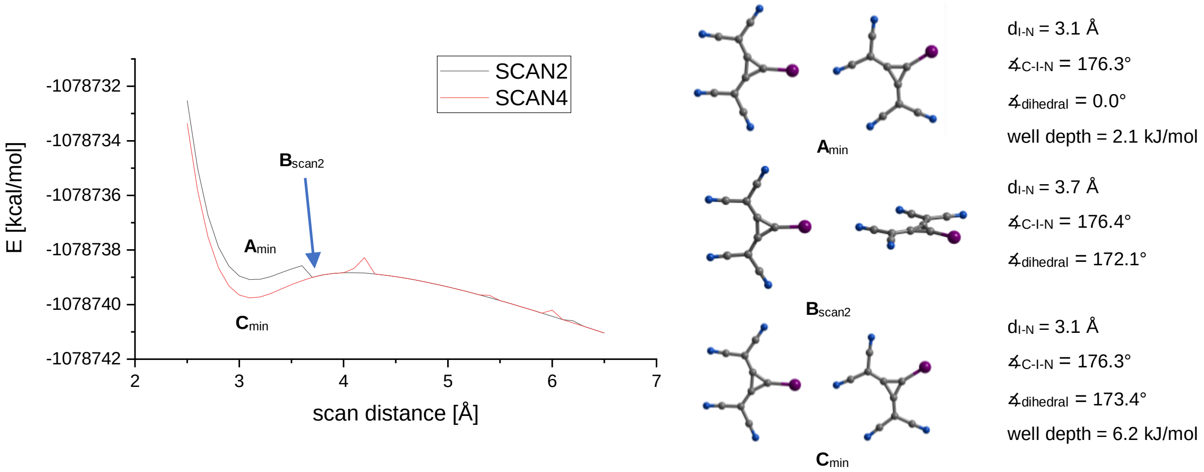

Figure S54. The energy profile observed for the dimer **1**···**1** in the gas phase. For scan 2 (black) the two molecules were originally arranged in one plane, whereas in the second meassurement, the XB-acceptor molecule was already positioned out of plane at the starting positon d = 2.5 Å. The linear arrangement, in which both C-I achis point in one direction was only obtained for dimer **1**···**1** in solution.

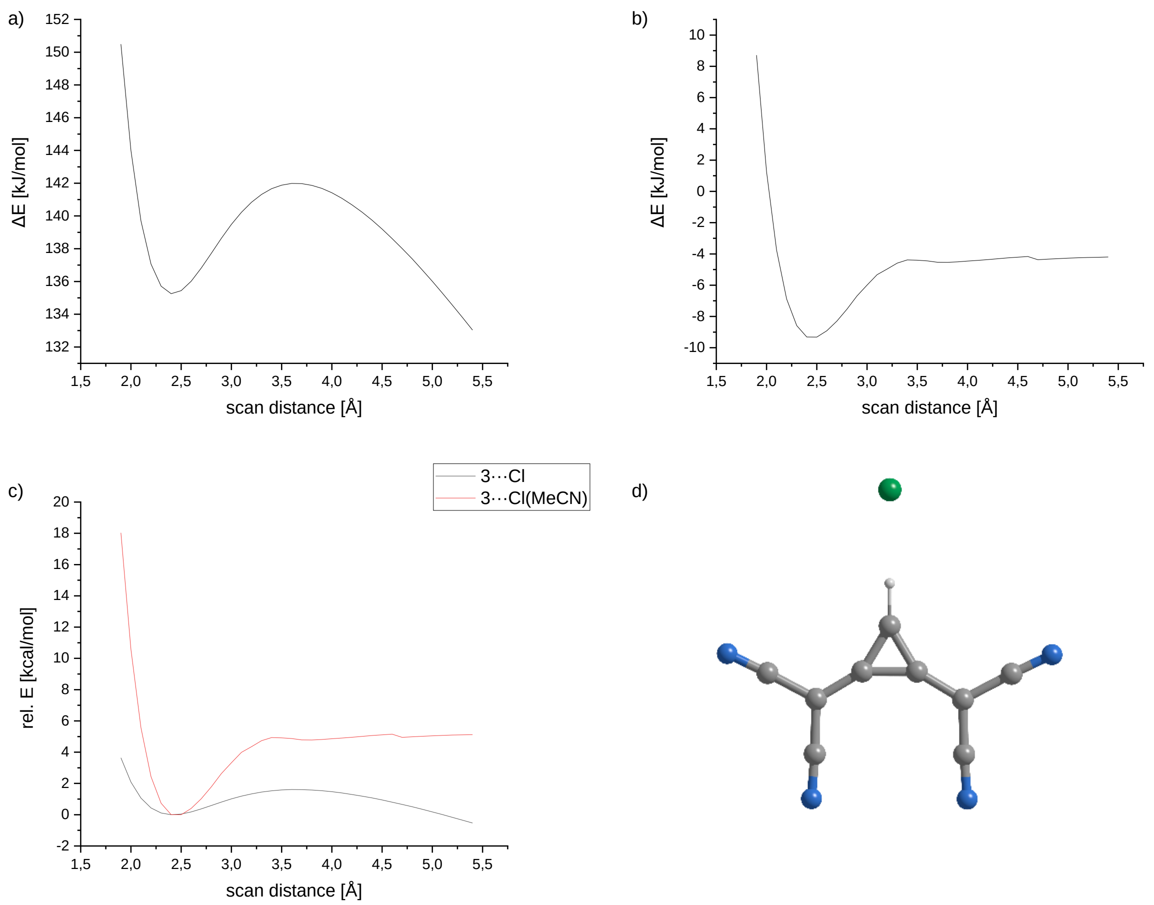

Figure S55. Scan of **3** with chloride in the gas phase (a) and in acetonitrile (b). c) relative energy for both the scan in the gas phase (black) and in acetonitrile (red) demonstrating the alterned energy profile. d) Minimum structure obtained for the scans in the gasphase (almost identical to the minimum structure in acetonitrile).

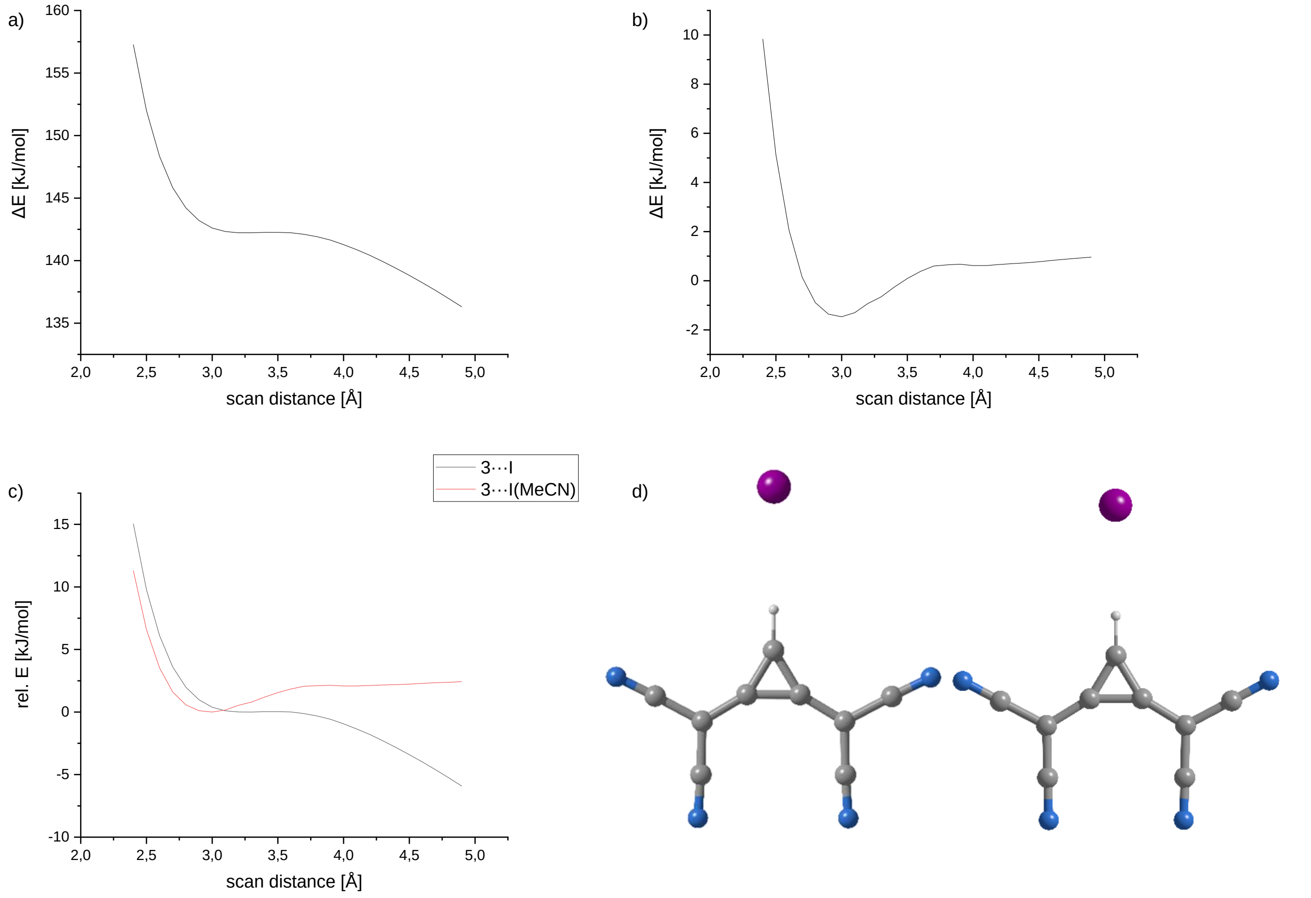

Figure S56. Scan of **3** with iodide in the gas phase (a) and in acetonitrile (b). c) relative energies for both the gasphase calculation and the scan in acetonirtile demonstrating the differences in the energy profiles. d) showes the minimum structures with $d_{H\text{-}I}$ = 3.3 in the gasphase (left) and 3.0 Å in acetonirtrile (right),

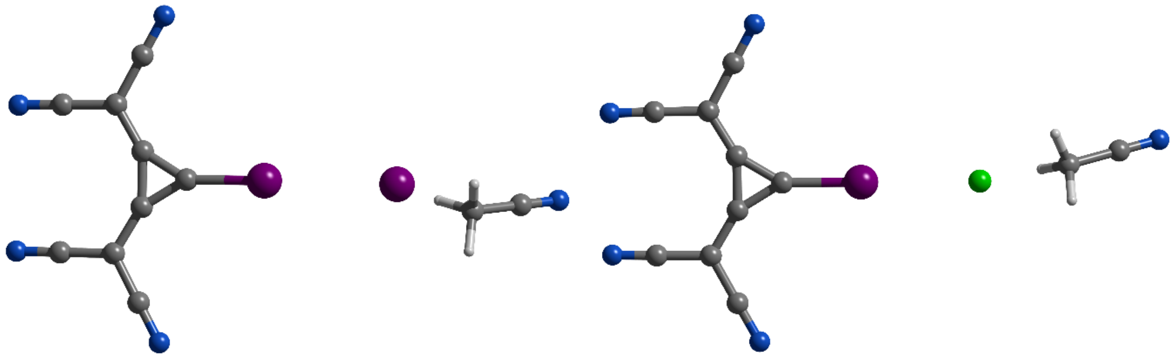

Figure S57. Structures obtained for the implicit solvent calculation of both, [**1**···Cl$^-$···MeCN] and [**1**···I$^-$···MeCN].

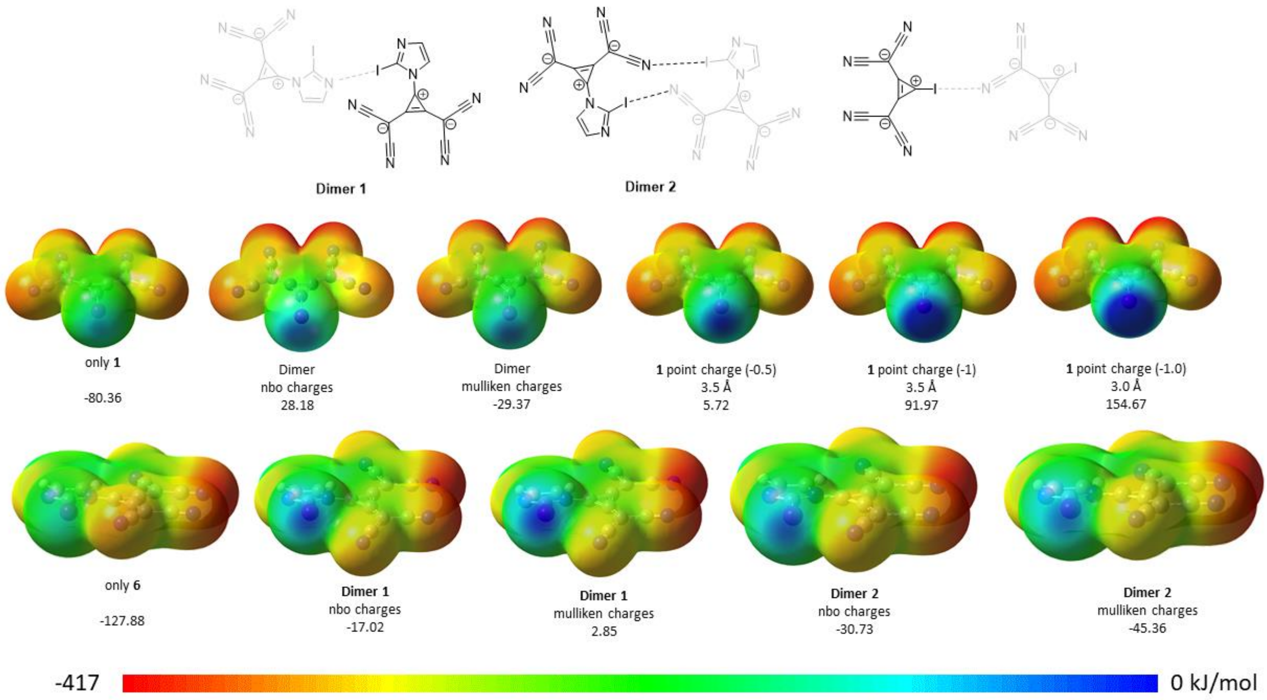

Figure S58. To investigate the role of polarisation, we replaced the atomes of the XB-accepror in the dimers, which were optimised beforhand, with point charges (nbo or mulliken charges) and performed another calculation without structure optimisation this time. In case of iodine compound **1** we used the planaer dimer, although it was found to be higher in energy in the gas phase, since we also found a planar structure experimentally. For **1** also single point charges were used. Afterwards the electrostatic potential was plotted (on the 0.001 au isosurface), Additially the σ-hole energies ($V_{S,max}$) are given in kJ/mol.

Table S20. Geometric parameters (I/H···LB distances in Å, $\measuredangle_{C\text{-}I/H\cdots LB}$ angles in °), well depths, binding energies and Gibb's free energies (with low-frequency entropy corrections)[15] and enthalpies (for the gas phase calculations) [all in kJ/mol] for minima corresponding to halogen bonding adducts. Calculations were performed in the gas phase, unless stated otherwise ("solv") and the corresponding cations were omitted.

	complex	$d_{I/H\cdots LB}$	$\measuredangle_{C\text{-}I/H\cdots LB}$	well depth	ΔE	ΔG	ΔH
1	**1**···I^-	3.5	180	-16	108	136	106
2	**1**···Cl^-	3.0	180	-33	87	117	87
3	**1**···I^-_{solv}[a]	3.5	178	-23	-10	0.6	
4	**1**···Cl^-_{solv}[a]	3.1	180	-26	-14	-1.9	
5	**6**···Cl^-	3.0	171	-24	111	142	111
6	**6**···I^-	3.6	157	-3.0	126	158	
7	**6**···I^-_{solv}[a]	3.6	176	-18	6.7	11	
8	**6**···Cl^-_{solv}[a]	3.0	176	-21	6.7	5.7	
9	**6**···**6**$_{1XB}$[b]	3.0	174	-13	80	141	85
10	**6**···**6**$_{2XB}$[c]	3.0	171	-	89	152	94
11	**1**···**1**	3.1	177	-2.1	101	154	105
12	**1**···**1**$_{solv}$[a]	3.0	180	-14	-14	45	
13	**3**···I^-	3.3	180	-0.03	142	173	144
14	**3**···Cl^-	2.4	180	-6.7	135	166.4	136
15	**3**···I^-_{solv}[a]	3.0	180	-2.1	-1.5	-53	
15	**3**···Cl^-_{solv}[a]	2.5	180	-5.2	9.3	-41	
17	**1**···$Cl^-_{impl.solv}$[d]	3.1	179	-	-7.6	20	
18	**1**···$I^-_{impl.solv}$[d]	3.5	180	-	-14	41	

[a] Calculated with SMD18 using parameters for acetonitrile. [b] This dimeric structure features one XB-contact as found for A···C in the crystal structure of **6a** (see figure 5) [c] A scan for the dimeric structure as found in the crystal structure of **6b** (see figure 4) was not possible. In case of **6**···**6**$_{1XB}$, almost identical energies were obtained when calculations were performed either on the crystal structure geometry or the optimized minimum. Therefore, we used the geometries found in the crystal structure as minimum structure for further optimisation and energy calculation. [d] The calculations used the SMD model plus an additional acetonitrile molecule. In this case the correction factor 6.4 kJ/mol was taken into account, as described elsewhere.[7]

V. References

[1] a) T. Fukunaga, *J. Am. Chem. Soc.* **1976**, *98*, 610; b) T. Fukunaga, US4005091, **1974**.
[2] M. Meyer, W. Paciorek, A. Kowalski, A. Muszynski, A. Wisniewski, M. Pol, M. Przewozniczek, P. Stec, D. Bujnik, H. Kulza et al., *CrysAlisPro*, Rigaku Oxford Diffraction (1995-2018), **2018**.
[3] L. J. Farrugia, *J. Appl. Crystallogr.* **1999**, *32*, 837.
[4] a) G. M. Sheldrick, *Acta Crystallogr., Sect. A, Found. Crystallogr.* **2008**, *64*, 112; b) C. B. Hübschle, G. M. Sheldrick, B. Dittrich, *J. Appl. Crystallogr.* **2011**, *44*, 1281.
[5] H. Putz, K. Brandenburg, "Crystal Impact", zu finden unter http://www.crystalimpact.com/diamond.
[6] A. V. Marenich, C. J. Cramer, D. G. Truhlar, *J. Phys. Chem. B* **2009**, *113*, 6378.
[7] E. Engelage, N. Schulz, F. Heinen, S. M. Huber, D. G. Truhlar, C. J. Cramer, *Chem. Eur. J.* **2018**, *24*, 15983.
[8] M. J. Frisch, G. W. Trucks, H. B. Schlegel, G. E. Scuseria, M. A. Robb, J. R. Cheeseman, G. Scalmani, V. Barone, B. Mennucci, G. A. Petersson et al., *Gaussian 09 Revision E01*, Gaussian, Inc, Wallingford, CT, USA, **2009**.
[9] Y. Zhao, D. G. Truhlar, *Theor. Chem. Acc.* **2008**, *120*, 215.
[10] F. Weigend, R. Ahlrichs, *Phys. Chem. Chem. Phys.* **2005**, *7*, 3297.
[11] D. Rappoport, F. Furche, *J. Chem. Phys.* **2010**, *133*, 134105.
[12] E. Engelage, D. Reinhard, S. M. Huber, *Chem. Eur. J.* **2019**.
[13] S. Grimme, *Chem. Eur. J.* **2012**, *18*, 9955.
[14] J. Contreras-García, E. R. Johnson, S. Keinan, R. Chaudret, J.-P. Piquemal, D. N. Beratan, W. Yang, *J. Chem. Theory Comput.* **2011**, *7*, 625.
[15] S. Grimme, J. Antony, S. Ehrlich, H. Krieg, *J. Chem. Phys.* **2010**, *132*, 154104.
